# Exploitation of the Ugi 5-Center-4-Component Reaction (U-5C-4CR) for the Generation of Diverse Libraries of Polycyclic (Spiro)Compounds

**DOI:** 10.3389/fchem.2018.00369

**Published:** 2018-09-06

**Authors:** Lisa Moni, Fabio De Moliner, Silvia Garbarino, Jörn Saupe, Christian Mang, Andrea Basso

**Affiliations:** ^1^Dipartimento di Chimica e Chimica Industriale, Università degli Studi di Genova, Genova, Italy; ^2^AnalytiCon Discovery GmbH, Potsdam, Germany

**Keywords:** multicomponent reactions, diversity oriented synthesis, scaffold diversity, combinatorial libraries, isocyanides

## Abstract

An Ugi multicomponent reaction with chiral cyclic amino acids, benzyl isocyanide and cyclic ketones (or acetone) has been exploited as key step for the generation of peptidomimetics. After a straightforward set of elaborations, the peptidomimetics were converted into polycyclic scaffolds displaying two orthogonally protected secondary amines. Libraries of compounds were obtained decorating the molecules through acylation/reductive amination reactions on these functional groups.

## Introduction

Drug research is nowadays suffering from the relatively low number of newly approved medicines with a clear health benefit for patients. One of the pitfalls has been recognized in the inadequate availability of potential new starting points for library generation. Structurally novel scaffolds with new modes of action are therefore highly desirable, and the possibility to assemble them through straightforward, yet complexity generating, procedures is a major challenge.

**Scheme 1:**
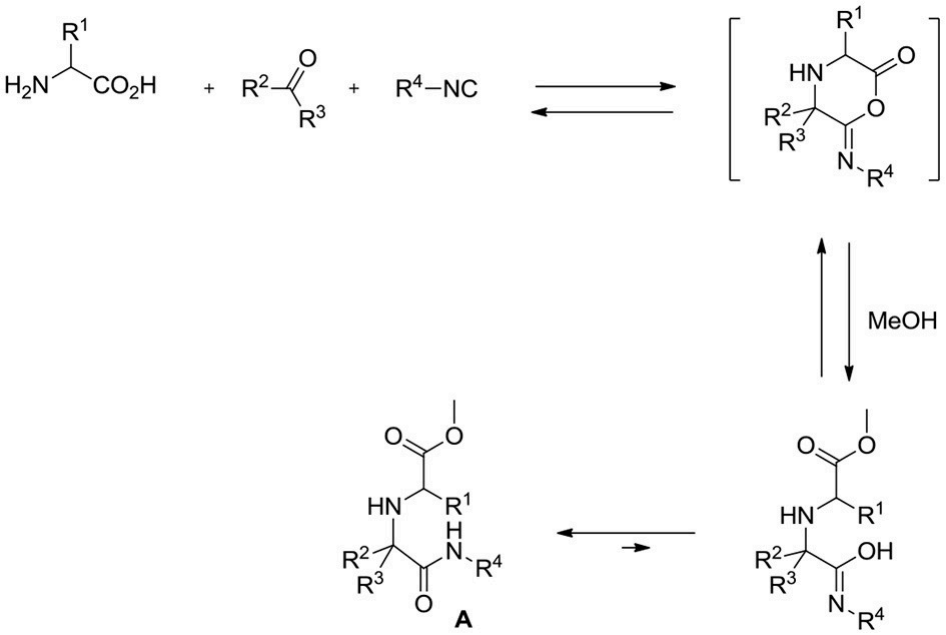
Mechanism of the Ugi 5-center-4-component reaction (U-5C-4CR).

Multicomponent reactions (MCRs) (Orru and de Greef, 2003; Touré and Hall, 2009) are a very powerful tool in the hands of organic chemists, enabling them to assemble complex scaffolds from relatively simple building blocks in just one synthetic step. Among MCRs, isocyanide (Koopmanschap et al., 2014; Giustiniano et al., 2017)-based ones, and specifically the Ugi reaction (U-4CR) (Dömling and Ugi, 2000; Dömling, 2006), are by far the most versatile and exploited ones, also because different variants are available. One of these is the so-called Ugi-5-center-4-component reaction (U-5C-4CR) that, employing α (Demharter et al., 1996; Ugi et al., 1996; Park et al., 1998; Kim et al., 2001; Zimmer et al., 2001; Liu and Dömling, 2009; Mandai et al., 2011; Mimura et al., 2015)- or β (Basso et al., 2004, 2005, 2010)-amino acids as bifunctional reagents, generates α,α′-imino dicarboxylic acid derivatives **A** according to the general mechanism depicted in Scheme 1.

**Scheme 2:**
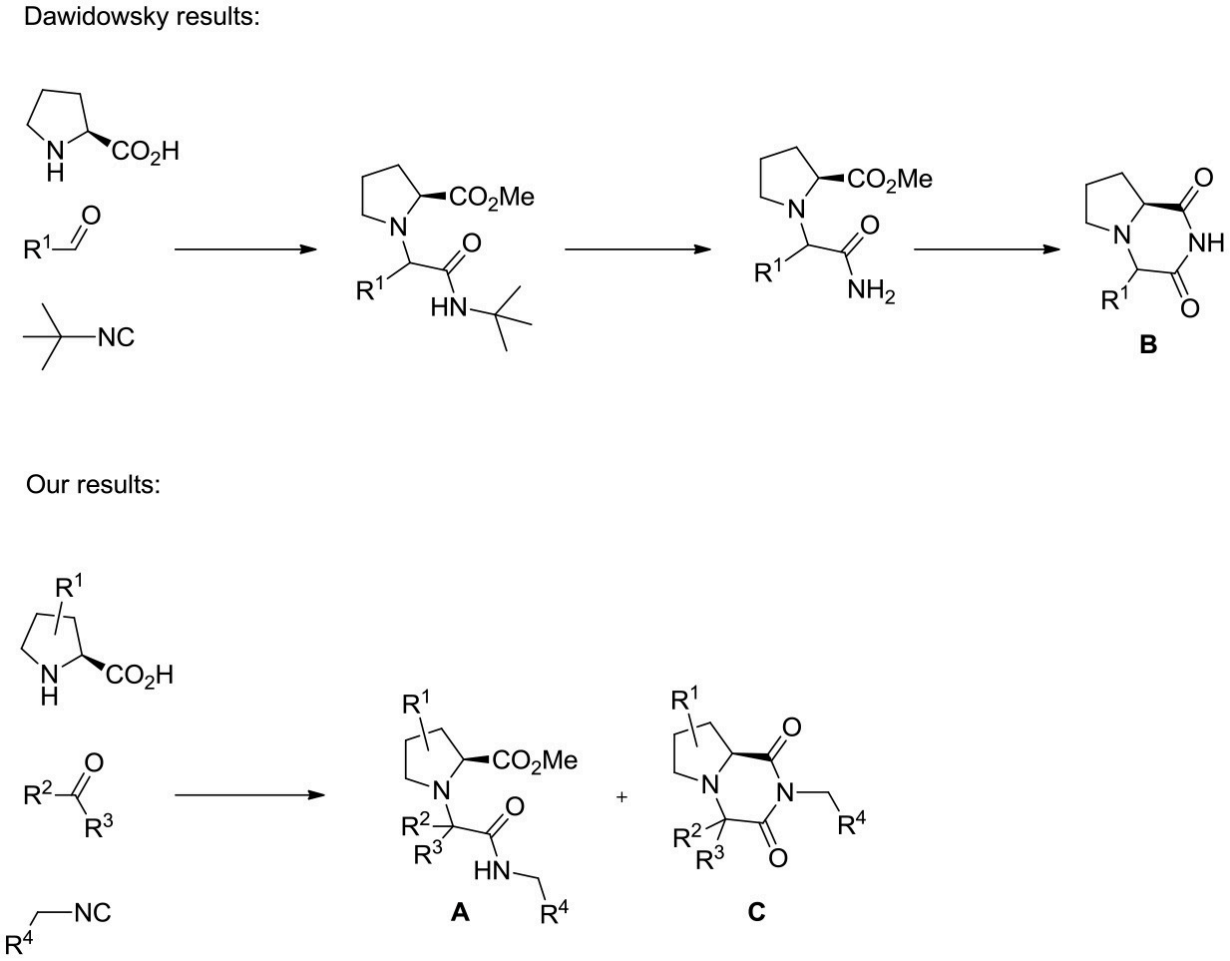
Different outcomes of the reaction between proline derivatives, carbonyl compounds and isocyanides.

The U-5C-4CR has been recently used by Dawidowski (Dawidowski et al., 2012) to prepare 2,6-diketopiperazines **B** starting from proline as aminoacid component, exploiting an intramolecular transamidation between the primary amido group deriving from the cleavage of the *tert*-butyl isocyanide side-chain and the methyl ester group generated during the multicomponent step (Scheme 2). In our hands, when the carbonyl component of the multicomponent step was a ketone, cyclization of **A** to imide **C** partially occurred during the Ugi reaction, even on the secondary amide deriving from unhindered isocyanides (Scheme 2; Dawidowski et al., 2014). With the aim to expand the scope of this chemistry, and due to overexploited synthesis of diketopiperazine libraries for biological applications (Perrotta et al., 2001; Fischer, 2003), we envisioned the possibility to further elaborate structures **C**, through a set of transformations amenable to afford original scaffolds, in optically pure form, with additional reactive groups amenable of selective functionalizations. In this paper we report our results.

## Results and discussion

**Scheme 3:**
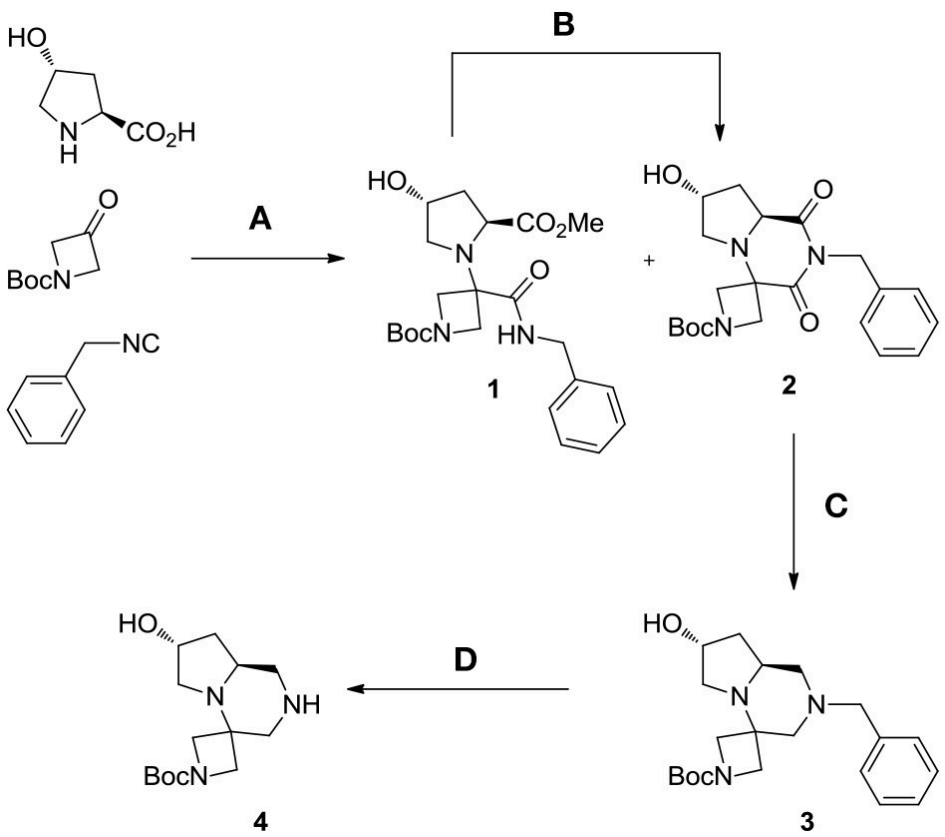
Synthesis of spiro tricyclic scaffold **4**. Reagents and conditions: **(A)** MeOH, 65°C, sealed tube; **(B)** DBU, acetonitrile, rt, 47%; **(C)** borane-Me_2_S complex, THF, 60°C, then MeOH, rt, 76%; **(D)** H_2_ (55 psi), EtOH, rt, quantitative.

Our studies started by investigating the reactivity of *trans*-4-hydroxy-L-proline with *N*-Boc-azetidinone and benzyl isocyanide in methanol (Scheme 3). Different reasons were at the basis of the choice of the carbonyl component: first of all, by using a symmetrical ketone, no additional stereocenters were generated during the Ugi reaction (often showing poor stereoselectivity); then the use of a cyclic ketone allowed us to obtain spiro compounds, privileged structures characterized by high conformational stability; finally, the employment of an *N*-protected azetidinone could be straightforwardly exploited for further functionalizations.

The contemporary presence of a secondary amino group and a cyclic ketone kinetically disfavored the Ugi reaction, which did not proceed at room temperature, even with the addition of a Lewis acid as previously reported by Dawidowski for other substrates (Dawidowski et al., 2014). However, by performing it in a sealed tube at 65°C, a mixture of Ugi adduct **1** and cyclic imide **2** was isolated after 6 days. This mixture was subjected to complete cyclization by addition of a catalytic amount of DBU in acetonitrile. The overall yield after the two steps was an acceptable 47% and remarkably, under these conditions, no epimerization was observed at the L-proline α-carbon, thus allowing us to obtain compound **2** in enantiomerically and diastereomerically pure form.

The subsequent step was the conversion of imide **2** into tertiary amine **3**, exploiting a borane-mediated reduction developed in our laboratories. After careful optimization of the reaction conditions, it was found that, by employing a large excess of borane-dimethylsulfide complex and by heating the mixture at 60°C for 24 h, complete conversion was obtained. From a practical point of view the addition of borane was best performed at 0°C in two aliquots, a white flocculate was observed with the reaction proceeding. Upon completion, slow addition of methanol at 0°C resulted in decomposition of excess borane; however, in order to break complexes between boron and the basic nitrogen atoms of the product, the methanolic solution was left stirring at room temperature overnight.

The crude material was purified by flash chromatography, yielding the desired product **3** in 76% yield. The spiro tricyclic system obtained was the starting point for the generation of a library of compounds. The library generation contemplated the successive deprotection and acylation of the benzyl- and Boc-protected nitrogen atoms, respectively.

Deprotection of the benzyl group was found more difficult than expected and only after 2 days under H_2_ pressure (55 psi) with one replacement of the catalyst (10% Pd/C) after the first day the desired compound **4** was obtained upon filtration, without need of further purification (Scheme 3).

**Scheme 4:**
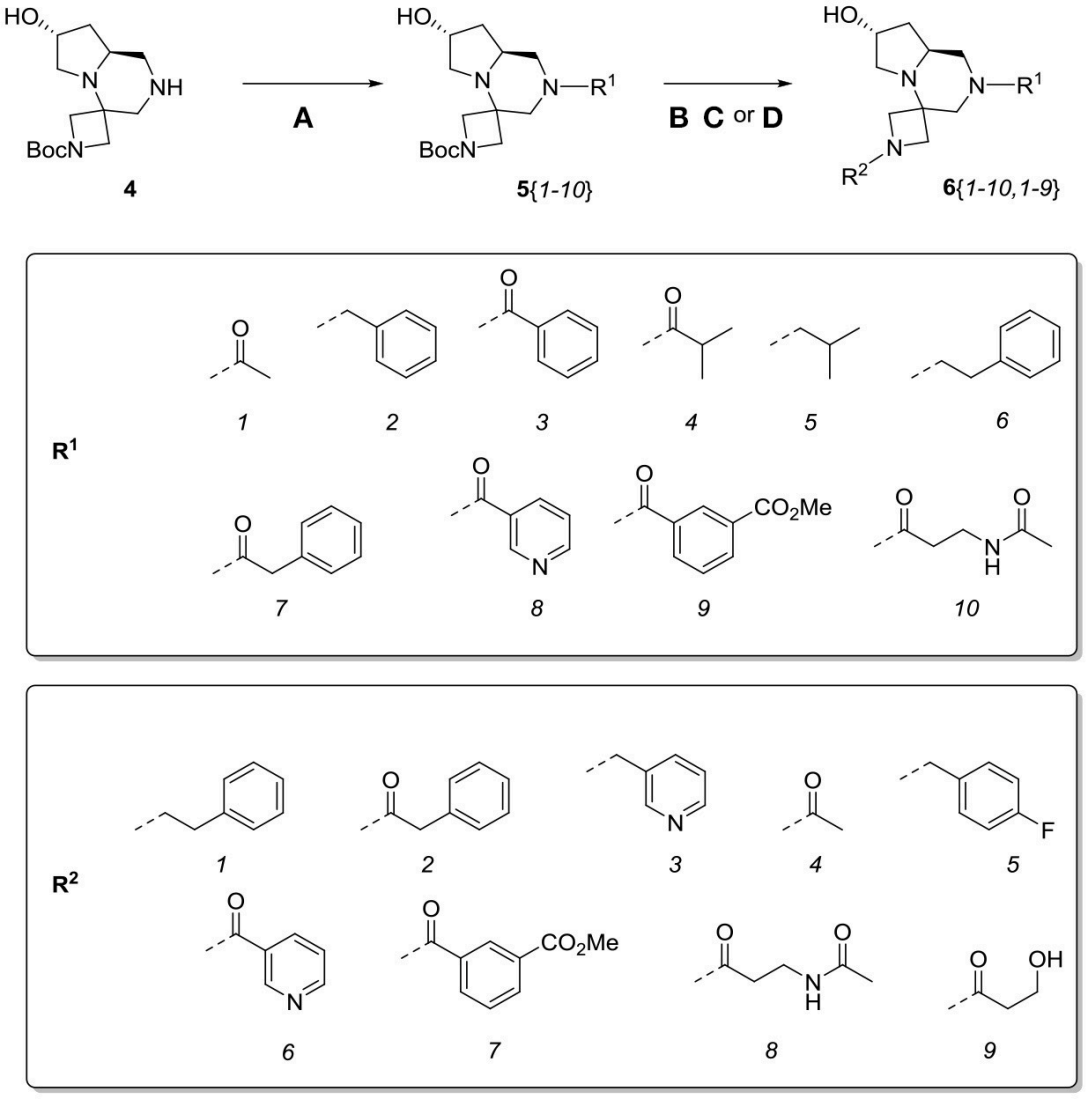
Library generation starting from scaffold **4**. Reagents and conditions: **(A)** acyl chloride, Et_3_N, DCM, rt or carboxylic acid, TBTU, DIPEA, DCM, rt; **(B)** acyl chloride or sulfonyl chloride, Et_3_N, DCM or **(C)** isocyanate, DCM or **(D)** aldehyde/ketone, borane-Me_2_S complex, DCM.

At this stage, reaction with various acyl chlorides or activated carboxylic acids afforded a first set of compounds. No acylation of the secondary alcohol moiety was observed and reactions proceeded usually with 70–80% yield for compounds **5**{*1,3,4,7–10*} (Scheme 4). In order to introduce a higher degree of diversification between the library members, alkylated products **5**{2,5–6} were also included. Compounds **5**{*5–6*} were prepared with an alternative strategy that involved the use, respectively of phenetyl or isobutyl isonitrile in the initial Ugi reaction with hydroxyproline and *N*-Boc-azetidinone. The multicomponent adducts followed the same sequence of transamidation and imide reduction affording directly **5**{*5–6*}. Compound **5**{*2*}, on the other hand, already derived from the same sequence with benzyl isocyanide. All compounds were fully characterized (see experimental part and Data Sheet 1).

With the diversomers **5**{*1–10*} in hand in multigram scale, we proceeded with the introduction of the second diversity input, by cleavage of the Boc group (HCl/MeOH) and elaboration of the free secondary amino group. This was carried out by decoration with acid chlorides, sulfonyl chlorides, isocyanates, aldehydes and ketones using standard conditions affording compound library **6**{*1–10,1–9*} (Scheme 4). The final compounds were obtained in 50–100 mg scale, usually with purities higher than 75% (Table 1).

**Table 1:**
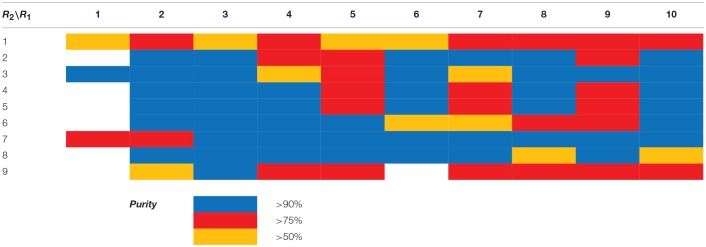
HPLC-UV purities of library members **6**{*1–10,1–9*}.

**Scheme 5:**
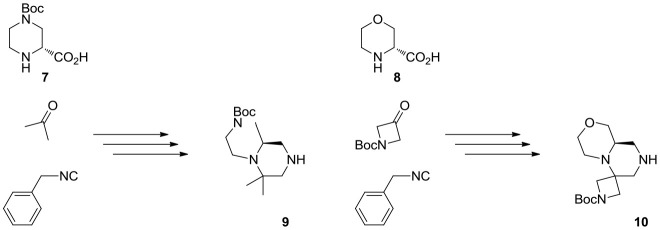
Multicomponent approach to the synthesis of scaffolds **9** and **10**. See experimental part for details.

The overall synthetic methodology was extended to other two enantiomerically pure cyclic amino acids, namely (R)-4-(*tert*-butoxycarbonyl)piperazine-2-carboxylic acid **7** and (R)-morpholine-2-carboxylic acid **8** (Scheme 5). In the first instance, the piperazine ring already possessed an additional Boc-protected amino group, therefore the Ugi reaction was performed with acetone, affording scaffold **9**. In the second case, *N*-Boc-azetidinone was used instead, in analogy with the first library described in this paper.

**Scheme 6:**
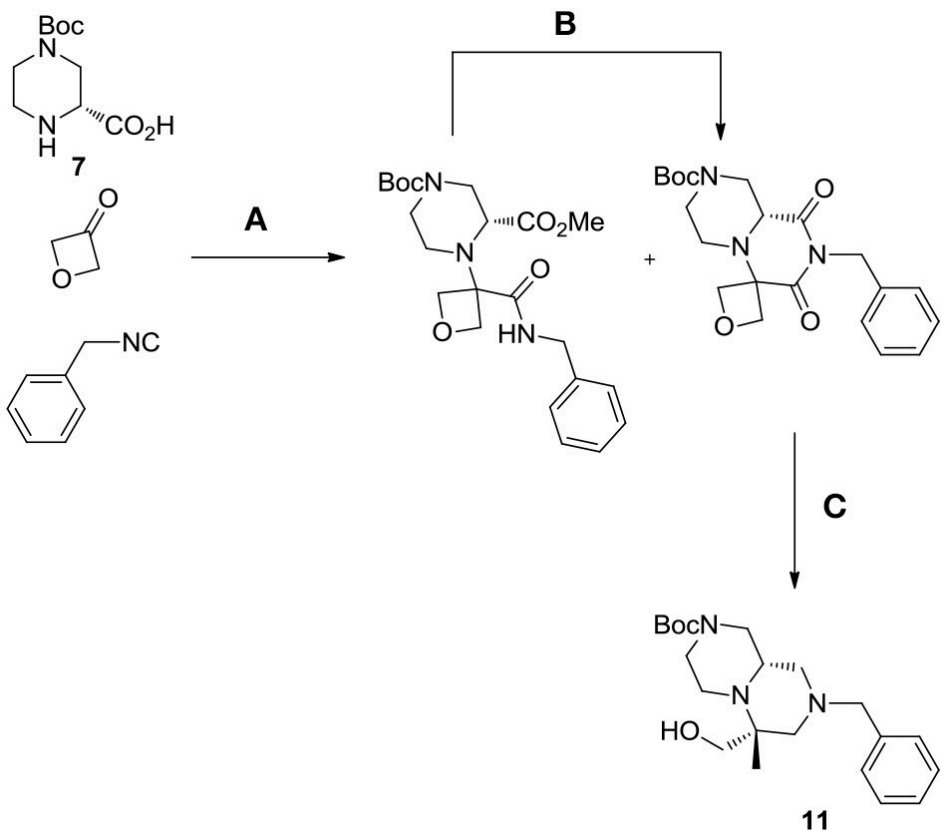
Unexpected outcome of the reaction with oxetanone as carbonyl component. Reagents and conditions: **(A)** MeOH, 65°C, sealed tube; **(B)** DBU, acetonitrile, rt, 54%; **(C)** borane-Me_2_S complex, THF, 60°C, then MeOH, rt, 57%.

Interestingly, in the case of piperazine-2-carboxylic acid **7**, also oxetanone was used as carbonyl component in the Ugi reaction, however, during borane reduction of the imide intermediate, the oxetanone ring was stereoselectively opened, affording derivative **11** (Scheme 6).

**Scheme 7:**
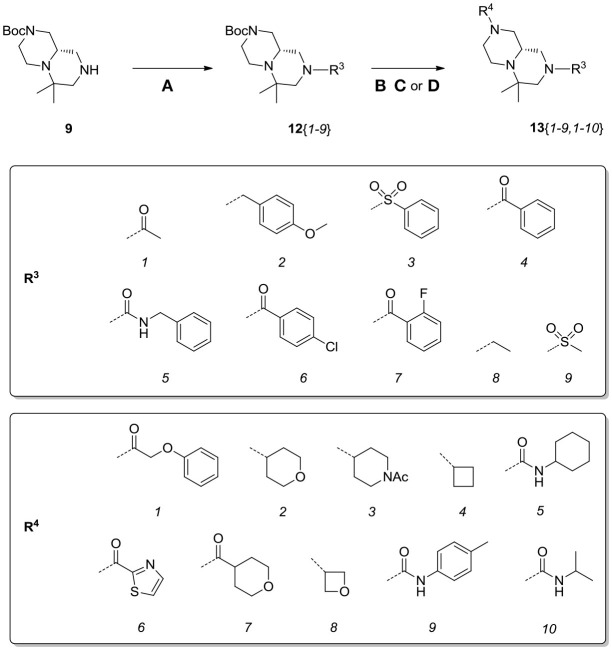
Synthesis of library **13**. Reagents and conditions: **(A)** acyl chloride (or sulfonyl chloride or isocyanate), Et_3_N, DCM, rt; **(B)** acyl chloride or sulfonyl chloride, Et_3_N, DCM or **(C)** isocyanate, DCM or **(D)** aldehyde/ketone, borane-Me_2_S complex, DCM.

**Scheme 8:**
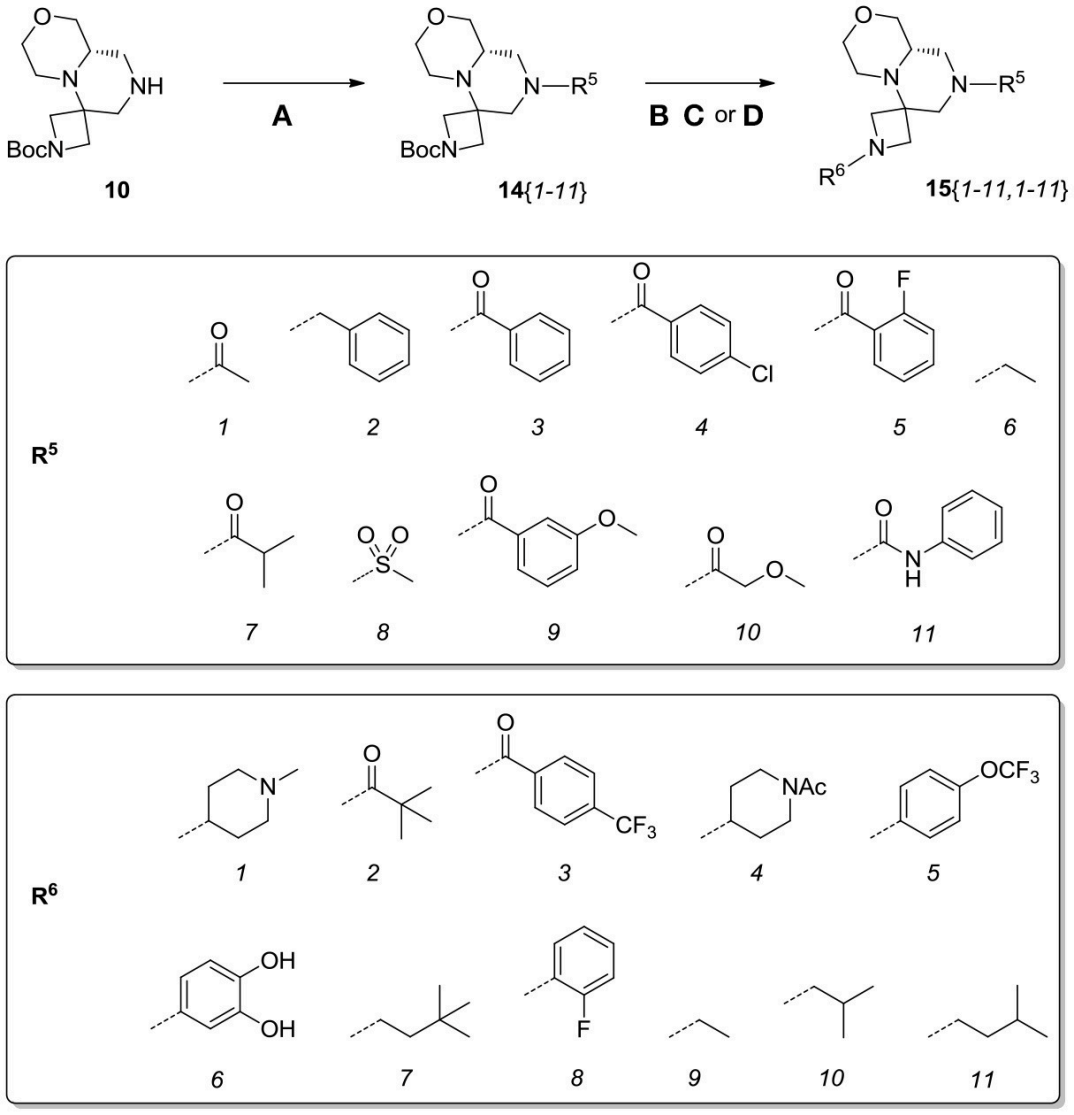
Synthesis of library **15**. Reagents and conditions: **(A)** acyl chloride (or sulfonyl chloride or isocyanate), Et_3_N, DCM, rt; **(B)** acyl chloride or sulfonyl chloride, Et_3_N, DCM or **(C)** isocyanate, DCM or **(D)** aldehyde/ketone, borane-Me_2_S complex, DCM.

Scaffolds **9** and **10** were then subjected to the same decorations procedures of compound **4**, affording libraries **13**{*1–9,1–10*} (Scheme 7 and Table 2) and **15**{*1–11,1–11*} (Scheme 8 and Table 3).

**Table 2:**
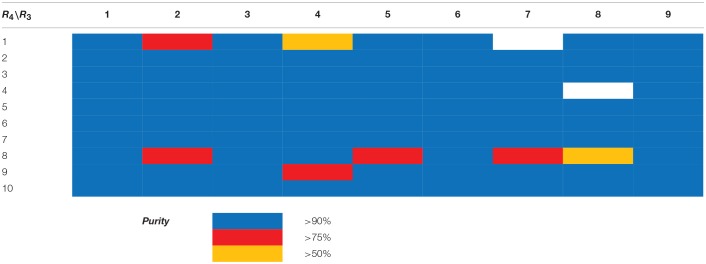
HPLC-UV purities of library members **13**{*1–9,1–10*}.

**Table 3:**
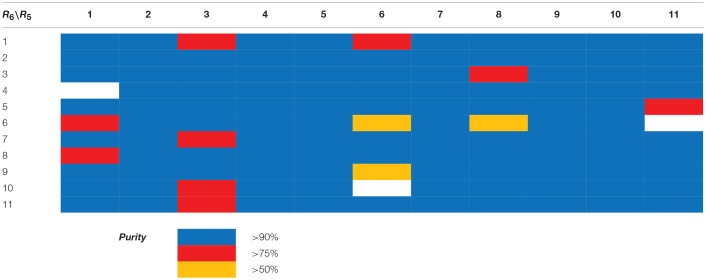
HPLC-UV purities of library members **15**{*1–11,1–11*}.

**Figure 1:**
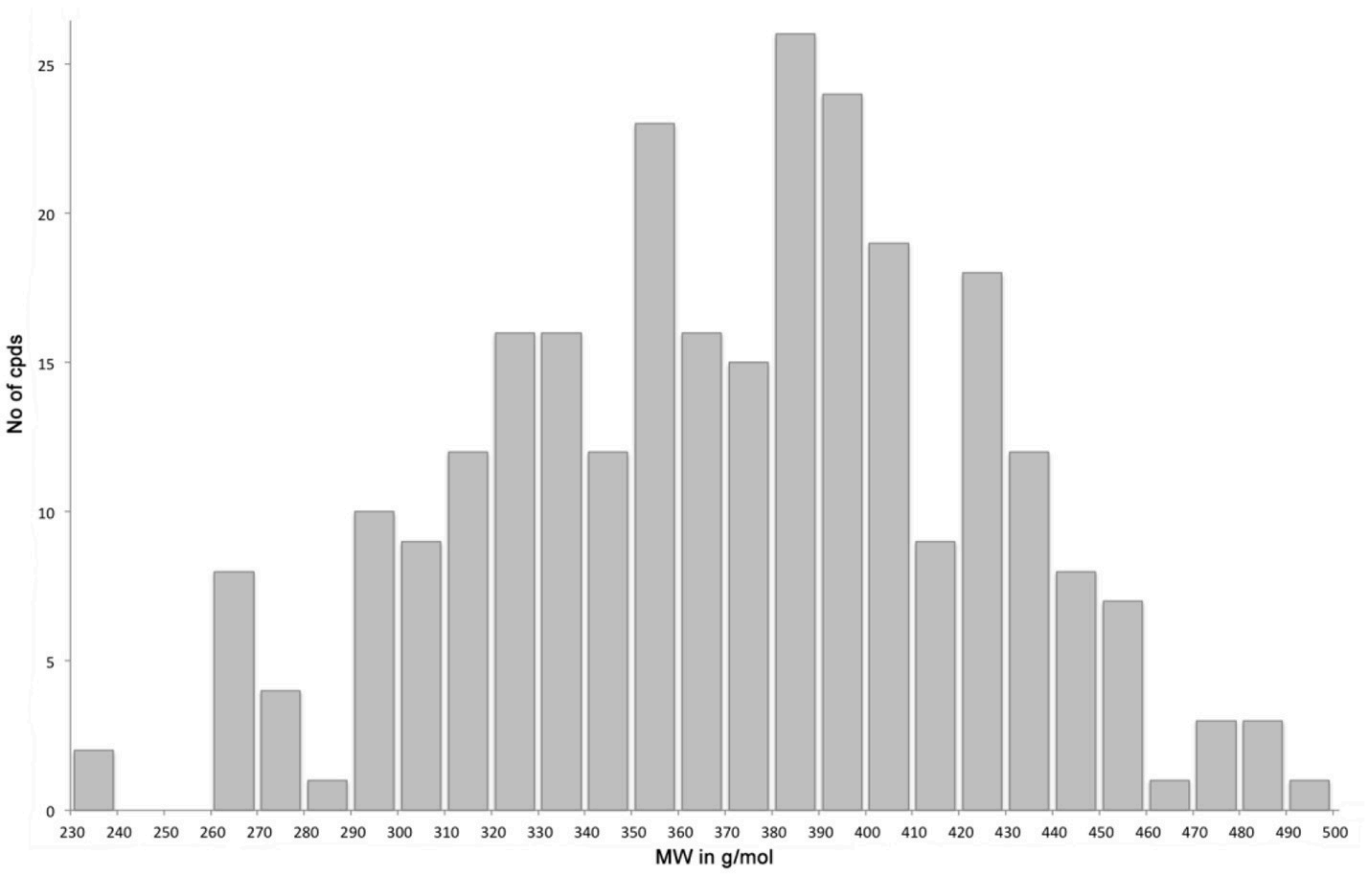
Molecular weight distribution of library members.

**Figure 2:**
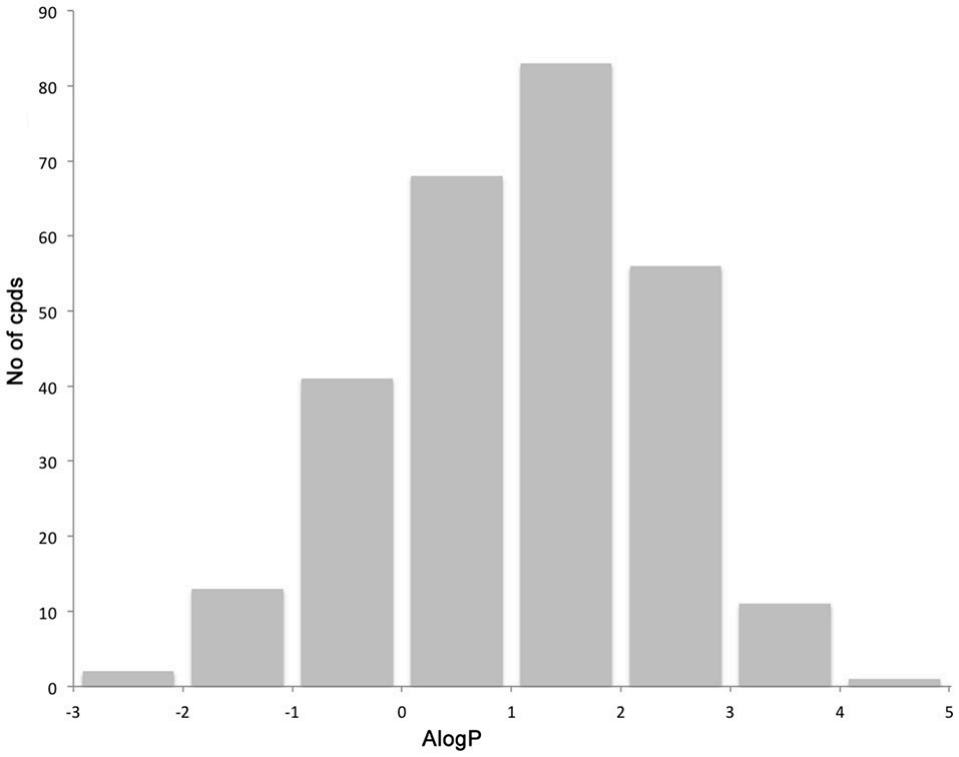
Distribution of the partition coefficient AlogP.

**Figure 3:**
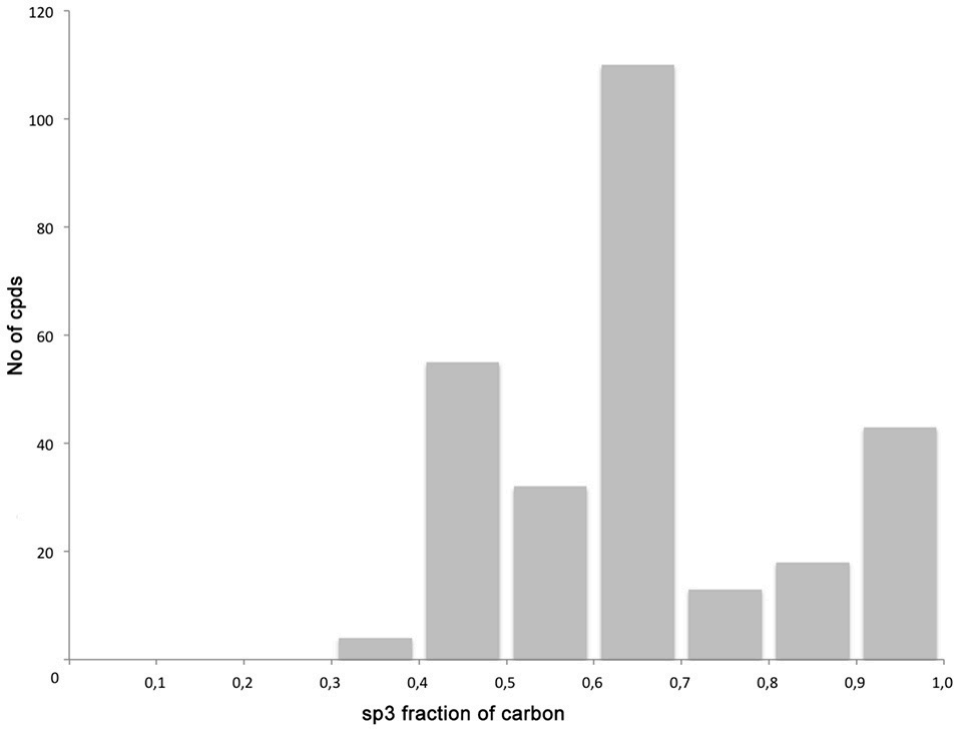
Degree of carbon sp3 fraction.

The library compounds were analyzed using Lead Likeness And Molecular Analysis (Llama) (Colomer et al., 2016). Analyzing the library members revealed that all compound show a molecular weight below 500 g/mol (Figure 1) as well as a partition coefficient AlogP between −3 and 5, with the majority of compounds having a AlogP between −1 and 3 (Figure 2). These values perfectly fit into the Lipinski rule of five, which is an important benchmark to obtain orally available compounds (Lipinski et al., 2001). Also the degree of carbon sp^3^ fraction was analyzed (Figure 3). The remarkably high amount of saturation is translated into a sp^3^ fraction of carbon in the range of 0.3 to 1 with the majority of compounds showing values between 0.4 and 1.

**Figure 4:**
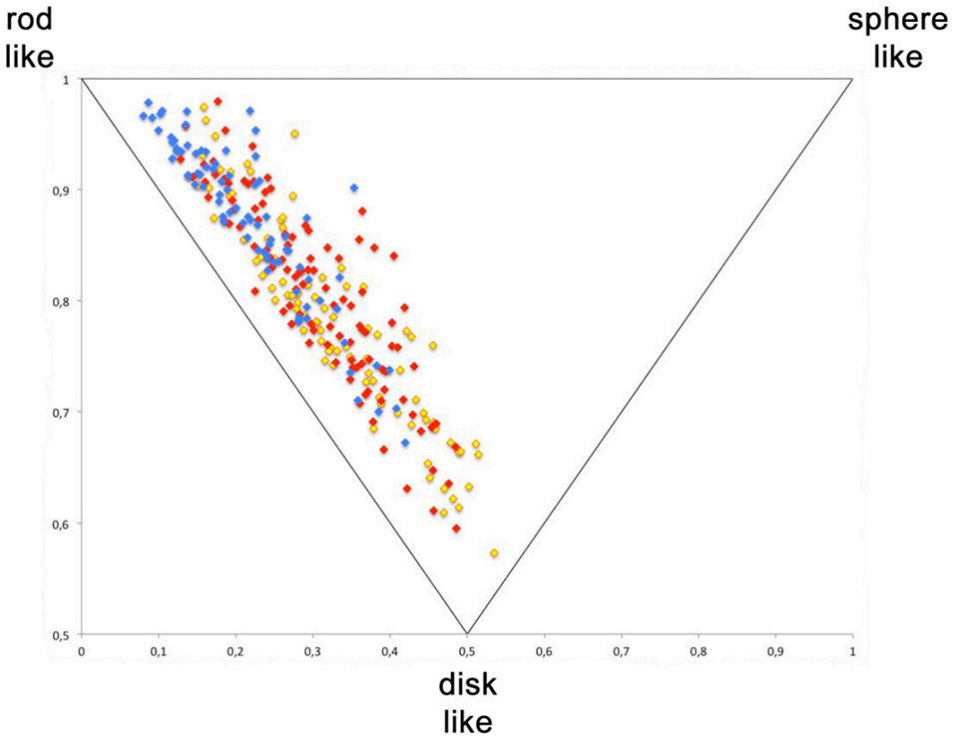
Principle moment of inerta (PMI) analysis. Yellow—library 6, red—library 15, blue—library 13.

For the visualization of the diversity generated with this library, a principal moments of inertia (PMI) analysis was performed (Figure 4). It shows the actual synthesized compounds of the three scaffolds; library 15 in blue, library 10 in red and library 6 in yellow. A broad distribution with the majority of compounds being in the region between rod- and disc-like and a certain portion of library members in the sphere-like region was observed. The library 15 has fewer compounds in the disc like region, compared to the two other libraries 16 and 17. However, the PMI analysis proofs that the Ugi 5-center-4-component reaction allows the generation of compound libraries with a good three-dimensional distribution of compounds.

## Conclusions

In conclusion, we have demonstrated that diverse libraries of polycyclic (spiro)compounds can be efficiently assembled in a straightforward manner, exploiting a multicomponent approach. The potential of the Ugi reaction as a versatile tool for parallel chemistry, even beyond explicitly peptidic space, has been once more evidenced. Parameter analysis of the structurally new libraries described in this work shows that PhysChem values are in a perfect range for pharmacological applications. Moreover, Fsp^3^—a parameter of growing interest in recent years—is addressed in an exemplary fashion.

## Experimental part

### General remarks

NMR spectra were recorded at 300 MHz (^1^H) and 75 MHz (^13^C) and the chemical shifts (δ) are expressed in parts per million relative to tetramethylsilane (TMS) as internal standard (0.00 ppm). Coupling constants are reported in hertz. Unless otherways stated, NMR acquisitions were performed at 295 K and CDCl_3_ was used as solvent.

Following methods were used for the LC/MS spectra (Shimadzu MS2020) of the final compounds:

Method A: a linear gradient was used starting with 5 mM ammonium hydrogen carbonate buffer containing ammonia (pH 10.4), ending with MeOH containing 5 mM ammonium hydrogen carbonate buffer at a flow rate of 1.2 mL/min.
Method B: a linear gradient was used starting with 5 mM ammonium formiate buffer containing 0.1% formic acid, ending with MeOH/ACN/ammonium formiate buffer containing 0.1% formic acid (0.5:0.5:1, v/v/v) at a flow rate of 0.7 mL/min.
Method C: a linear gradient was used starting with 5 mM ammonium formiate buffer containing 0.1% formic acid, ending with MeOH/ACN/ammonium formiate buffer containing 0.1% formic acid (0.5:0.5:1, v/v/v) at a flow rate of 0.7 mL/min and at a oven temperature of 60°C.

All final products with a purity below 90% (UV 215 nm) were purified with preparative HPLC (Knauer K1800 pumps, fitted with a Knauer K2500 UV detector and a Sedere Sedex 75 ELSD). Basic compounds were purified with a Gemini NX column (50 mm × 21.2 mm, 5 μm) using a fitted gradient systems for each compound [solvent A: H_2_O, 1%NH_3_ (aq., 26%)/solvent B: MeOH, 1%NH3(aq., 26%)] at a flow rate of 35 mL/min. Neutral compounds were purified with a Phenomenex LunaC8 column (50 mm × 25 mm, 5 μm) using a fitted gradient system for each compound (solventA: H_2_O, 0.1% AS/solvent B: acetonitrile, 0.1% AS) at a flow rate of 70 mL/min.

HR-MS analyses were carried out on a Synapt G2 QToF mass spectrometer. MS signals were acquired from 50 to 1,200 m/z in ESI positive ionization mode. Reactions were monitored by TLC. TLC analyses were carried out on silica gel plates (thickness = 0.25 mm), viewed at UV (λ = 254 nm) and developed with iodine vapors or Hanessian stain (dipping into a solution of (NH_4_)_4_MoO_4_·4H_2_O (21 g) and Ce(SO_4_)_2_·4H_2_O (1 g) in H_2_SO_4_ (31 mL) and H_2_O (469 mL) and warming). Column chromatographies were performed with the “flash” methodology alternatively using 220–400 mesh silica, grade I alumina or 60–100 mesh Florisil. Solvents employed as eluents and for all other routinary operations, as well as anhydrous solvents and all reagents used were purchased from commercial suppliers and employed without any further purification.

### Synthesis of scaffolds 4, 9, and 10

(7′R,8a′S)-*tert*-butyl 2′-benzyl-7′-hydroxy-1′,3′-dioxohexahydro*-1*′*H-*spiro[azetidine-3,4′-pyrrolo[1,2-*a*]pyrazine]-1-carboxylate **2**


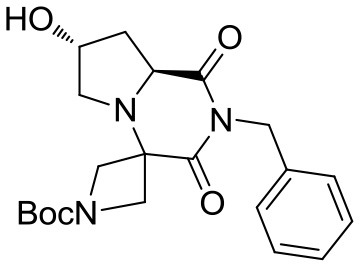


*Trans* 4-hydroxy-L-proline (3.0 g, 23 mmol) was dissolved in methanol (30 mL) within a pressure flask together with *tert*-butyl-3-oxoazetidine-1-carboxylate (3.9 g, 23 mmol) and benzyl isocyanide (2.7 mL, 23 mmol). The flask was then sealed, warmed up to 65°C and left stirring for 6 days. The solution was then allowed to cool down and the solvent removed in vacuo. Crude material was diluited with dichloromethane and washed with 5% sodium bicarbonate solution and brine. The organic layer was dried over sodium sulfate, filtered and concentrated under reduced pressure.

The crude material was dissolved in acetonitrile (30 mL) and DBU (0.34 mL, 2.3 mmol) was added under vigorous stirring at room temperature. After complete consumption of the Ugi adduct the solution was diluted with dichloromethane and washed with saturated ammonium chloride, 5% sodium bicarbonate and brine. The organic layer was dried over sodium sulfate, filtered, concentrated under reduced pressure and purified by flash chromatography (PE/EA 1:1 +2% MeOH), yielding 4.2 g of product (10 mmol, 45% yield) as a yellow foam.

^1^H NMR: 7.35–7.22 (m, 5H), 4.94 (s, 2H), 4.58 (d, *J* = 8.8 Hz, 1H), 4.43 (m, 1H), 4.14 (d, *J* = 8.6 Hz, 1H), 3.87 (m, 3H), 2.93 (qd, *J* = 10.1, 3.6 Hz, 2H), 2.53 (dt, *J* = 14.0, 6.5 Hz, 1H), 2.44 (d, *J* = 5.5 Hz, 1H), 2.28 (ddd, *J* = 14.0, 8.0, 1.7 Hz, 1H), 1.44 (s, 9H).

^13^C NMR: 171.75, 171.30, 156.11, 136.60, 128.80, 128.70, 127.86, 80.56, 69.31, 58.58, 58.46, 55.89, 54.98 (broad), 54.31 (broad), 43.19, 38.71, 28.44.

HR-MS (m/z): [M+H]^+^ calcd for C_21_H_28_N_3_O_5_, 402.2023; found, 402.2015.

(7′R,8a′S)-*tert*-butyl 2′-benzyl-7′-hydroxyhexahydro-*1*′*H*-spiro[azetidine-3,4′-pyrrolo[1,2-*a*]pyrazine]-1-carboxylate **3**


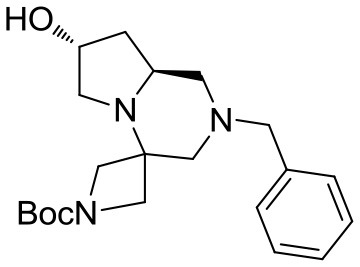


Cyclic imide (2.3 g, 5.7 mmol) was dissolved in THF (25 mL) under nitrogen atmosphere at room temperature. The solution was cooled down to 0°C and borane-dimethylsulfide complex (2 M solution in THF, 14.3 mL, 28.7 mmol) was slowly added in two aliquots with 1 h interval. Once the addition was complete the reaction was warmed up to 60°C and left stirring overnight.

The reaction was then cooled down to 0°C again and methanol (40 mL) was added dropwise in 1 h, then the reaction was left stirring for 1 day to allow complete decomposition of boranes. The solvents were then removed under reduced pressure and the crude material was purified by flash chromatography (PE/EA 4:6) affording the product (1.7 g, 4.7 mmol) in 82% yield as a colorless oil.

^1^H NMR: 7.36–7.23 (m, 5H), 4.48 (m, 1H), 4.18–4.03 (d, *J* = 9.3 Hz, 1H), 3.89 (d, *J* = 9.3 Hz, 1H), 3.68–3.64 (m, 2H), 3.57–3.40 (m, 4H), 2.97–2.81 (m, 2H), 2.70 (m, 1H), 2.52 (dd, *J* = 9.7, 4.3 Hz, 1H), 2.11 (d, *J* = 10.8 Hz, 1H), 1.79 (t, *J* = 10.2 Hz, 1H), 1.74–1.64 (m, 2H), 1.43 (s, 9H).

^13^C NMR: 156.13, 138.24, 128.94, 128.48, 127.32, 79.70, 69.45, 62.88, 62.51, 60.90, 57.20, 55.66, 55.61, 55.07, 39.32, 30.02, 28.54.

HR-MS (m/z): [M+H]^+^ calcd for C_21_H_32_N_3_O_3_, 374.2438; found, 374.2435.

(7′R,8a′S)-*tert*-butyl 7′-hydroxyhexahydro-*1*′*H*-spiro[azetidine-3,4′-pyrrolo[1,2-*a*]pyrazine]-1-carboxylate **4**


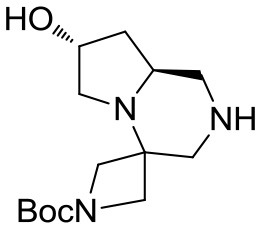


The reaction was performed in a Parr Shaker Hydrogenation apparatus. *N*-benzyl tertiary amine (3.8 g, 10 mmol) was dissolved im methanol (50 mL) under argon atmosphere and 10% Pd/C (0.6 g) was added. The inert atmosphere was then replaced by hydrogen gas (55 psi) and the suspension mechanically shaken for 2 days. Hydrogen atmosphere was then removed, the solution was filtered over a short celite pad and concentrated under reduced pressure. The crude material was recovered in quantitative yield and used for the final acylations without further purification.

^1^H NMR: 4.52–4.43 (m, 1H), 4.09 (d, *J* = 9.3 Hz, 1H), 3.93 (d, *J* = 9.6 Hz, 1H), 3.66 (d, *J* = 9.0 Hz, 1H), 3.60–3.50 (m, 2H), 3.09 (d, *J* = 12.0 Hz, 1H), 3.04 (dd, *J* = 11.7, 2.1 Hz, 1H), 2.71 (dd, *J* = 12.0, 1.8 Hz, 1H), 2.60–2.35 (m, 3H), 1.80–1.60 (m, 2H), 1.45 (s, 9H).

^13^C NMR: 156.51, 79.80, 68.92, 58.63, 56.52, 56.20, 55.79, 54.28, 50.44, 39.48, 30.00, 28.54.

HR-MS (m/z): [M+H]^+^ calcd for C_14_H_26_N_3_O_3_, 284.1969; found, 284.1953.

### Synthesis of scaffold 9

(R)-*tert*-butyl 8-benzyl-6,6-dimethyl-7,9-dioxohexahydro-*1H*-pyrazino[1,2-*a*]pyrazine-2(*6H*)-carboxylate


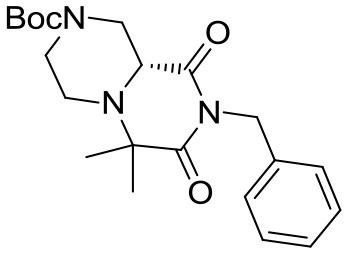


(R)-4-(*tert*-butoxycarbonyl)piperazine-2-carboxylic acid (5.8 g, 25 mmol) was dissolved in methanol (50 mL) within a pressure flask together with acetone (3.5 mL, 50 mmol) and benzyl isocyanide (2.9 mL, 24 mmol). The flask was then sealed, warmed up to 65°C and left stirring for 7 days. The solution was then allowed to cool down and the solvent removed in vacuo. Crude material was diluited with dichloromethane and washed with 5% sodium bicarbonate solution and brine. The organic layer was dried over sodium sulfate, filtered and concentrated under reduced pressure.

The crude material was dissolved in acetonitrile (20 mL) and DBU (0.37 mL, 2.5 mmol) was added under vigorous stirring at room temperature. After complete consumption of the Ugi adduct the solution was diluted with dichloromethane and washed with saturated ammonium chloride, 5% sodium bicarbonate and brine. The organic layer was dried over sodium sulfate, filtered, concentrated under reduced pressure and purified by flash chromatography (PE/EA 1:1), yielding 3.6 g of product (9.4 mmol, 39% yield) as a yellow foam.

^1^H NMR: 7.34–7.20 (m, 5H), 4.98 (d, *J* = 14.0 Hz, 1H), 4.89 (d, *J* = 14.0 Hz, 1H), 4.44 (broad d, *J* = 13.0 Hz, 1H), 3.93 (d, *J* = 13.0 Hz, 1H), 3.39 (dd, *J* = 9.3, 3.7 Hz, 1H), 3.15–2.75 (m, 3H), 2.39 (td, *J* = 10.9, 3.2 Hz, 1H), 1.52 (s, 3H), 1.47 (s, 9H), 1.22 (s, 3H).

^13^C NMR: 174.46, 169.42, 154.41, 136.84, 128.60, 128.53, 127.61, 80.47, 61.12, 57.35, 45.52, 44.39, 43.21, 28.50, 23.98, 16.87.

HR-MS (m/z): [M+H]^+^ calcd for C_21_H_30_N_3_O_4_, 388.2231; found, 388.2212.

(S)-*tert*-butyl 8-benzyl-6,6-dimethylhexahydro-*1H*-pyrazino[1,2-*a*]pyrazine-2(*6H*)-carboxylate


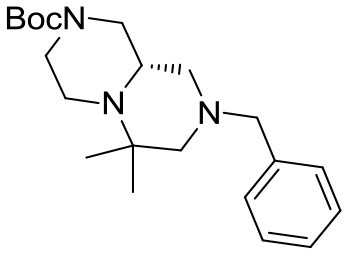


Cyclic imide (2.3 g, 5.9 mmol) was dissolved in THF (26 mL) under nitrogen atmosphere at room temperature. The solution was cooled down to 0°C and borane-dimethylsulfide complex (2 M solution in THF, 15.0 mL, 29.5 mmol) was slowly added in two aliquots with 1 h interval. Once the addition was complete the reaction was warmed up to 60°C and left stirring overnight.

The reaction was then cooled down to 0°C again and methanol (40 mL) was added dropwise in 1 h, then the reaction was left stirring for 1 day to allow complete decomposition of boranes. The solvents were then removed under reduced pressure and the crude material was purified by flash chromatography (PE/EA 7:3) affording the product (1.6 g, 4.5 mmol) in 76% yield as a colorless oil.

^1^H NMR: 7.40–7.10 (m, 5H), 4.15–3.75 (broad m, 2H), 3.46 (d, *J* = 14.0 Hz, 1H), 3.37 (d, *J* = 14.0 Hz, 1H), 3.00–2.30 (series of m, 6H), 2.18 (td, *J* = 11.8, 2.9 Hz, 1H), 1.96 (d, *J* = 13.5 Hz, 1H), 1.76 (t, *J* = 10.5 Hz, 1H), 1.44 (s, 9H), 1.08 (s, 3H), 1.02 (s, 3H).

^13^C NMR: 154.62, 138.67, 128.79, 128.33, 127.07, 79.71, 66.18, 62.84, 57.34, 53.99, 53.65, 47.50 (broad), 44.58, 44.00 (broad), 28.56, 26.61, 16.05.

HR-MS (m/z): [M+H]^+^ calcd for C_21_H_34_N_3_O_2_, 360.2646; found, 360.2628.

(S)-*tert*-butyl 6,6-dimethylhexahydro-*1H*-pyrazino[1,2-*a*]pyrazine-2(*6H*)-carboxylate **9**


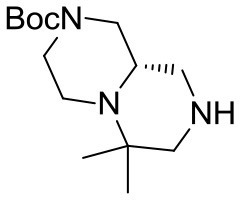


The reaction was performed in a Parr Shaker Hydrogenation apparatus. *N*-benzyl tertiary amine (3.0 g, 8.3 mmol) was dissolved im methanol (35 mL) under argon atmosphere and 10% Pd/C (0.4 g) was added. The inert atmosphere was then replaced by hydrogen gas (55 psi) and the suspension mechanically shaken for 2 days. Hydrogen atmosphere was then removed, the solution was filtered over a short celite pad and concentrated under reduced pressure. The crude material was recovered in quantitative yield and used for the final acylations without further purification.

^1^H NMR: 4.25–3.75 (broad m, 2H), 3.00–2.30 (series of m, 6H), 2.00–1.60 (m, 3H), 1.49 (s, 9H), 1.09 (s, 3H), 1.04 (s, 3H). *NH is missing*.

^13^C NMR: 154.53, 79.69, 68.49, 59.24, 55.31, 53.66, 53.23, 46.47, 44.39, 28.45, 26.63, 16.20.

HR-MS (m/z): [M+H]^+^ calcd for C_14_H_28_N_3_O_2_, 270.2176; found, 270.2178.

### Synthesis of scaffold 10

(R)-*tert*-butyl 8′-benzyl-7′,9′-dioxohexahydro-*1*′*H*-spiro[azetidine-3,6′-pyrazino[2,1-*c*][1,4]oxazine]-1-carboxylate


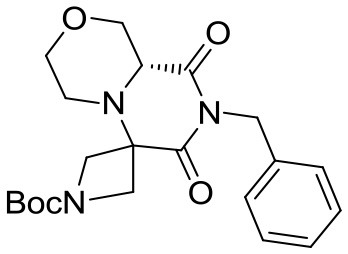


(R)-Morpholine-2-carboxylic acid hydrochloride (6.4 g, 38 mmol) was dissolved in methanol (70 mL) within a pressure flask together with *tert*-butyl-3-oxoazetidine-1-carboxylate (6.5 g, 38 mmol), benzyl isocyanide (4.6 mL, 38 mmol) and triethylamine (10.6 mL, 76 mmol). The flask was then sealed, warmed up to 65°C and left stirring for 10 days. The solution was then allowed to cool down and the solvent removed in vacuo. Crude material was diluited with ethyl acetate and washed with 5% sodium bicarbonate solution and brine. The organic layer was dried over sodium sulfate, filtered and concentrated under reduced pressure.

The crude material was purified by flash chromatography (PE/EA 7:3), yielding 11 g of product (28 mmol, 73% yield) as a yellow foam.

^1^H NMR: 7.33–7.22 (m, 5H), 5.04 (d, *J* = 13.5 Hz, 1H), 4.92 (d, *J* = 13.8 Hz, 1H), 4.57 (d, *J* = 8.8 Hz, 1H), 4.35–4.20 (m, 1H), 4.10 (d, *J* = 8.6 Hz, 1H), 3.95–3.65 (m, 5H), 3.47 (broad t, *J* = 3.5 Hz, 1H), 2.75–2.50 (m, 2H), 1.45 (s, 9H).

^13^C NMR: 170.53, 168.75, 156.04, 136.47, 128.93, 128.71, 127.88, 80.54, 66.34, 66.23, 66.15, 60.47, 55.97, 53 (broad), 44.15, 43.09, 28.42.

HR-MS (m/z): [M+H]^+^ calcd for C_21_H_28_N_3_O_5_, 402.2023; found, 402.2001.

(S)-*tert*-butyl 8′-benzylhexahydro-*1*′*H*-spiro[azetidine-3,6′-pyrazino[2,1-*c*][1,4]oxazine]-1-carboxylate


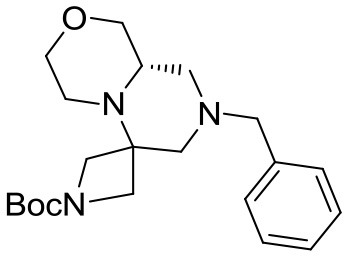


Cyclic imide (6.2 g, 15.4 mmol) was dissolved in THF (80 mL) under nitrogen atmosphere at room temperature. The solution was cooled down to 0°C and borane-dimethylsulfide complex (2 M solution in THF, 38.6 mL, 77 mmol) was slowly added in two aliquots with 1 h interval. Once the addition was complete the reaction was warmed up to 60°C and left stirring overnight.

The reaction was then cooled down to 0°C again and methanol (80 mL) was added dropwise in 1 h, then the reaction was left stirring for 1 day to allow complete decomposition of boranes. The solvents were then removed under reduced pressure and the crude material was purified by flash chromatography (PE/EA 4:6) affording the product (5.0 g, 13.4 mmol) in 87% yield as a colorless oil.

^1^H NMR: 7.35–7.20 (m, 5H), 4.12 (broad d, *J* = 9.0 Hz, 1H), 3.94 (d, *J* = 9.6 Hz, 1H),), 3.87 (broad d, *J* = 11.1 Hz, 1H), 3.70–3.10 (series of m, 7H), 2.90 (t, *J* = 9.7 Hz, 2H), 2.60–2.40 (m, 3H), 2.18 (d, *J* = 10.8 Hz, 1H), 1.80–1.70 (m, 1H), 1.42 (s, 9H).

^13^C NMR: 155.97, 137.46, 128.59, 128.16, 127.07, 79.26, 69.25, 66.93, 62.35, 61.92, 56.09, 54.79, 53.71, 53.61, 51 (broad), 44.84, 28.19.

HR-MS (m/z): [M+H]^+^ calcd for C_21_H_32_N_3_O_3_, 374.2438; found, 374.2420.

(S)-*tert*-butyl hexahydro-*1*′*H*-spiro[azetidine-3,6′-pyrazino[2,1-*c*][1,4]oxazine]-1-carboxylate **10**


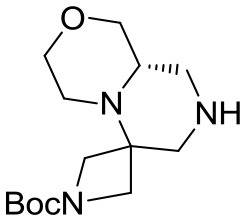


*N*-benzyl tertiary amine (4.5 g, 12 mmol) was dissolved im ethanol (80 mL) under argon atmosphere and 10% Pd/C (0.75 g) was added. The inert atmosphere was then replaced by hydrogen gas (balloon, atmospheric pressure) and the suspension left stirring at 60°C for 2 days. Hydrogen atmosphere was then removed, the solution was filtered over a short celite pad and concentrated under reduced pressure. The crude material was recovered in quantitative yield and used for the final acylations without further purification.

^1^H NMR: 4.16 (dd, *J* = 9.0, 1.5 Hz, 1H), 3.98 (d, *J* = 9.6 Hz, 1H), 3.92 (broad d, *J* = 11.4 Hz, 1H), 3.75–3.60 (m, 3H), 3.45 (d, *J* = 9.9 Hz, 1H), 3.25–3.10 (m, 2H), 2.93 (dt, *J* = 11.7, 1.8 Hz, 1H), 2.88 (dd, *J* = 14.1, 1.8 Hz, 1H), 2.80–2.68 (m, 1H), 2.61 (td, *J* = 11.4, 3.3 Hz, 1H), 2.50–2.38 (m, 2H), 1.44 (s, 9H).

^13^C NMR: 156.23, 80.25, 68.72, 68.55, 67.13, 55.02, 54 (broad), 52.63, 51 (broad), 44.22, 28.42.

HR-MS (m/z): [M+H]^+^ calcd for C_14_H_26_N_3_O_3_, 284.1969; found, 284.1960.

(6R,9aS)-*tert*-butyl 8-benzyl-6-(hydroxymethyl)-6-methylhexahydro-*1H*-pyrazino[1,2-*a*]pyrazine-2(*6H*)-carboxylate **11**


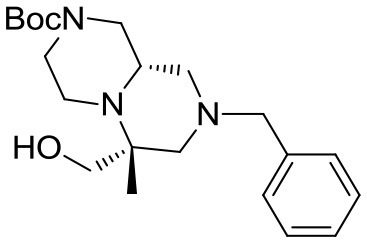


After Ugi reaction and DBU cyclization, performed as described above, the resulting cyclic imide (4.0 g, 9.9 mmol) was dissolved in THF (45 mL) under nitrogen atmosphere at room temperature. The solution was cooled down to 0°C and borane-dimethylsulfide complex (2 M solution in THF, 25.0 mL, 49.7 mmol) was slowly added in two aliquots with 1 h interval. Once the addition was complete the reaction was warmed up to 60°C and left stirring overnight.

The reaction was then cooled down to 0°C again and methanol (45 mL) was added dropwise in 1 h, then the reaction was left stirring for 1 day to allow complete decomposition of boranes. The solvents were then removed under reduced pressure and the crude material was purified by flash chromatography (PE/EA 1:1) affording the product (2.1 g, 5.6 mmol) in 57% yield as a colorless oil.

^1^H NMR: 7.40–7.10 (m, 5H), 4.10–3.70 (broad m, 2H), 3.47 (d, *J* = 13.3 Hz, 1H), 3.37 (d, *J* = 13.3 Hz, 1H), 3.15–2.60 (series of m, 6H), 2.47 (broad t, *J* = 6.4 Hz, 1H), 2.43 (dd, *J* = 11.2, 1.8 Hz, 1H), 2.34 (d, *J* = 11.2, Hz, 1H), 2.23 (td, *J* = 11.7, 2.9 Hz, 1H), 1.73 (t, *J* = 11.3 Hz, 1H + –OH overlapped, 1H), 1.45 (s, 9H), 1.02 (s, 3H).

^13^C NMR: 154.56, 138.38, 128.86, 128.40, 127.21, 79.97, 64.95, 62.93, 61.61, 57.51, 56.79, 53.76, 47.50 (broad), 45.00 (broad), 44.49, 28.54, 14.06.

HR-MS (m/z): [M+H]^+^ calcd for C_21_H_34_N_3_O_3_, 376.2595; found, 376.2578.

### General conditions for introduction of the first diversomer

#### Acylation reaction

##### Method A

Compound **4**, **9** or **10** (1.0 mmol) was dissolved in dry DCM (5 mL) under nitrogen and the carboxylic acid (1.1 mmol) was added. The solution was then cooled to 0°C and TBTU (1.1 mmol) and DIPEA (1.5 mmol) were added. The reaction was allowed to warm up to room temperature and left stirring under nitrogen overnight. The solution was then diluted with aqueous ammonium chloride and extracted twice with ethyl acetate. The organic phase was anhydrified over sodium sulfate, concentrated and purified by flash chromatography.

##### Method B

Compound **4**, **9** or **10** (1.0 mmol) was dissolved in dry DCM (5 mL) under nitrogen and the acyl (or sulfonyl) chloride (1.0 mmol) and triethylamine (1.5 mmol) were added. The reaction was left stirring under nitrogen overnight. The solution was then diluted with aqueous sodium carbonate and extracted twice with ethyl acetate. The organic phase was anhydrified over sodium sulfate, concentrated and purified by flash chromatography.

##### Method C

Compound **9** or **10** (1.0 mmol) was dissolved in dry DCM (5 mL) under nitrogen and the isocyanate (1.0 mmol) and triethylamine (1.5 mmol) were added. The reaction was left stirring under nitrogen overnight. The solution was then diluted with aqueous sodium bicarbonate and extracted twice with DCM. The organic phase was dired over sodium sulfate, concentrated and purified by flash chromatography.

#### Reductive amination reaction

Compound **4** (1.0 mmol) was dissolved in MeOH and the aldehyde/ketone (1.2 mmol) and Borane-Pyridine complex (2 mmol) were added followed by one drop od 0.5 M HCl. The reaction was left stirring overnight. The solution was then poured over an SCX-cartridge and eluted with MeOH until no impurity could be detected anymore. Then the cartridge was eluted with 5 mL ammonia in MeOH (2–3 M) and the resulting solution was evaporated to dryness. The remaining residue was purified by flash chromatography.

### General conditions for the introduction of the second diversomer

#### Boc cleavage

The Boc-protected compound was dissolved in HCl/MeOH (1.5 M) and the mixture was stirred at rt until completion (usually 1–2 h). The solvent was evaporated, the remainder was dried and used for the next steps withiut further purification.

#### Acylation reaction

##### Acid chlorides

To a solution of the starting material (usually 50–80 mg) in CH_2_Cl_2_ were added 500 mg Poly(4-vinylpyridine) and the acid chloride. The mixture was agitated at rt for 16 h. After completion (as indicated by TLC-monitoring) the mixture was filtrated. To the filtrate was added half saturated NaHCO_3_ solution, the mixture was agitated for 30 min, then the phases were separated and the aqueous phase was extracted once again with CH_2_Cl_2_. The combined organic phases were dried over Na_2_SO_4_ and the solvent was evaporated.

##### Isocyanates

To a solution of the starting material (usually 50–80 mg) in CH_2_Cl_2_ were added 500 mg Poly(4-vinylpyridine) and the isocyanate (1.05 equiv.) The mixture was agitated at rt for 16 h. After completion (as indicated by TLC-monitoring) the mixture was filtrated and the solvent was evaporated.

##### Sulfonyl chlorides

To a solution of the starting material (usually 50–80 mg) in CH_2_Cl_2_/Py (3/7, v:v) was added the sulfonyl chloride (5 equiv.) at 0°C. The mixture was agitated at rt for 16 h. After completion (as indicated by TLC-monitoring) the solvent was evaporated. The residue was redissolved in CH_2_Cl_2_ and extracted with saturated NaHCO_3_ solution. The organic phase was dried over Na_2_SO_4_ and the solvent was evaporated.

#### Reductive amination reaction

To a solution of the free amine *19* in 2 ml dry MeOH were added 2 equivalents of the aldehyde or ketone, 2.5 equivalents of the borane-pyridine complex, 1 drop 0.5 M HCl and stirring was continued at rt overnight. LC/MS-check! After completion of the reaction the solution was poured over a SCX-cartridge (Phenomenex, Strata SCX, 0,6 mmol/g) and eluted with methanol until no impurity could be detected anymore. Then the cartridge was eluted with ca. 8 M methanolic ammonia solution. After removal of the solvent in a turbovap the remaining residue was finally evaporated to dryness in a Rotary Vacuum Concentrator (Christ, Germany; 40°C, 6 h) and subjected to analysis.

### Analytical data for selected library members

#### Compound 6{*3,4*}


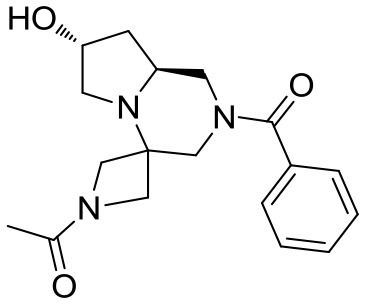


^1^H NMR (CDCl_3_, 70°C): 7.47–7.33 (m, 5H), 4.74–4.27 (m, 2H), 4.26–3.90 (m, 3H), 3.87–3.56 (m, 2H), 3.55–3.43 (m, 1H), 2.95 (d, *J* = 12.9 Hz, 1H), 2.79–2.4 (m, 3H), 2.40–2.05 (broad m, 1H), 1.85 (s, 3H), 1.81–1.56 (m, 2H).

^13^C NMR (CDCl_3_, 70°C): 171.11, 170.99, 135.72, 130.20, 128.82, 127.29, 69.04, 56.35, 55.79, 55.51, 55.26, 53.93, 52.20, 49.86, 39.18, 19.02.

HR-MS (m/z): [M+H]^+^ calcd for C_18_H_24_N_3_O_3_, 330.1812; found, 330.1836.

#### Compound 6{*4,5*}


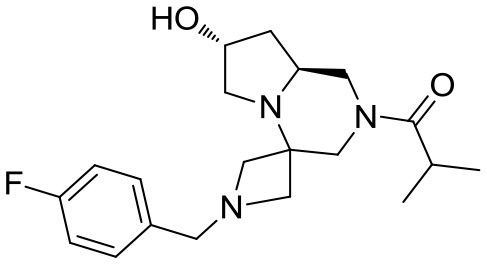


^1^H NMR: 7.30–7.17 (m, 2H), 7.05–6.90 (m, 2H), 4.92 (d, *J* = 12.9 Hz, 0.4H), 4.63 (d, *J* = 12.6 Hz, 0.6H), 4.47 (broad s, 1H), 4.23 (d, *J* = 12.7 Hz, 0.6H), 3.87 (d, *J* = 12.1 Hz, 0.4H), 3.60 (s, 2H), 3.57–3.36 (m, 1H), 3.27–2.84 (m, 5H), 2.84–2.15 (m, 5H), 1.86–1.63 (m, 2H), 1.21–1.04 (m, 6H).

The spectrum was not recorded at 70°C because azetidine Hs collapsed.

^13^C NMR (CDCl_3_, 70°C): 176.20, 162.33 (d, *J* = 245 Hz), 134.25, 129.98 (d, *J* = 7.8 Hz), 115.25 (d, *J* = 21.2 Hz), 69.46, 62.21, 60.08, 56.74, 56.28, 55.33, 54.48, 39.54, 30.34, 19.86, 19.51.

CH_2_S of azetidine ring could only be detected in the HSQC analysis at 53 and 45 ppm.

HR-MS (m/z): [M+H]^+^ calcd for C_20_H_29_FN_3_O_2_, 362.2238; found, 362.2246.

#### Compound 13{*8,1*}


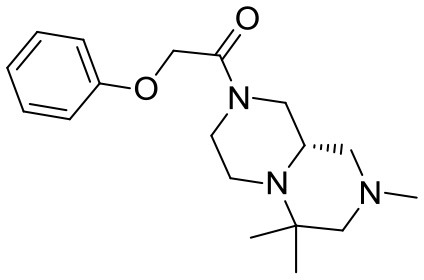


^1^H NMR (DMSO-*d*_6_, 90°C): 7.32–7.23 (m, 2H), 6.98–6.91 (m, 3H), 4.76 (s, 2H), 4.25–3.80 (m, 2H), 2.90 (broad d, *J* = 11.4 Hz, 2H), 2.77 (broad d, *J* = 10.5 Hz, 2H), 2.56–2.48 (m, 2H), 2.20 (s, 3H), 2.17–2.05 (m, 1H), 1.96 (broad d, *J* = 10.8 Hz, 1H), 1.74 (broad t, *J* = 9.6 Hz, 1H), 1.04 (s, 3H), 1.03 (s, 3H).

^13^C NMR (DMSO-*d*_6_, 90°C): 165.20, 157.72, 128.82, 120.56, 114.44, 66.73, 66.11, 57.54, 53.03, 52.62, 45.06, 43.75, 25.64, 20.34, 15.27 (one peak is missing).

HR-MS (m/z): [M+H]^+^ calcd for C_18_H_28_N_3_O_2_, 318.2176; found, 318.2164.

#### Compound 13{*6,2*}


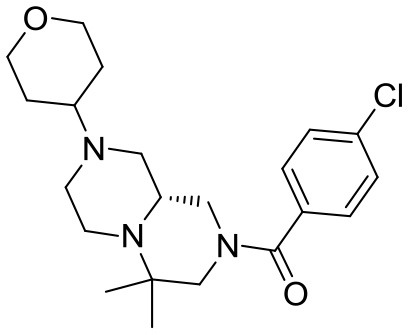


^1^H NMR (CDCl_3_, 70°C): 7.36 (d, *J* = 8.7, 2H), 7.32 (d, *J* = 8.7, 2H), 3.98 (dm, *J* = 10.5 Hz, 2H), 3.98–3.50 (very broad m, 1H), 3.33 (td, *J* = 11.7, 2.1 Hz, 2H), 2.95–2.20 (series of m, 9H), 1.88 (t, *J* = 9.5, 1H), 1.75–1.65 (m, 2H), 1.53 (qd, *J* = 12.1, 4.9, 3H), 1.06 (s, 3H), 0.98 (s, 3H).

^13^C NMR (CDCl_3_, 70°C): 169.46, 135.96, 134.65, 128.93, 128.82, 67.49 (2C), 61.03, 54.13, 53.30, 53.09, 49.73, 44.89, 29.93, 29.82, 25.98, 14.45.

HR-MS (m/z): [M+H]^+^ calcd for C_21_H_31_ClN_3_O_2_, 392.2099; found, 392.2094.

#### Compound 15{*6,3*}


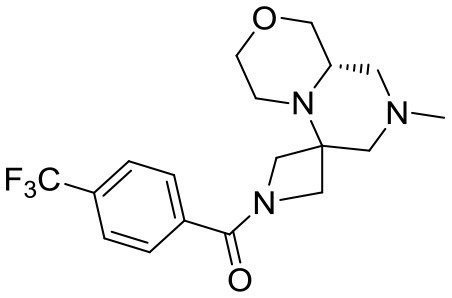


^1^H NMR: 7.76 (d, *J* = 8.2 Hz, 2H), 7.66 (d, *J* = 8.2 Hz, 2H), 4.41 (d, *J* = 9.7 Hz, 1H), 4.26 (d, *J* = 10.8 Hz, 1H), 3.97 (d, *J* = 9.7 Hz, 1H), 3.90 (broad d, *J* = 11.2 Hz, 1H), 3.73 (d, *J* = 10.8 Hz, 1H), 3.71–3.58 (m, 2H), 3.21 (dd, *J* = 11.1, 9.6, Hz, 2H), 3.00–2.87 (m, 2H), 2.62–2.44 (m, 3H), 2.24 (s, 3H), 2.24–2.19 (m, 1H), 1.75 (t, *J* = 10.9 Hz, 1H).

^13^C NMR: 169.01, 137.01, 133.21 (q, *J* = 32.8 Hz), 128.50, 125.58 (q, *J* = 3.8 Hz), 123.94 (q, *J* = 272.5 Hz), 69.76, 67.40, 64.54, 57.21, 56.16, 55.22, 55.14 (very broad), 54.06 (very broad), 46.13, 45.22.

HR-MS (m/z): [M+H]^+^ calcd for C_18_H_23_F_3_N_3_O_2_, 370.1737; found, 370.1746.

#### Compound 15{*8,4*}


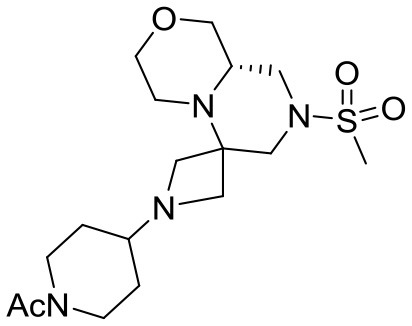


^1^H NMR (CDCl_3_, 70°C): 4.05–3.85 (m, 3H), 3.73 (dd, *J* = 11.1, 3.0 Hz, 1H), 3.63 (td, *J* = 11.0, 2.5 Hz, 2H), 3.42 (dt, *J* = 10.8, 2.6 Hz, 1H), 3.35–3.05 (m, 6H), 3.00–2.90 (m, 1H), 2.89 (d, *J* = 8.1 Hz, 1H), 2.80 (d, *J* = 12 Hz, 1H), 2.76 (s, 3H), 2.67–2.55 (m, 2H), 2.48 (t, *J* = 10.8 Hz, 1H), 2.35 (tt, *J* = 7.7, 3.7 Hz, 1H), 2.04 (s, 3H), 1.70–1.55 (m, 2H), 1.40–1.20 (m, 2H).

^13^C NMR (CDCl_3_, 70°C): 168.88, 69.14, 67.55, 62.36, 56.57 (broad), 56.43, 54.04 (broad), 53.92, 53.72, 46.15, 45.36, 44.03 (broad), 39.17 (broad), 34.99, 29.51 (broad), 28.66 (broad), 21.32.

HR-MS (m/z): [M+H]^+^ calcd for C_17_H_31_N_4_O_4_S, 387.2061; found, 387.2052.

## Author contributions

All authors listed have made a substantial, direct and intellectual contribution to the work, and approved it for publication.

### Conflict of interest statement

JS and CM are employed by AnalytiCon Discovery GmbH.

The remaining authors declare that the research was conducted in the absence of any commercial or financial relationships that could be construed as a potential conflict of interest.
