# Corrigendum: Exploitation of the Ugi 5-Center-4-Component Reaction (U-5C-4CR) for the Generation of Diverse Libraries of Polycyclic (Spiro)Compounds

**DOI:** 10.3389/fchem.2018.00510

**Published:** 2018-10-19

**Authors:** Lisa Moni, Fabio De Moliner, Silvia Garbarino, Jörn Saupe, Christian Mang, Andrea Basso

**Affiliations:** ^1^Dipartimento di Chimica e Chimica Industriale, Università degli Studi di Genova, Genova, Italy; ^2^AnalytiCon Discovery GmbH, Potsdam, Germany

**Keywords:** multicomponent reactions, diversity oriented synthesis, scaffold diversity, combinatorial libraries, isocyanides

In the original article, there was an error. The sentence “The contemporary presence of a secondary amino group and a cyclic ketone kinetically disfavored the Ugi reaction, which did not proceed at room temperature, even with the addition of a Lewis acid as previously reported by Dawidowski” was misleading.

A correction has been made to Results and Discussion, Second paragraph

The contemporary presence of a secondary amino group and a cyclic ketone kinetically disfavored the Ugi reaction, which did not proceed at room temperature, even with the addition of a Lewis acid as previously reported by Dawidowski for other substrates (Dawidowski et al., 2014). However, by performing it in a sealed tube at 65°C, a mixture of Ugi adduct **1** and cyclic imide **2** was isolated after 6 days. This mixture was subjected to complete cyclization by addition of a catalytic amount of DBU in acetonitrile. The overall yield after the two steps was an acceptable 47% and remarkably, under these conditions, no epimerization was observed at the L-proline α-carbon, thus allowing us to obtain compound **2** in enantiomerically and diastereomerically pure form.

The authors apologize for this error and state that this does not change the scientific conclusions of the article in any way.

The original article has been updated.

## Conflict of interest statement

The authors declare that the research was conducted in the absence of any commercial or financial relationships that could be construed as a potential conflict of interest.
