# Supplementary material for: Exploitation of the Ugi 5-Center-4-Component Reaction (U-5C-4CR) for the Generation of Diverse Libraries of Polycyclic (Spiro)Compounds
Source: Front Chem. 2018 Sep 6;6:369. doi: 10.3389/fchem.2018.00369 (PMC6136273; doi:10.3389/fchem.2018.00369)

Supplementary Material

Exploitation of the Ugi 5-centre-4-component reaction (U-5C-4CR) for the generation of diverse libraries of polycyclic (spiro)compounds

Lisa Moni[1], Fabio De Moliner[1], Silvia Garbarino[1], Jörn Saupe[2], Christian Mang[2]* and Andrea Basso[1]*

*** Correspondence:**

Christian Mang: c.mang@ac-discovery.com

Andrea Basso: andrea.basso@unige.it

^{1}H-NMR and ^{13}C-MR spectra

Compound 2

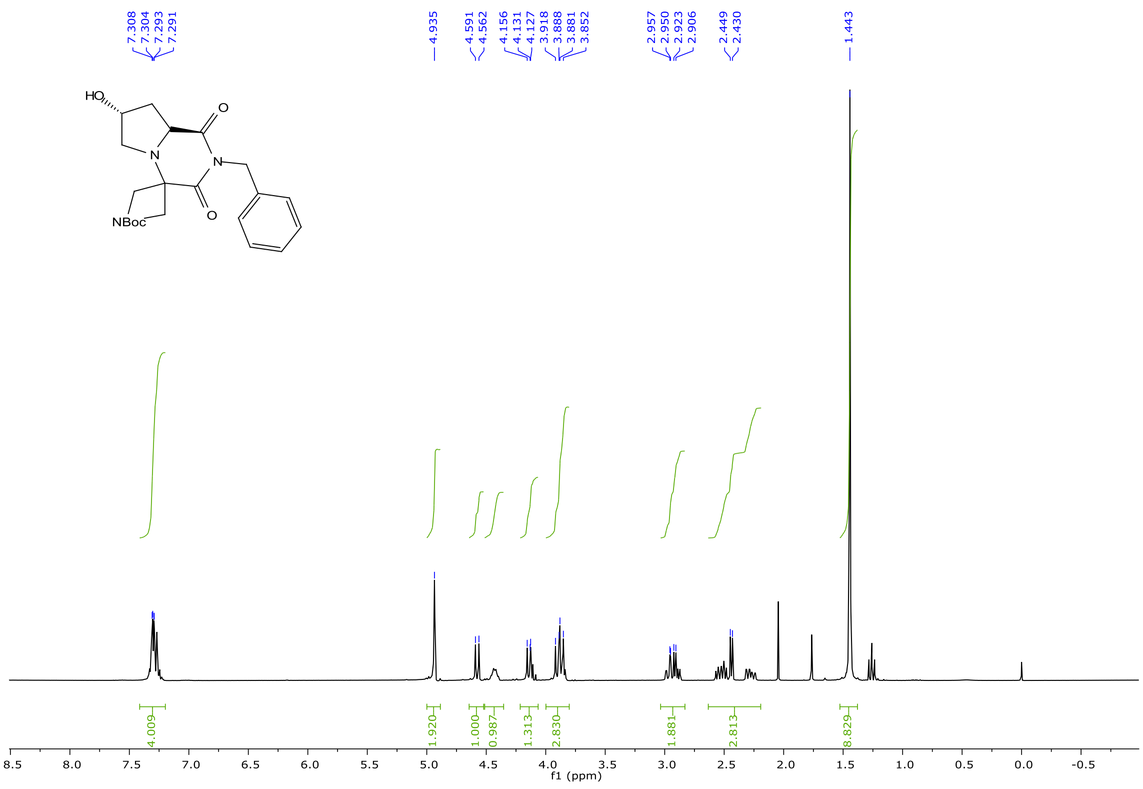
7.308
7.304
7.293
7.291
4.935
4.591
4.562
4.156
4.131
4.127
3.918
3.888
3.881
3.852
2.957
2.950
2.923
2.906
2.449
2.430
1.443
HO
O
N
N
O
NBoc
4.009
1.920
1.000
0.987
1.313
2.830
1.881
2.813
8.829
f1 (ppm)

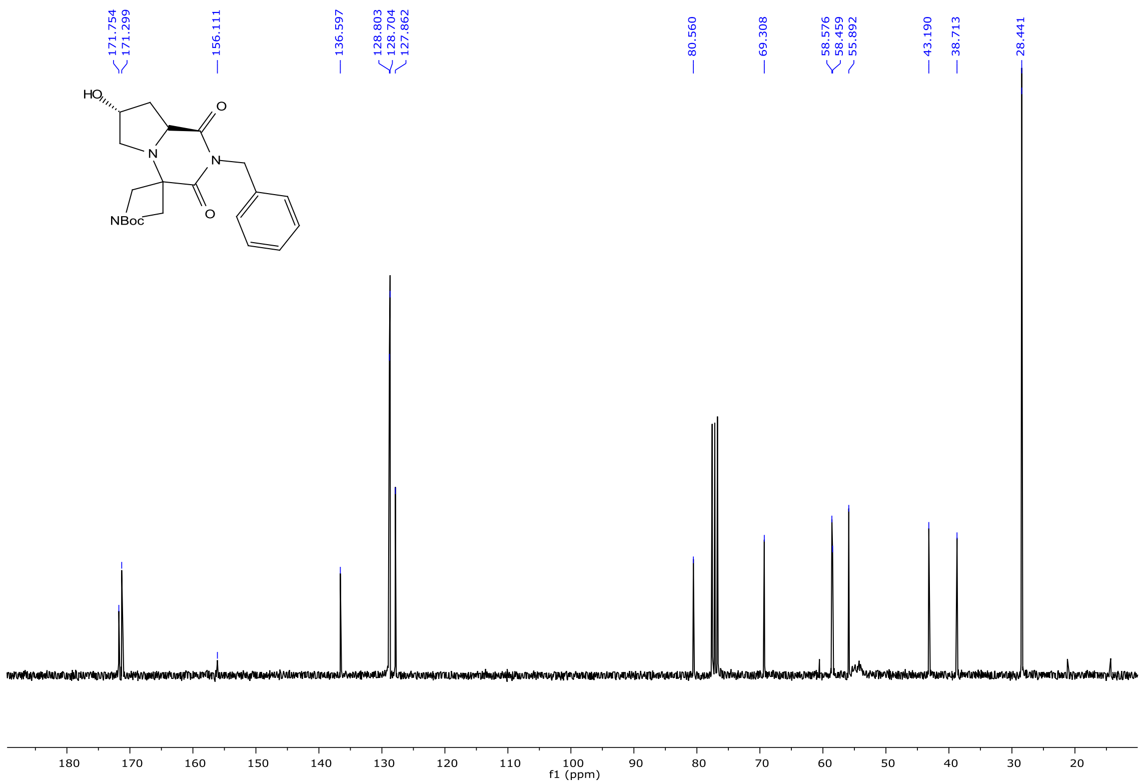
171.754
171.299
156.111
136.597
128.803
128.704
127.862
80.560
69.308
58.576
58.459
55.892
43.190
38.713
28.441
HO
O
N
N
O
NBoc
f1 (ppm)

Compound 3

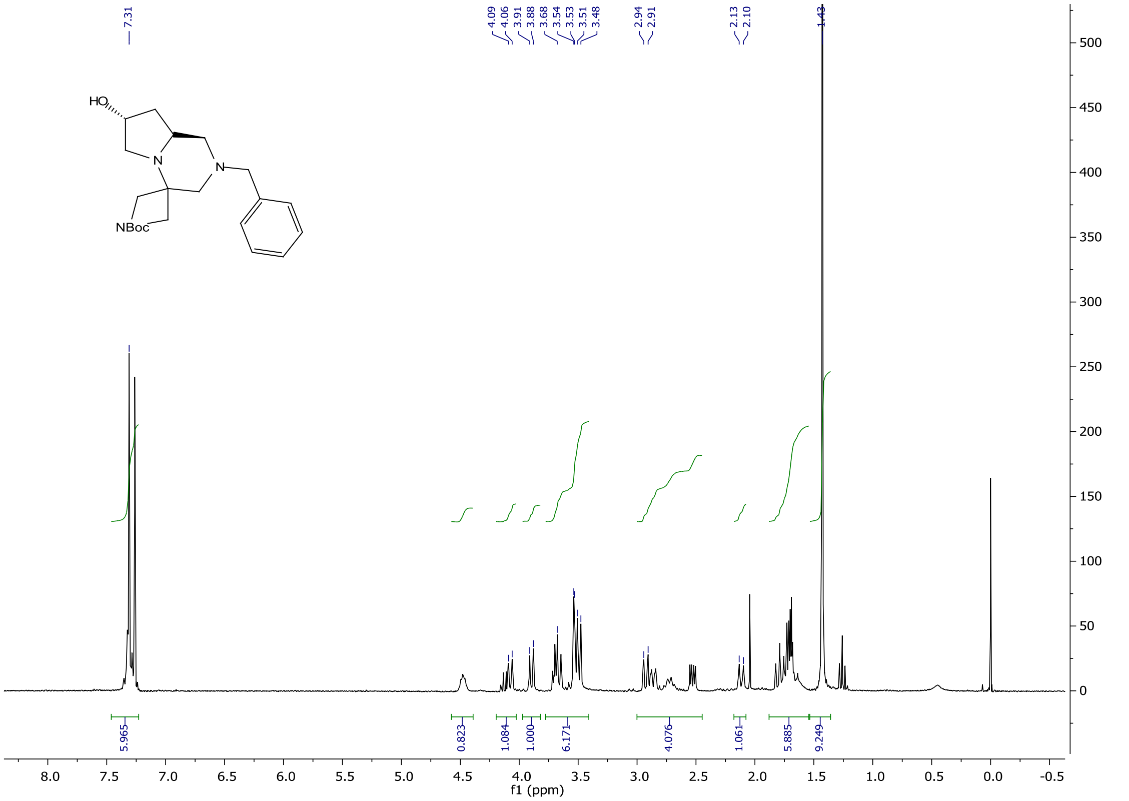

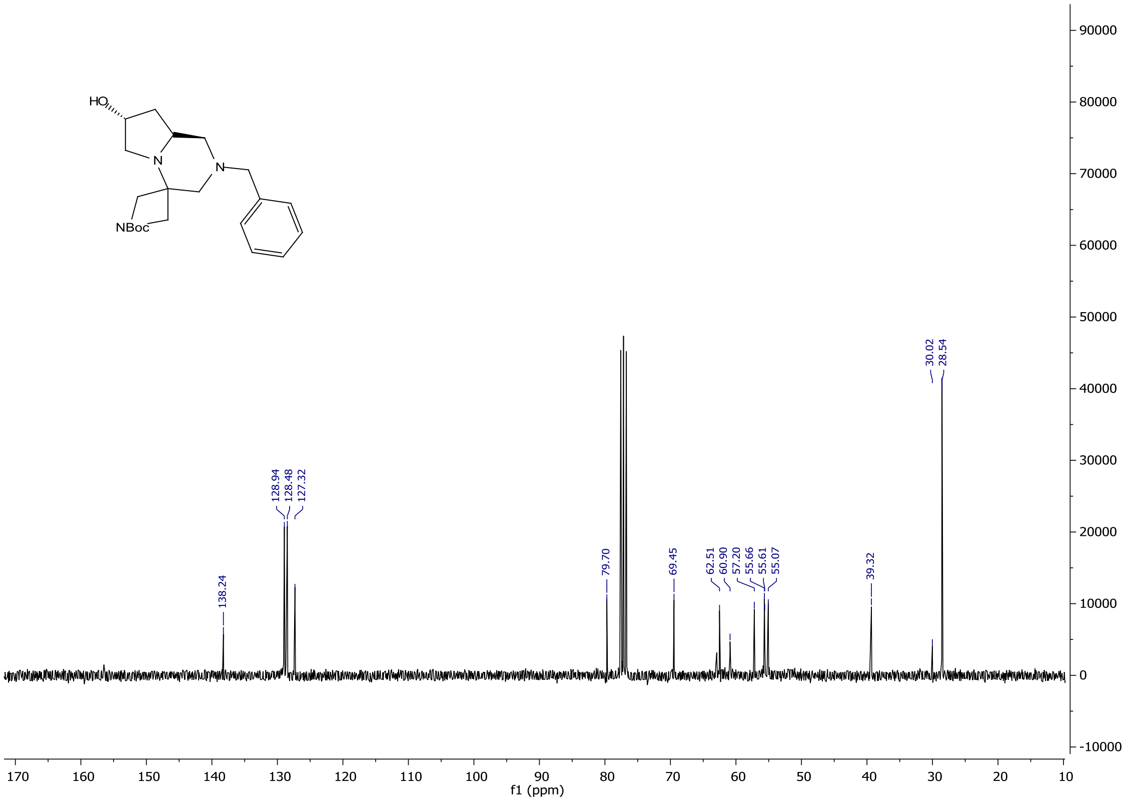

Compond 4

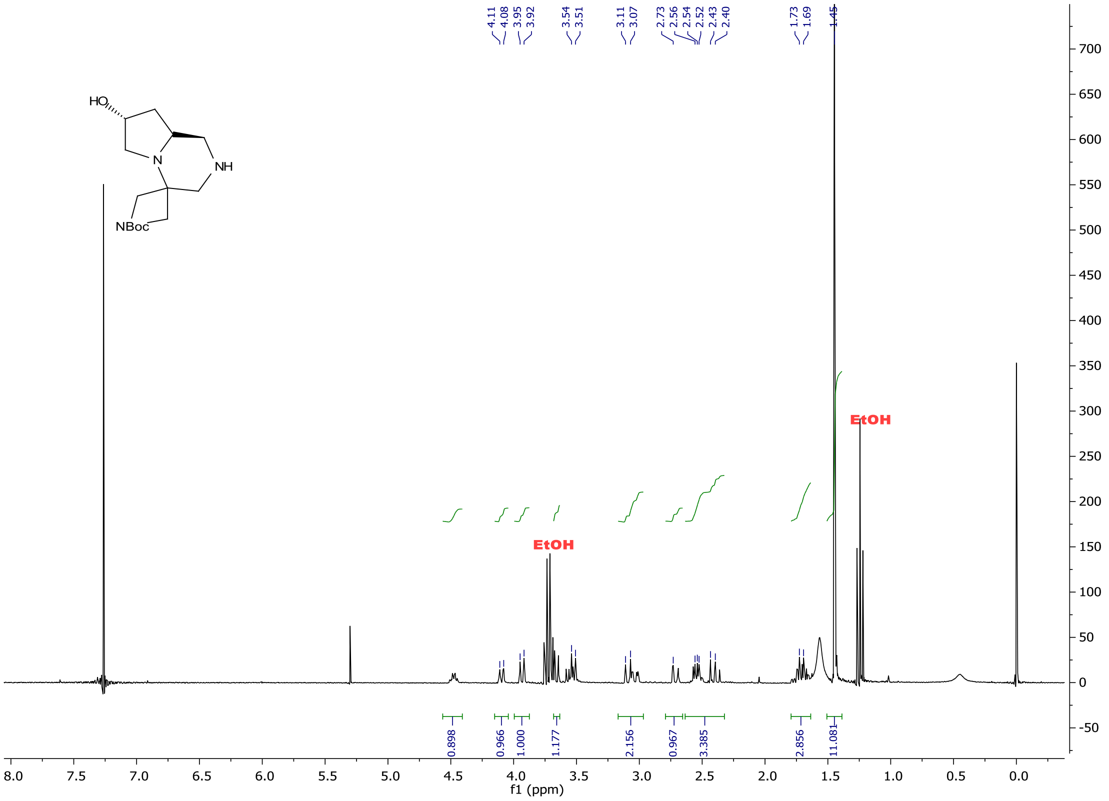
HO
N
NH
NBoc
4.11
4.08
3.95
3.92
3.54
3.51
3.11
3.07
2.73
2.56
2.54
2.52
2.43
2.40
1.73
1.69
1.45
EtOH
EtOH
0.898
0.966
1.000
1.177
2.156
0.967
3.385
2.856
11.081
f1 (ppm)

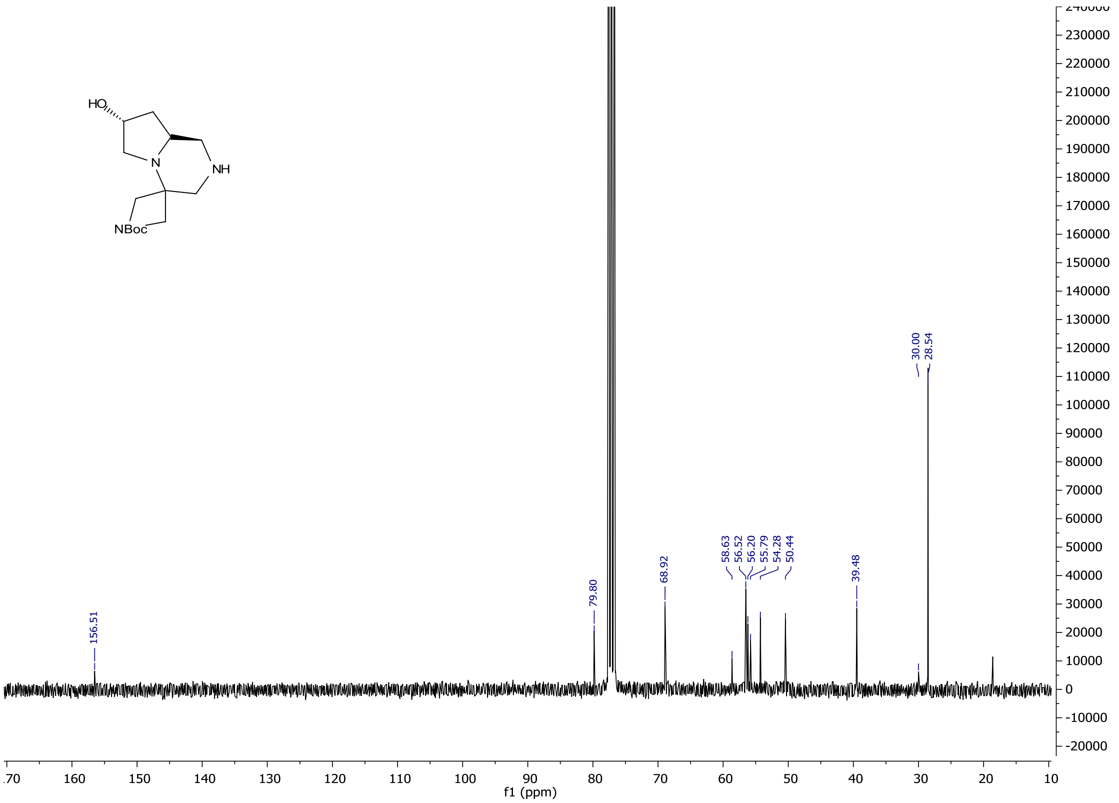
HO
N
NH
NBoc
156.51
79.80
68.92
58.63
56.52
56.20
55.79
54.28
50.44
39.48
30.00
28.54
f1 (ppm)

Intermediate in the synthesis of compound 9

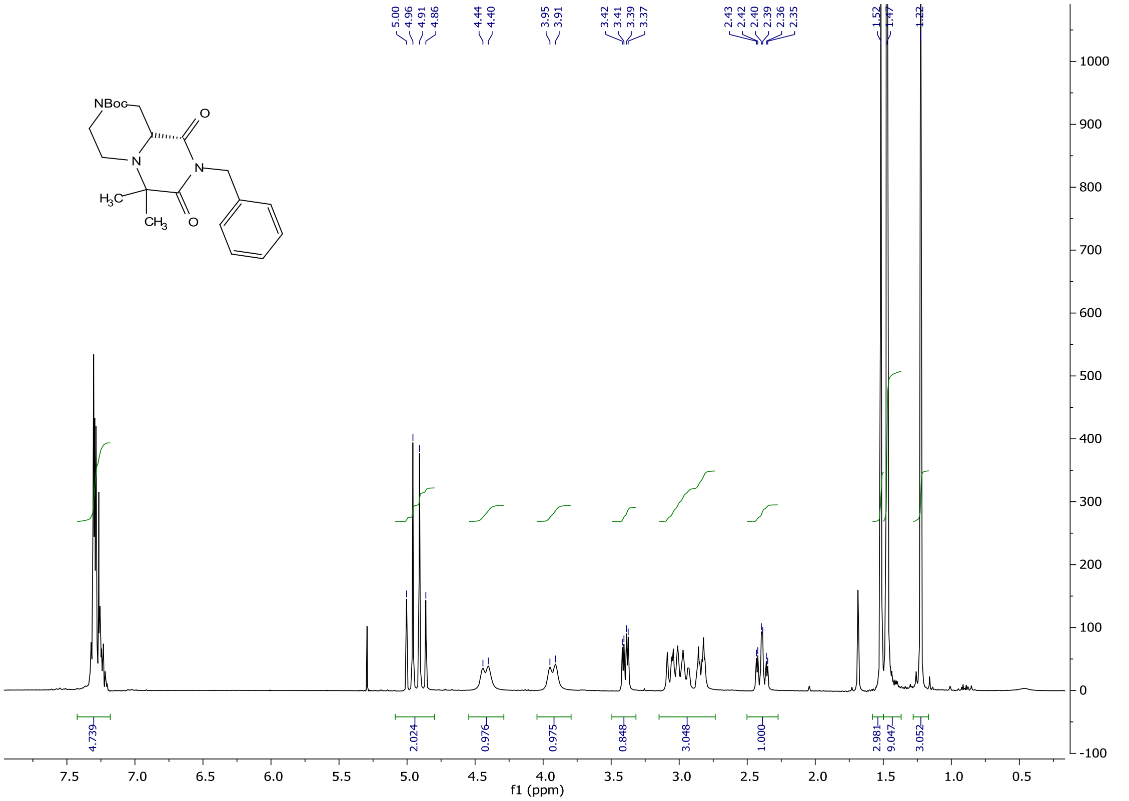

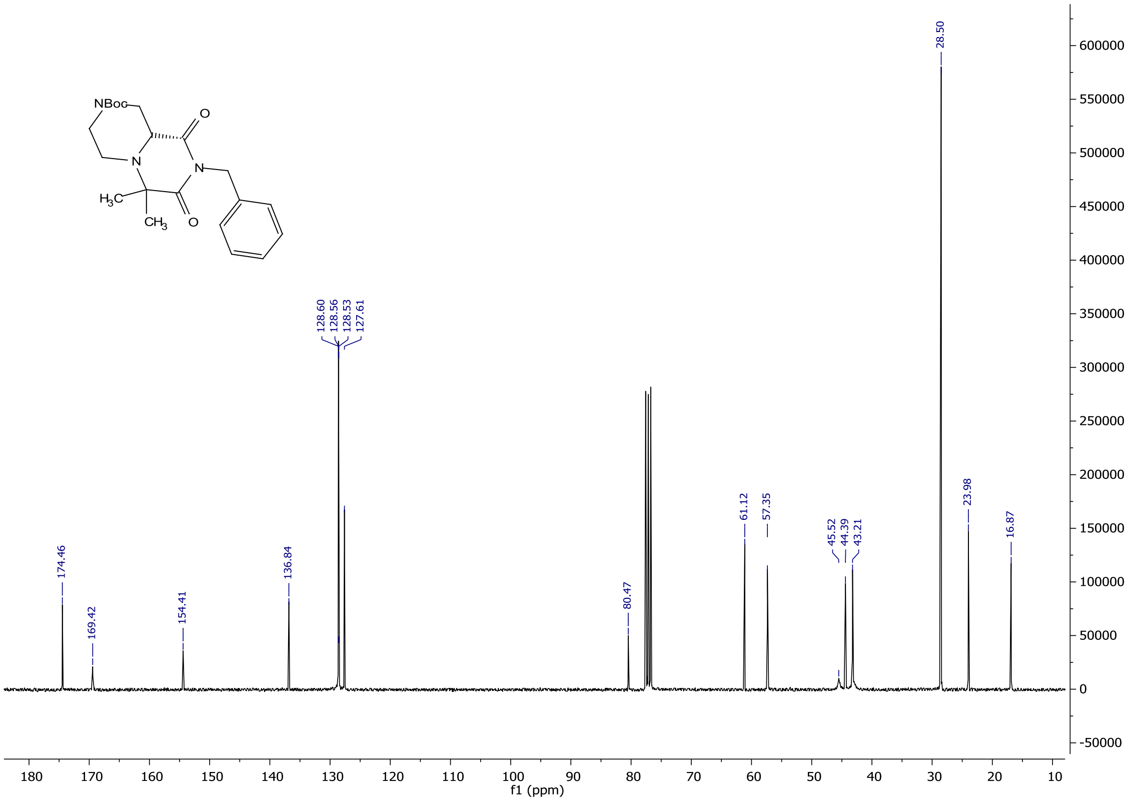

Intermediate in the synthesis of compound 9

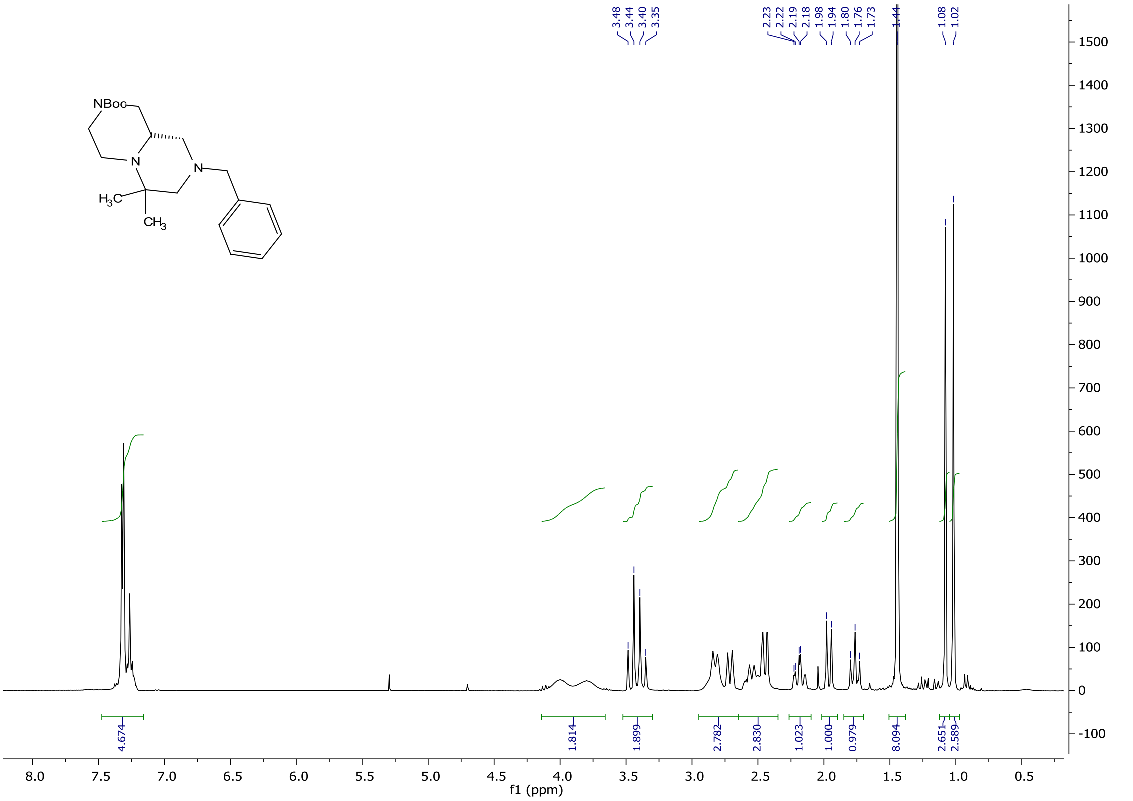

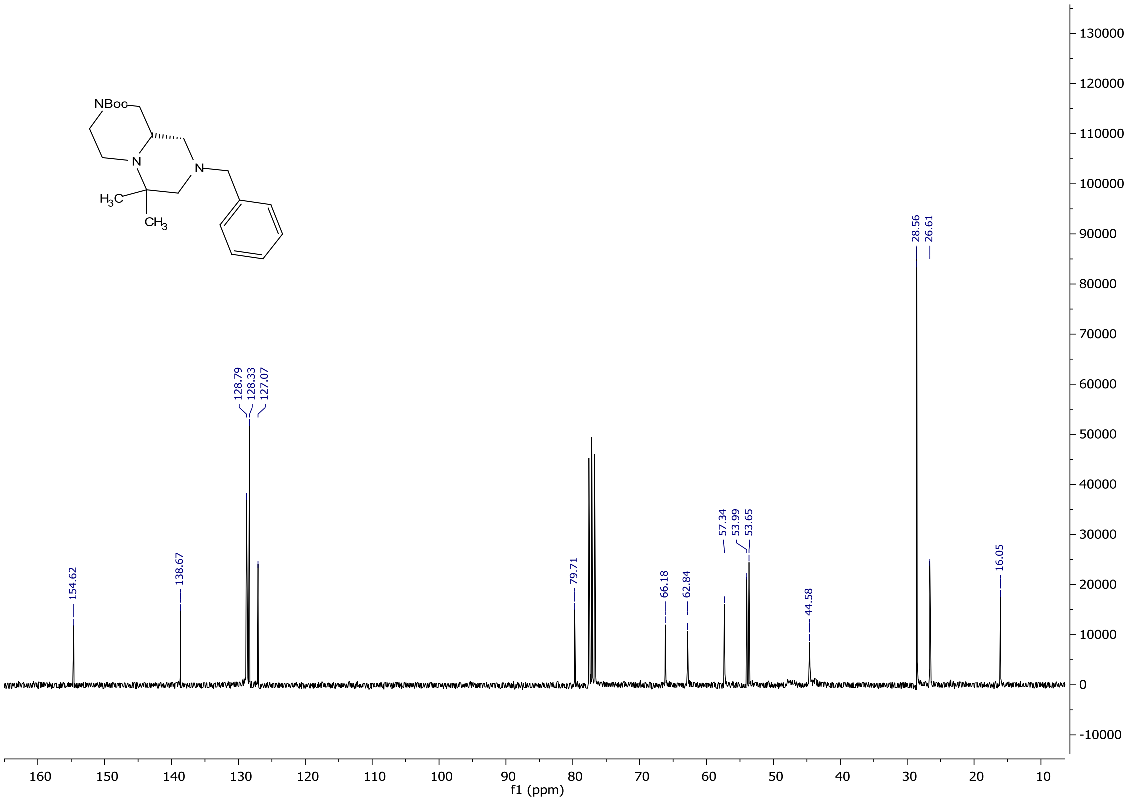

Compound 9

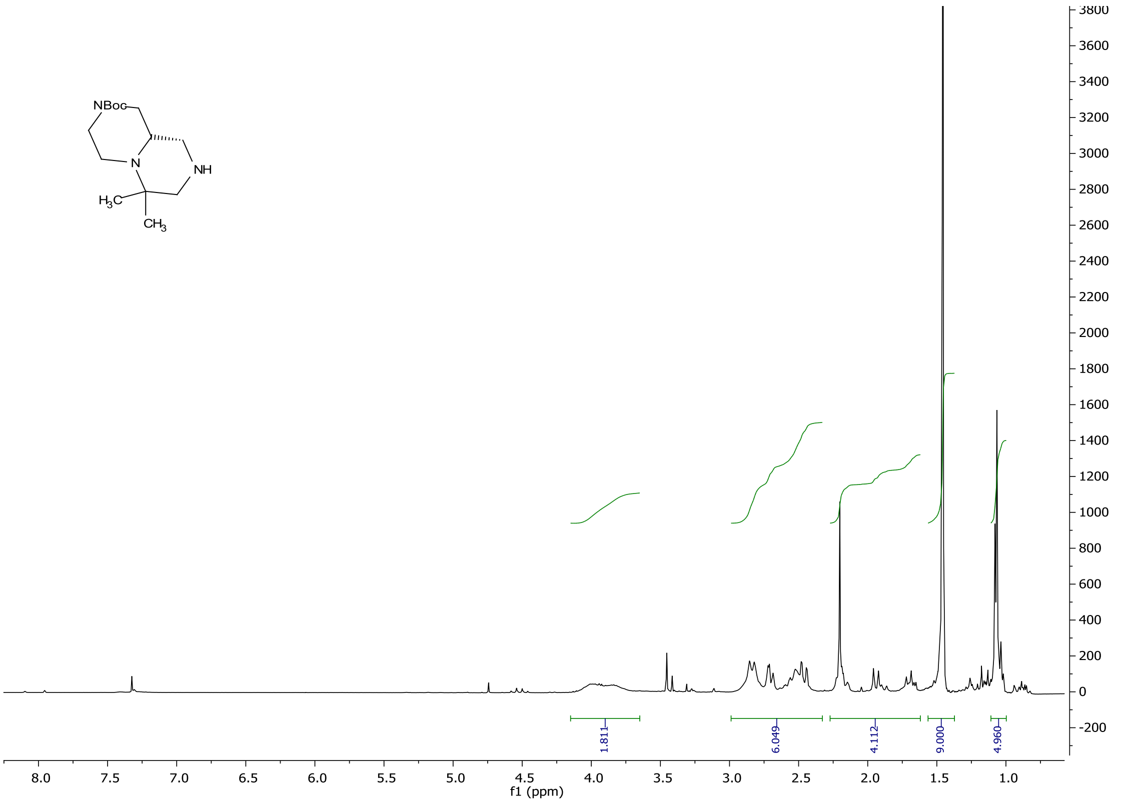

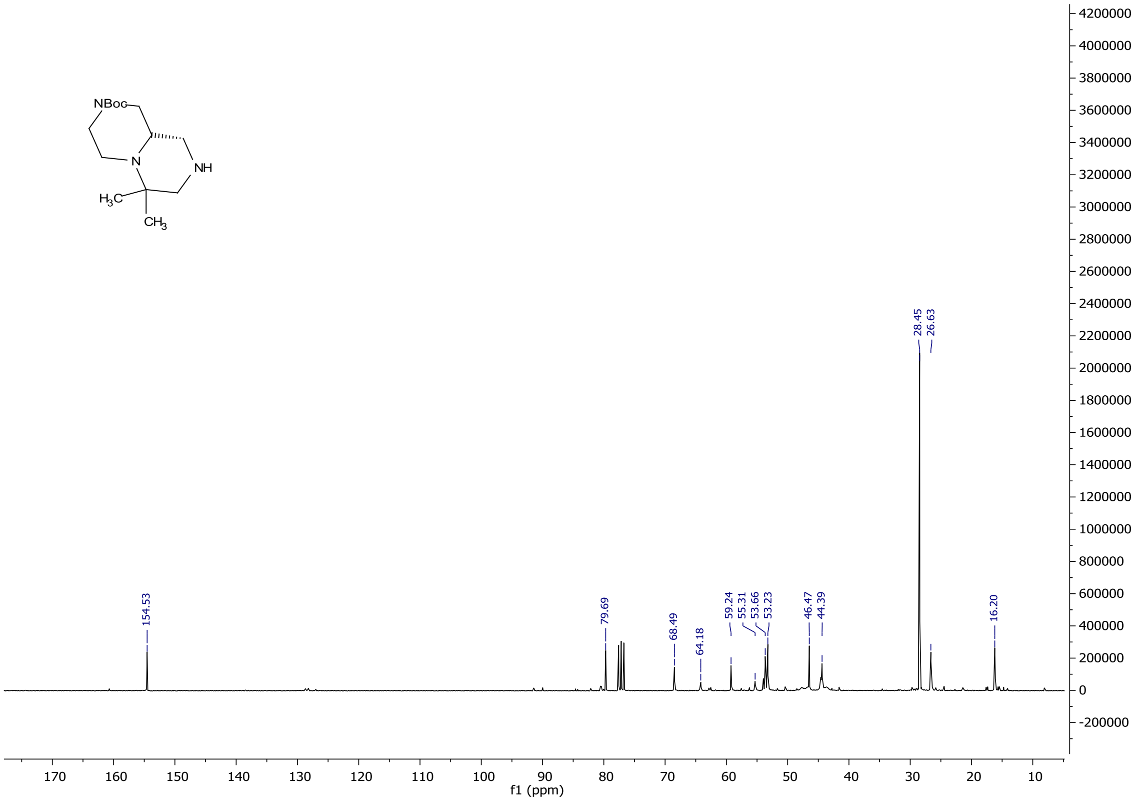

Intermediate in the synthesis of compound 10

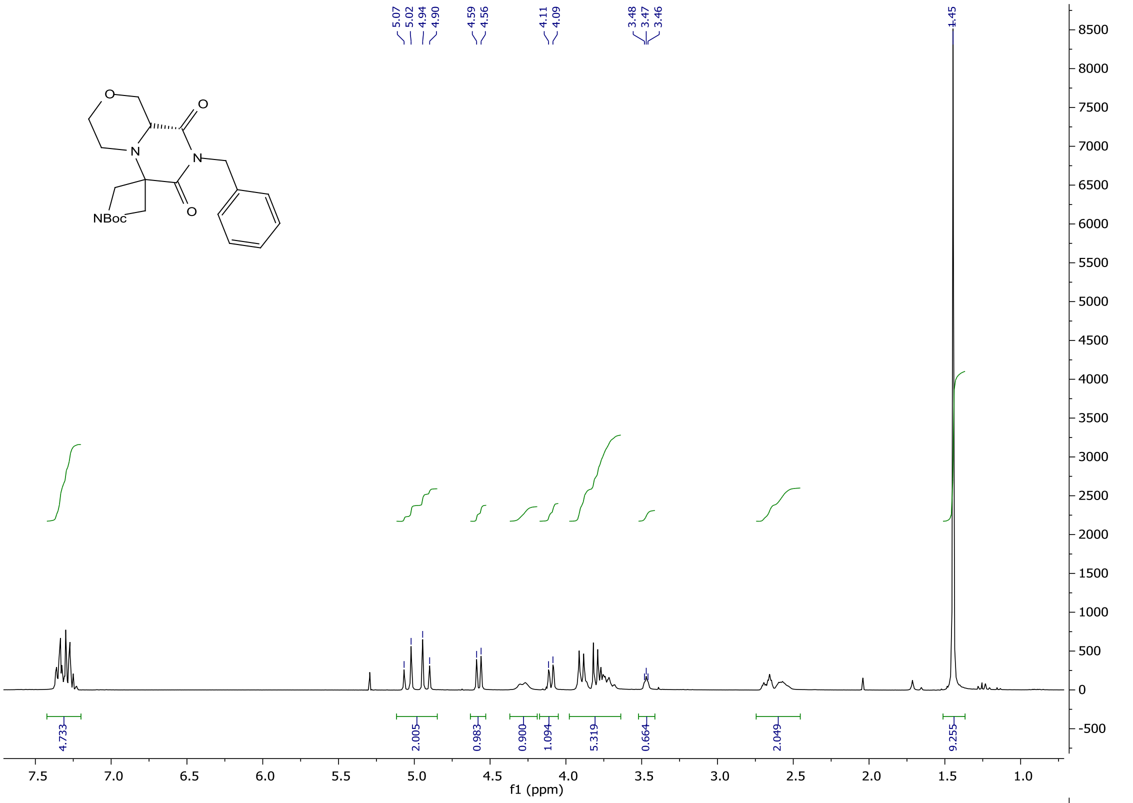

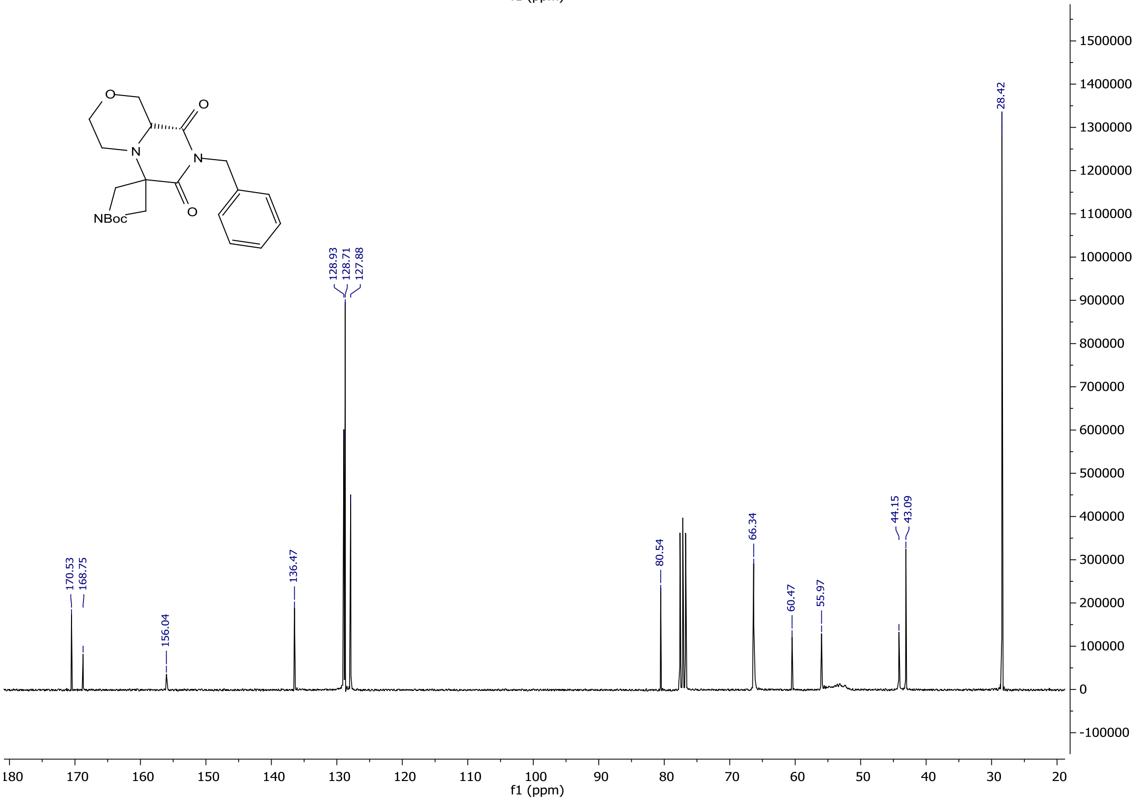

Intermediate in the synthesis of compound 10

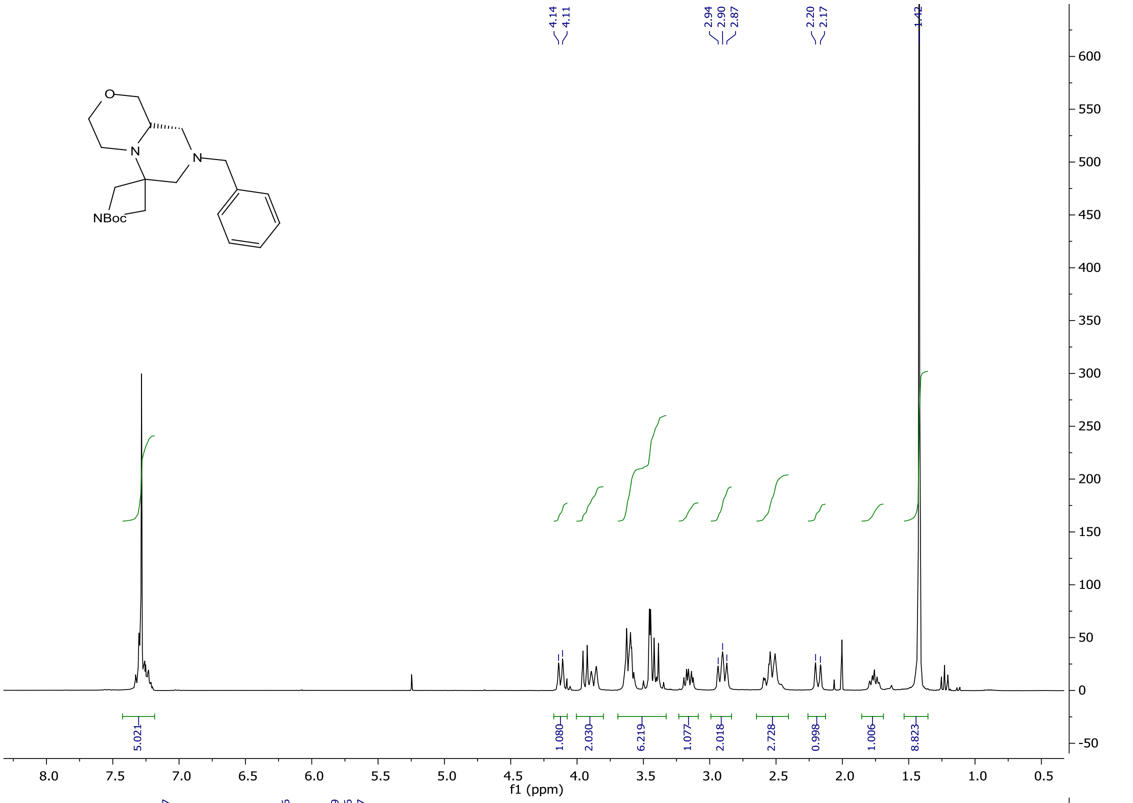

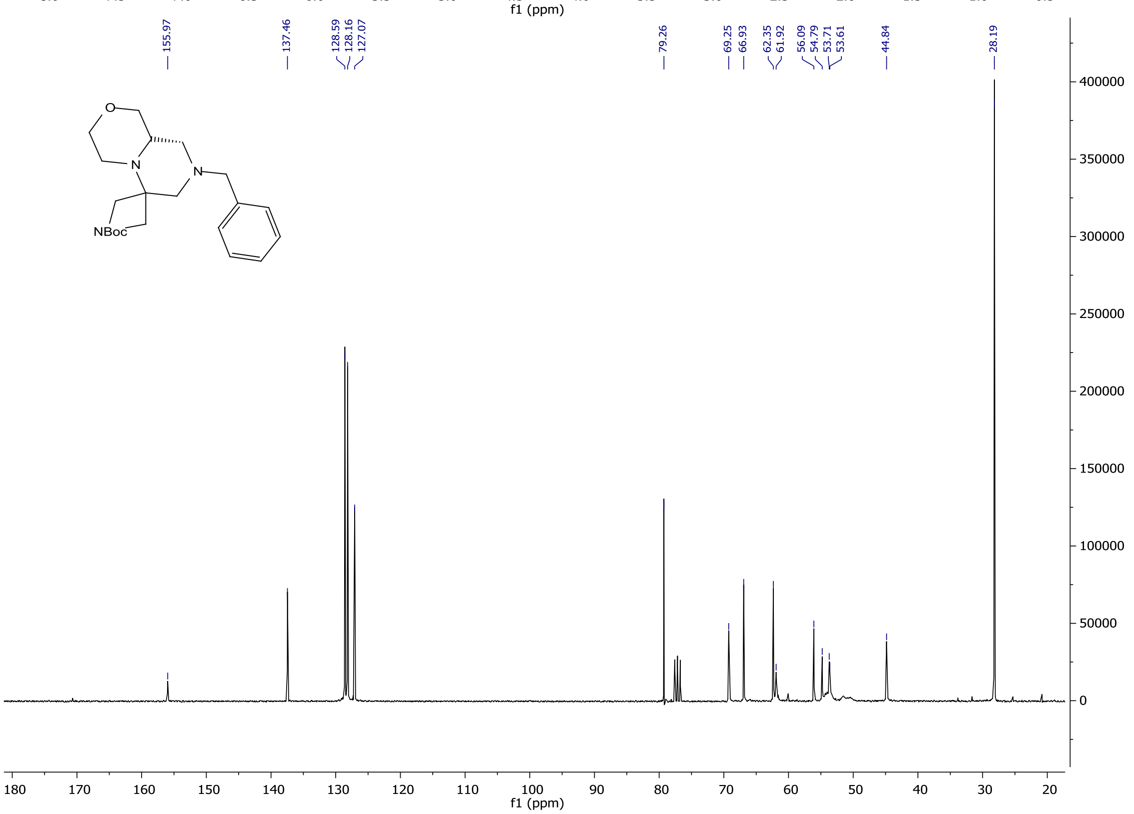

Compound 10

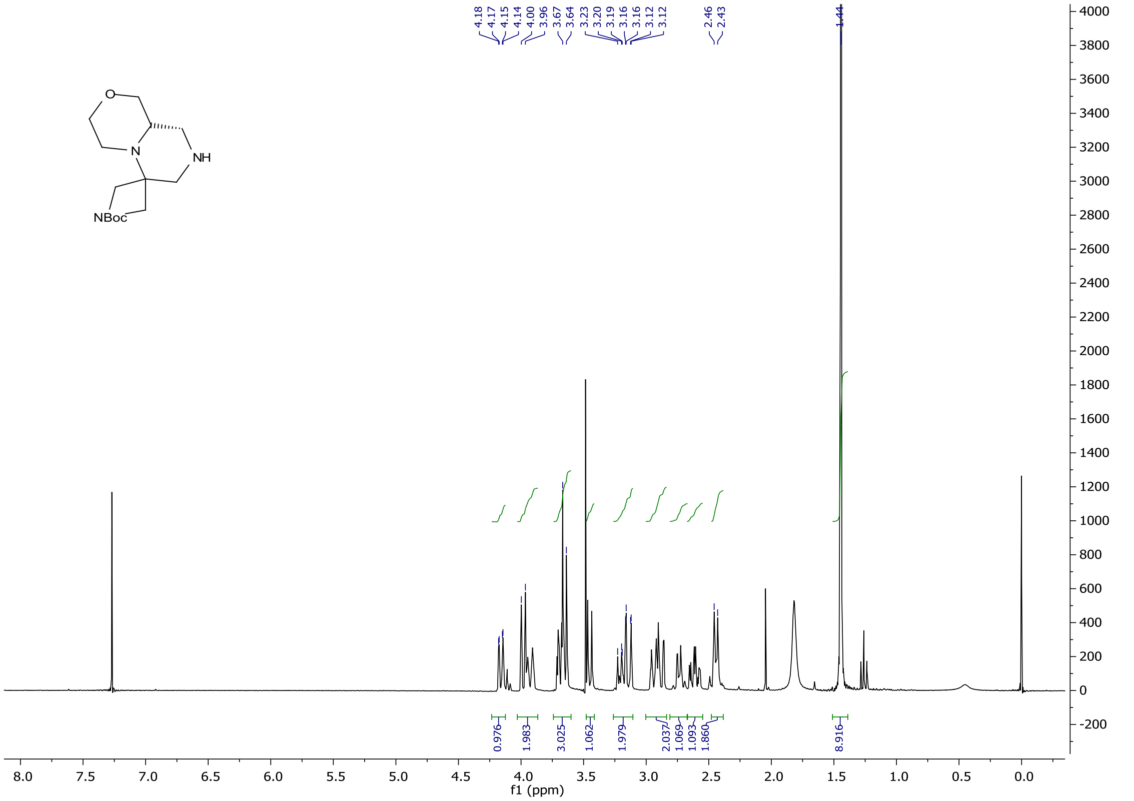
4.18 4.17 4.15 4.14 4.00 3.96 3.67 3.64 3.23 3.20 3.19 3.16 3.16 3.12 3.12 2.46 2.43 1.44
0.976 1.983 3.025 1.062 1.979 2.037 1.069 1.093 1.860 8.916
O
N
NH
NBoc
f1 (ppm)

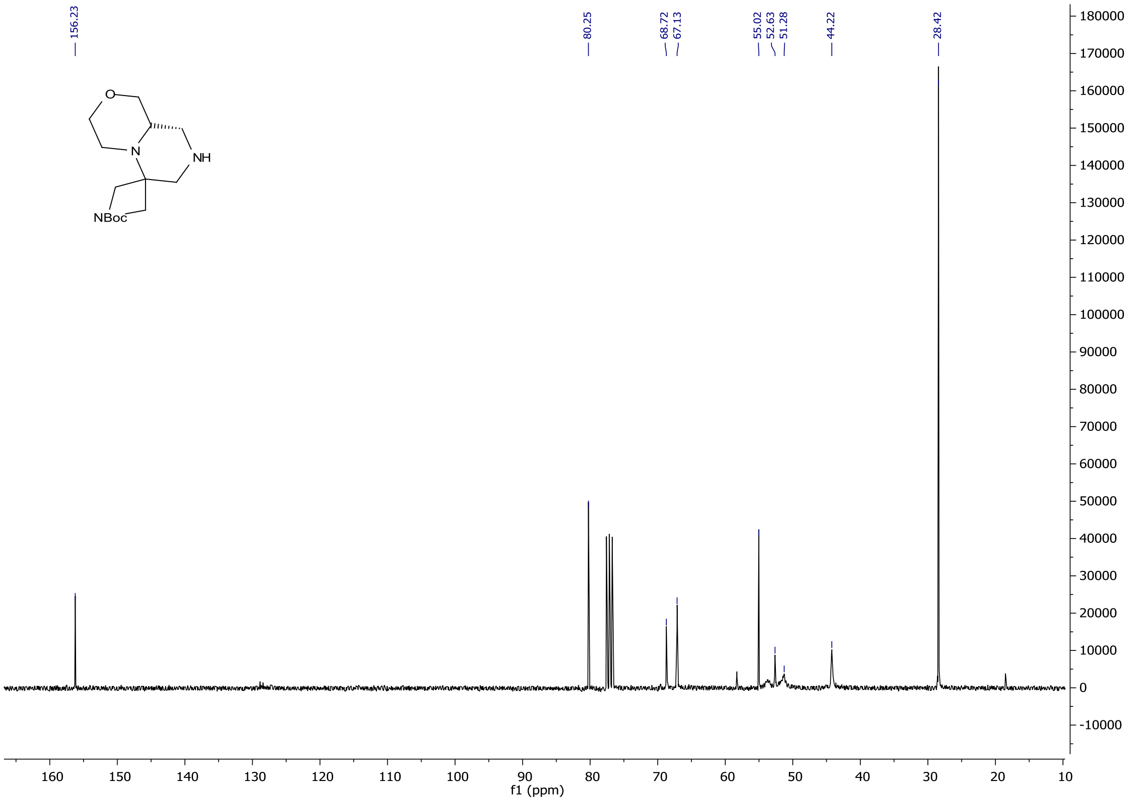
156.23 80.25 68.72 67.13 55.02 52.63 51.28 44.22 28.42
O
N
NH
NBoc
f1 (ppm)

Compound 11

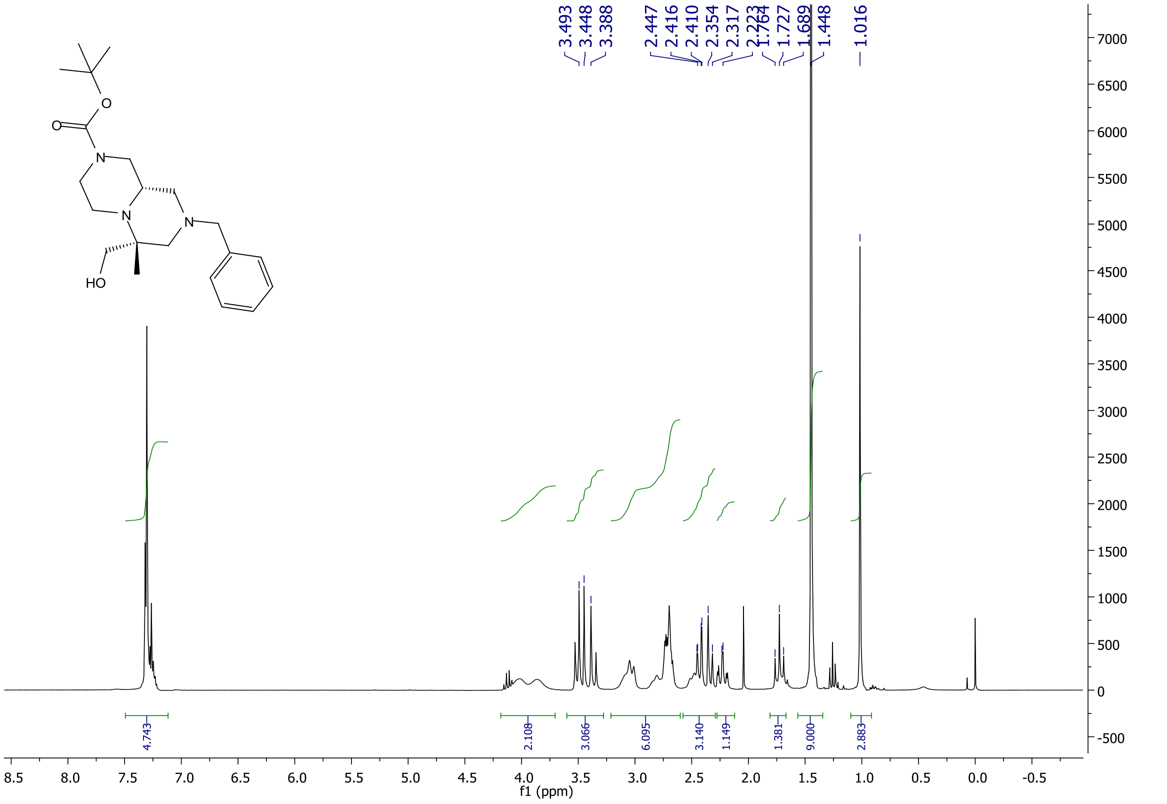

3.493
3.448
3.388
2.447
2.416
2.410
2.354
2.317
2.223
1.764
1.727
1.689
1.448
1.016
4.743
2.108
3.066
6.095
3.140
1.149
1.381
9.000
2.883
f1 (ppm)

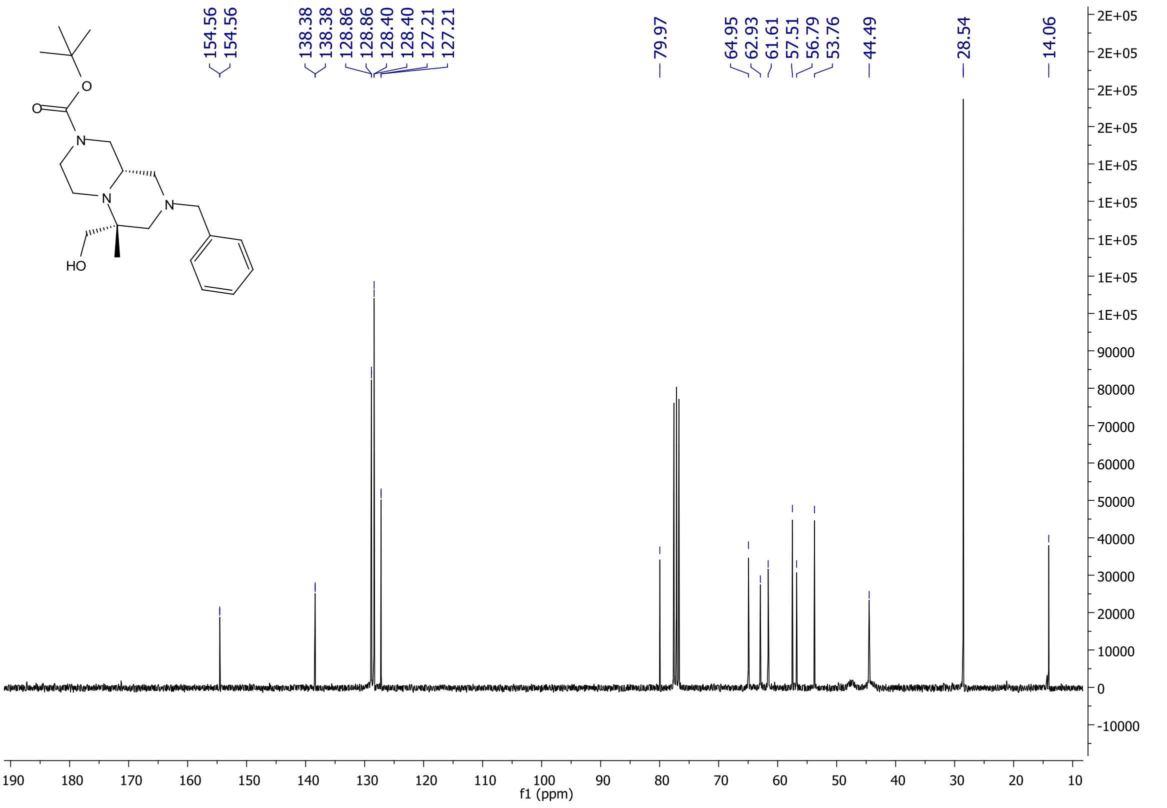

154.56
154.56
138.38
138.38
128.86
128.86
128.40
128.40
127.21
127.21
79.97
64.95
62.93
61.61
57.51
56.79
53.76
44.49
28.54
14.06
f1 (ppm)

Compound 6{3,4}

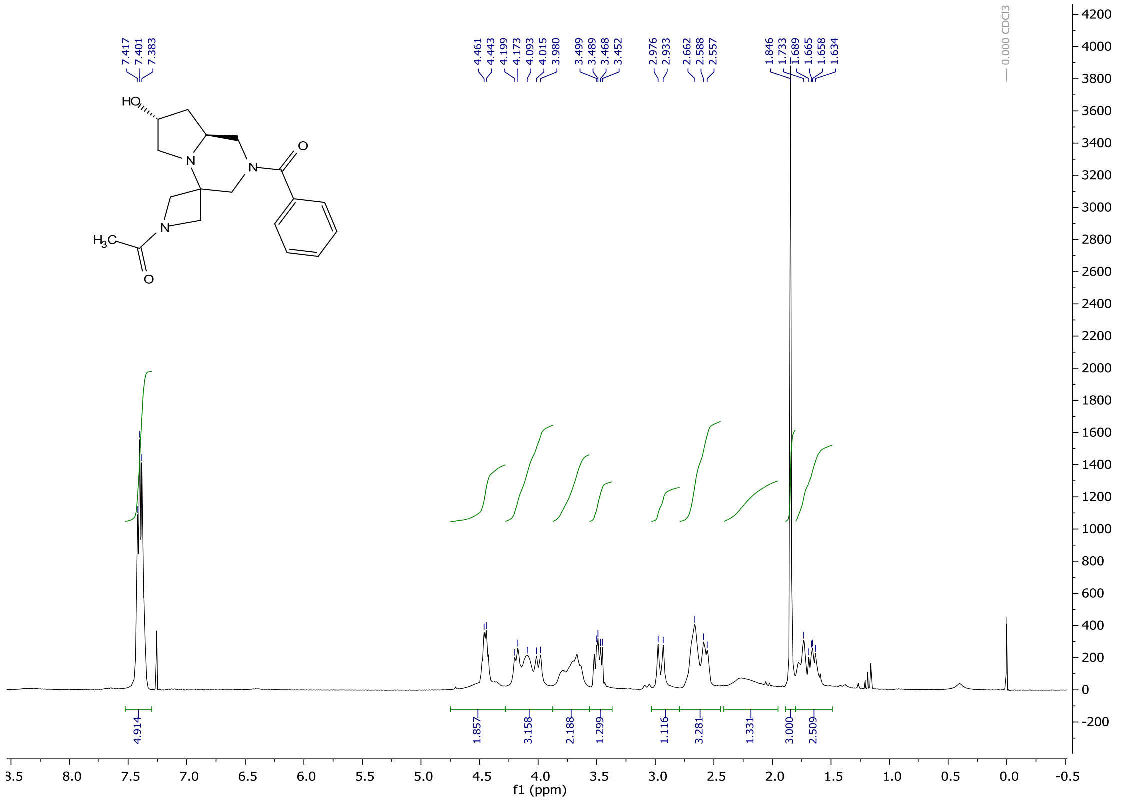

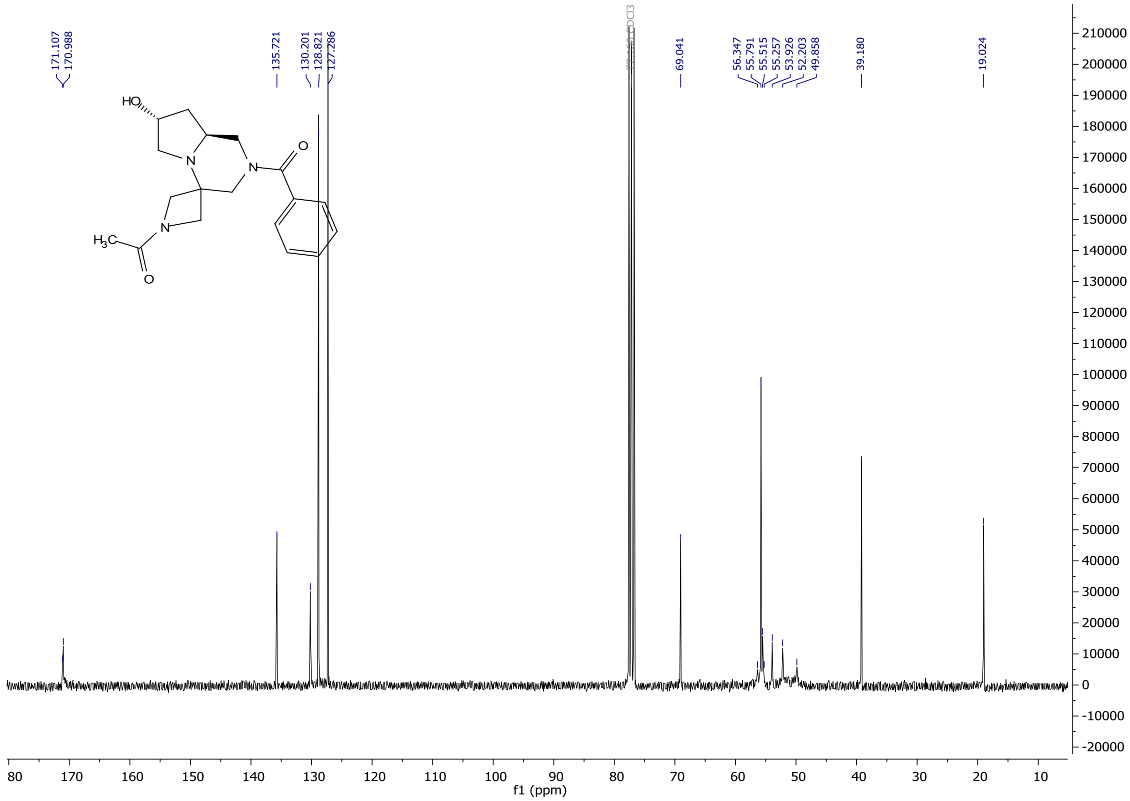

Compound 6{4,5}

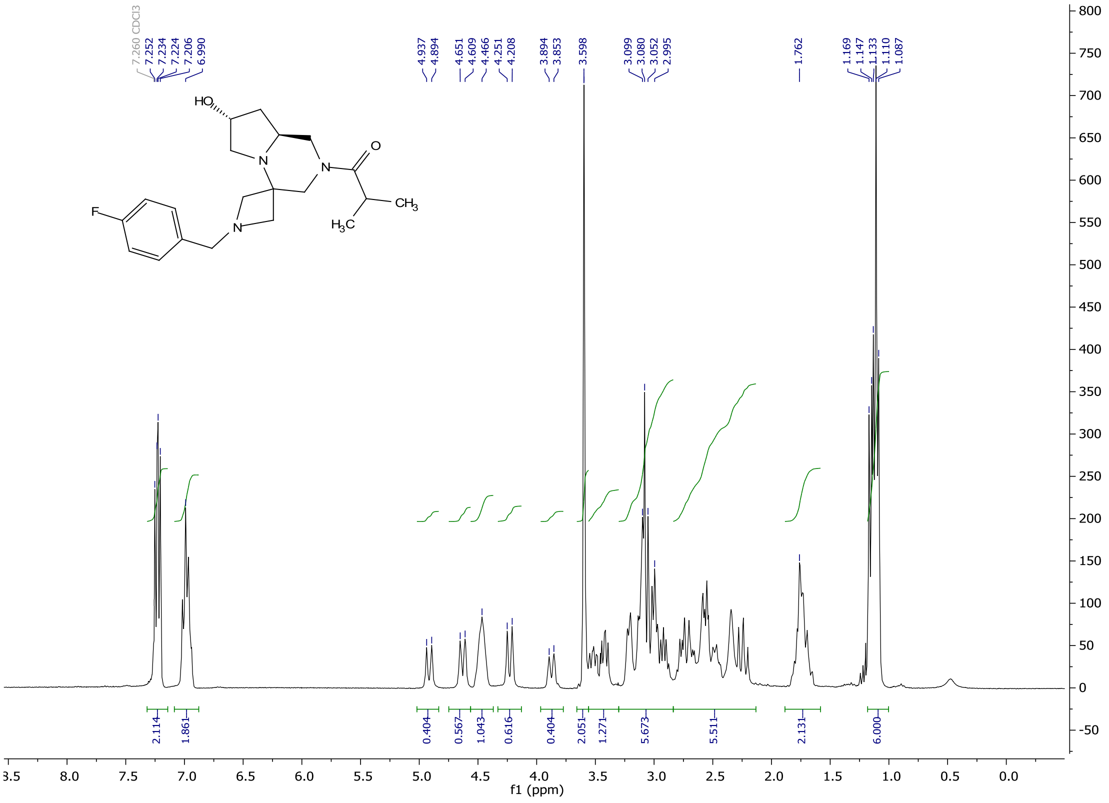

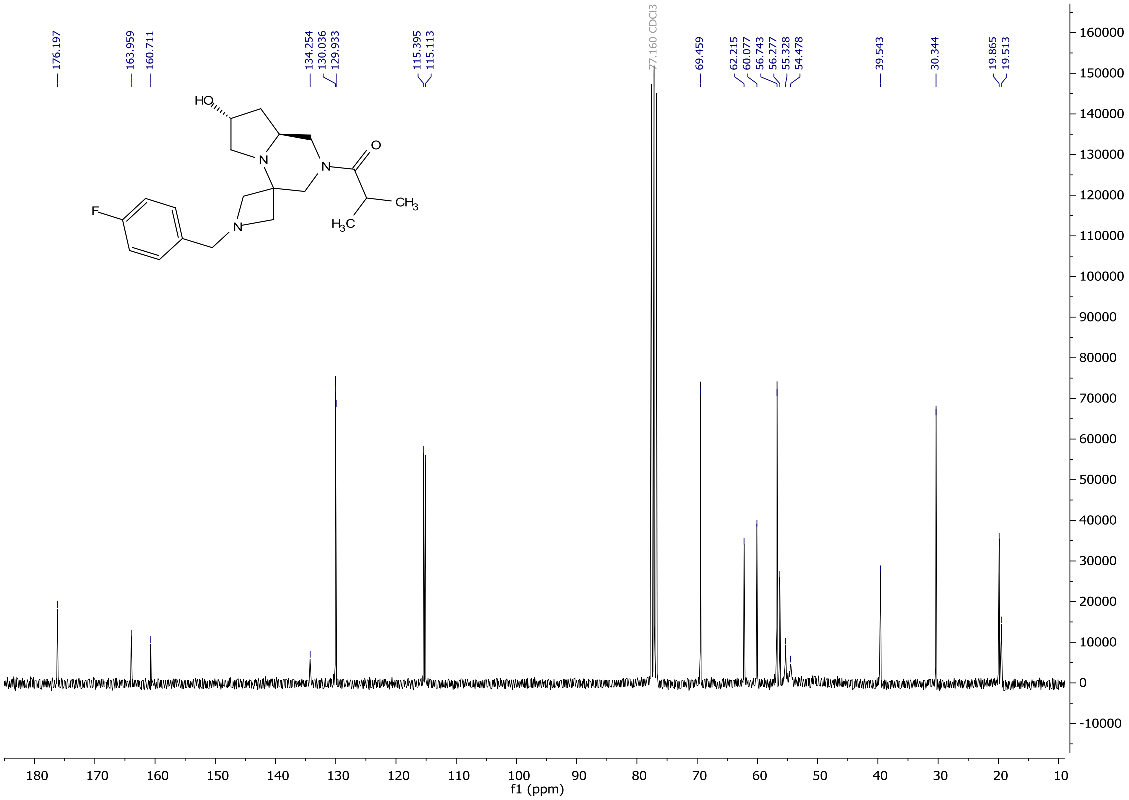

Compound 13{8,1}

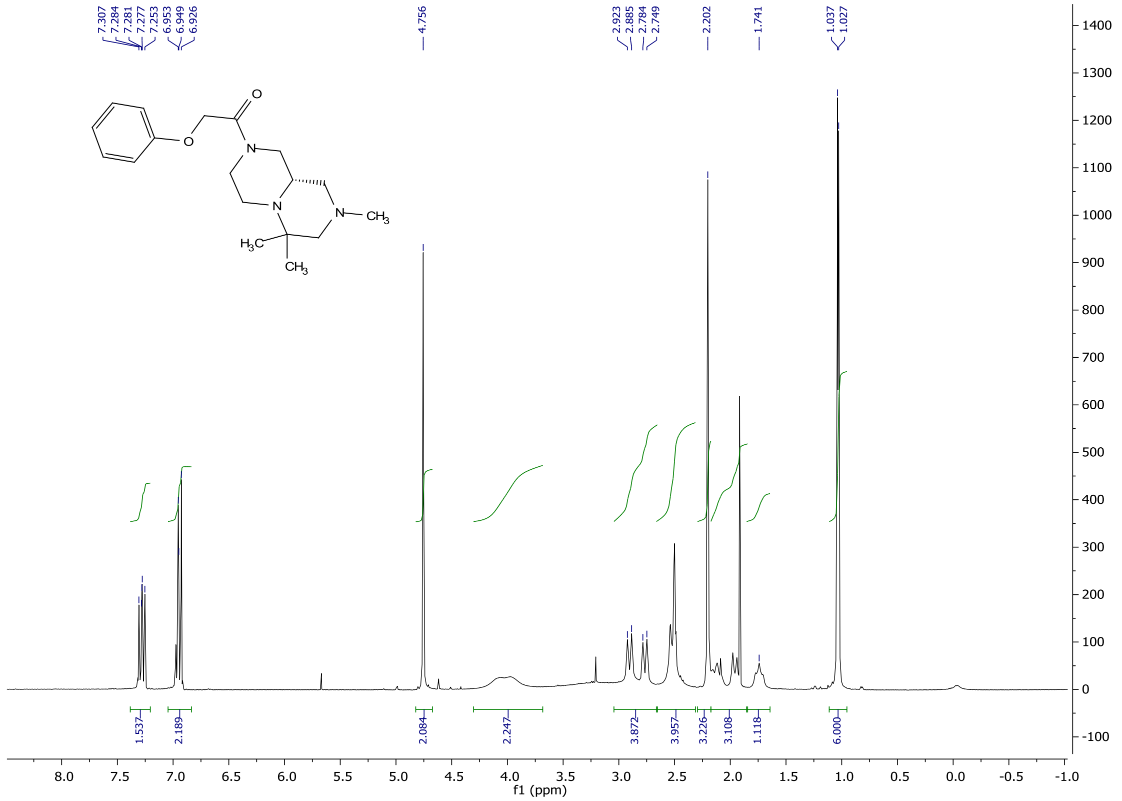

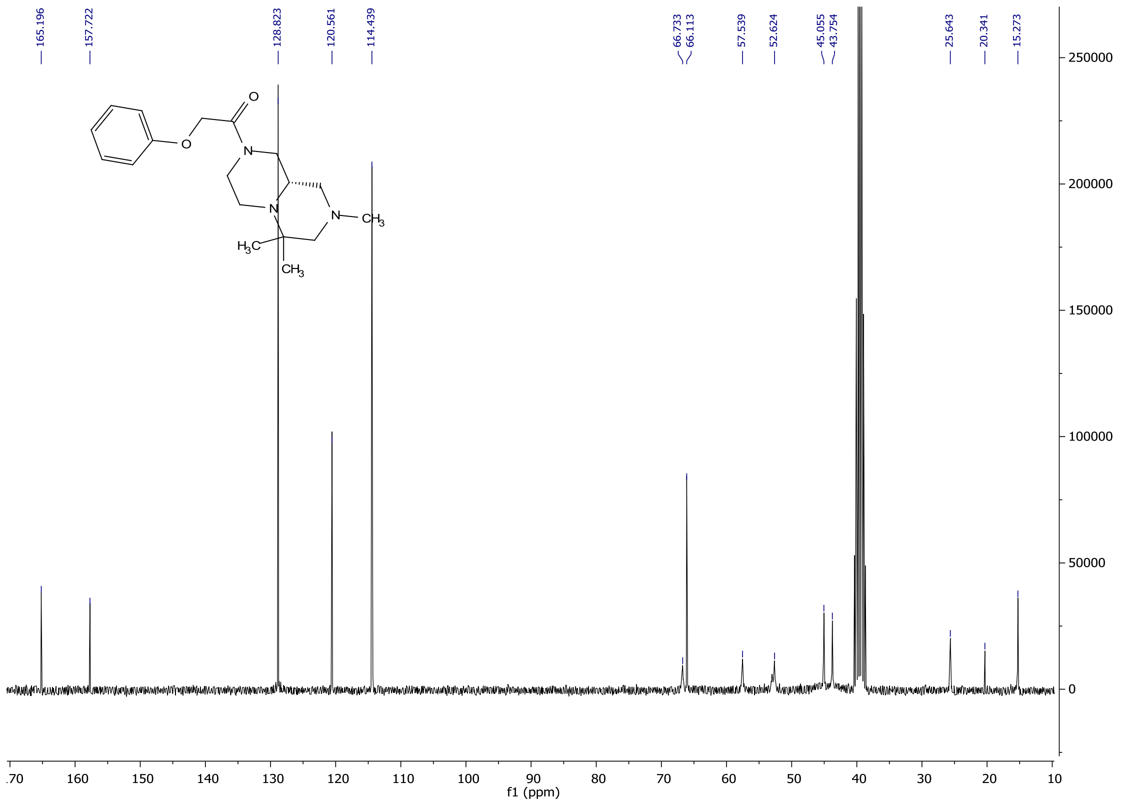

Compound 13{6,2}

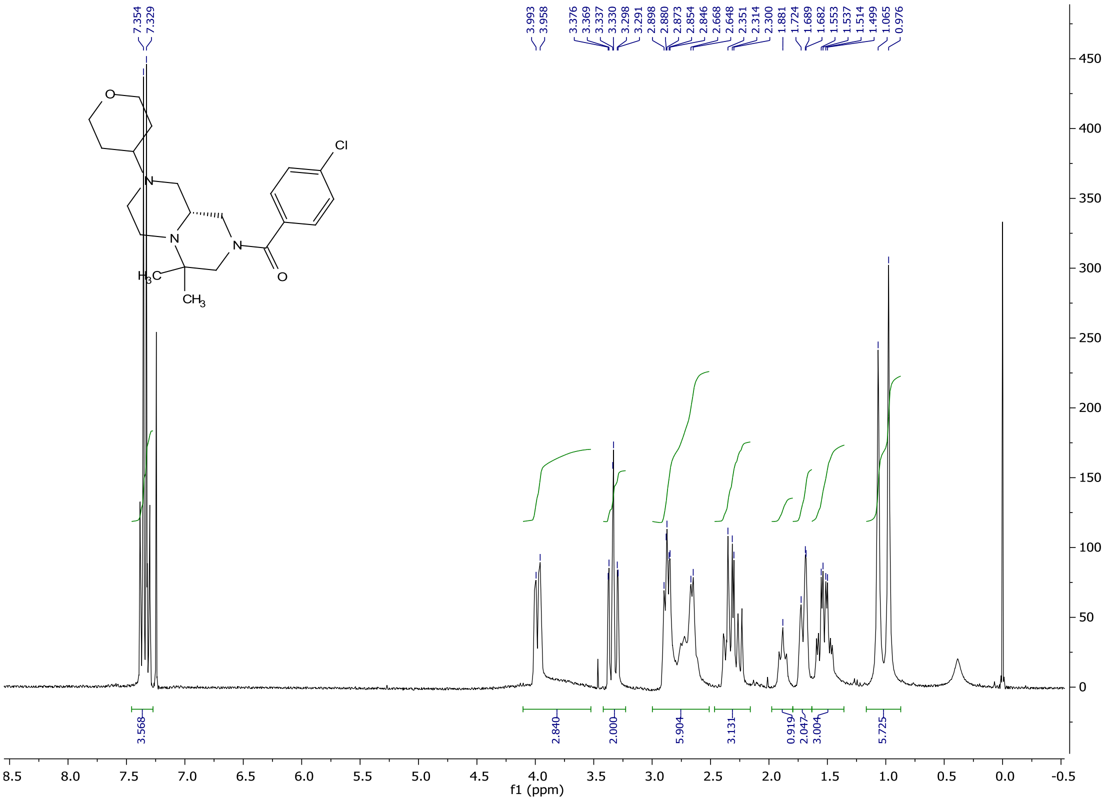

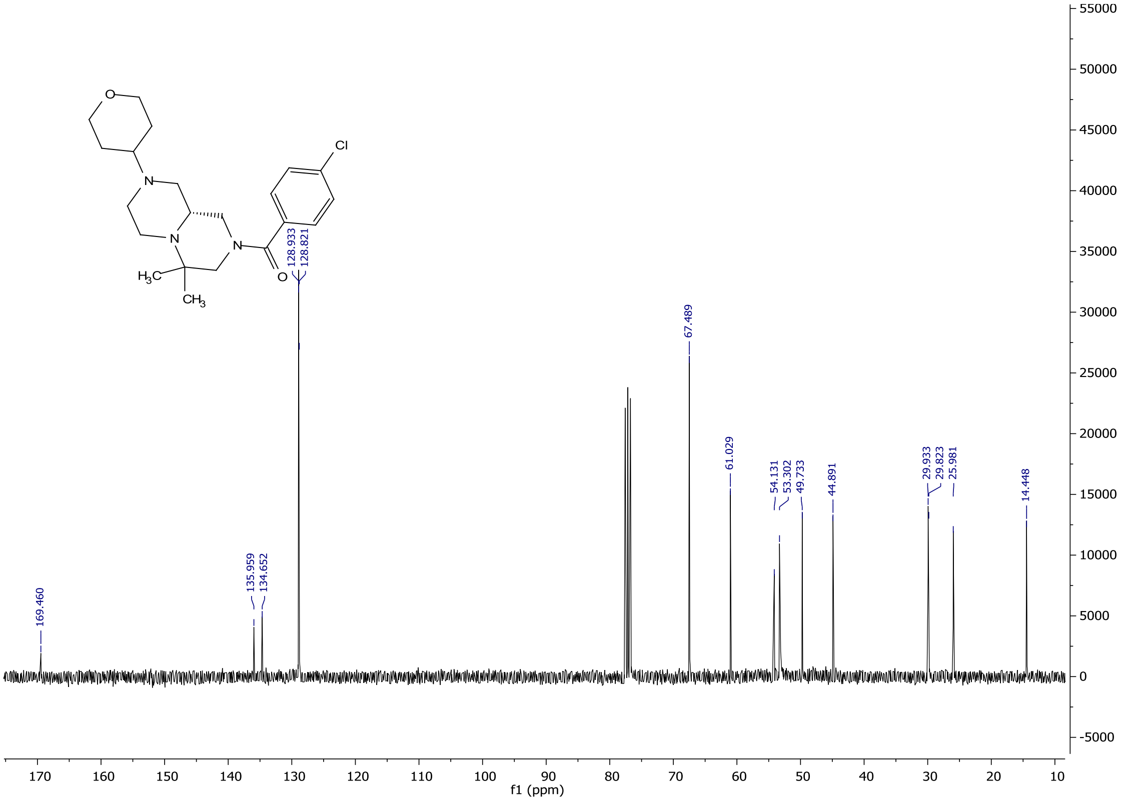

Compound 15{8,4}

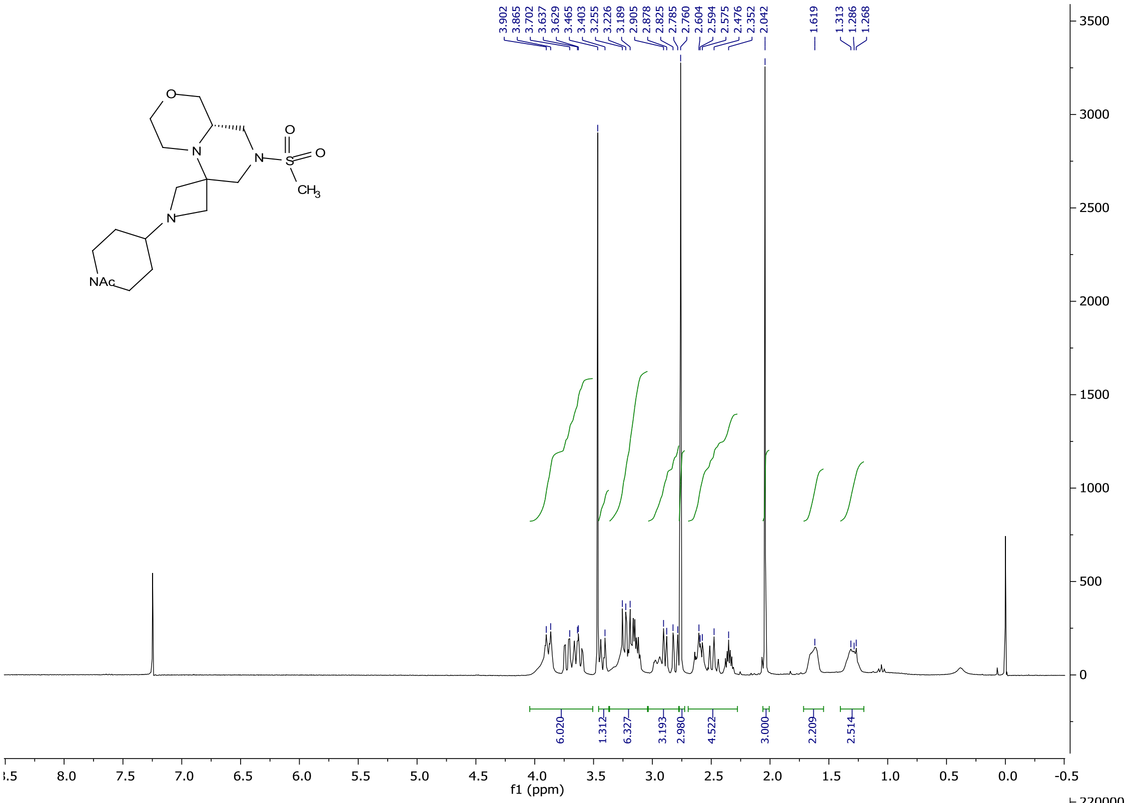

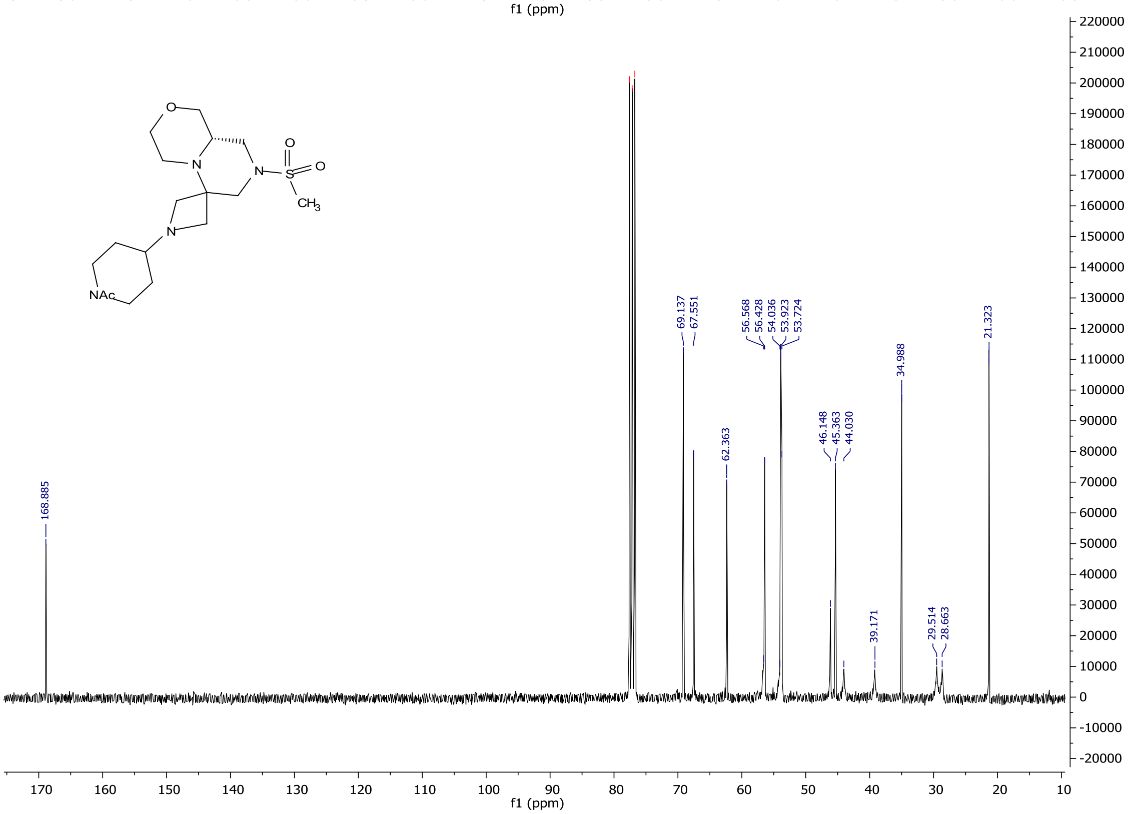

Compound 15{6,3}

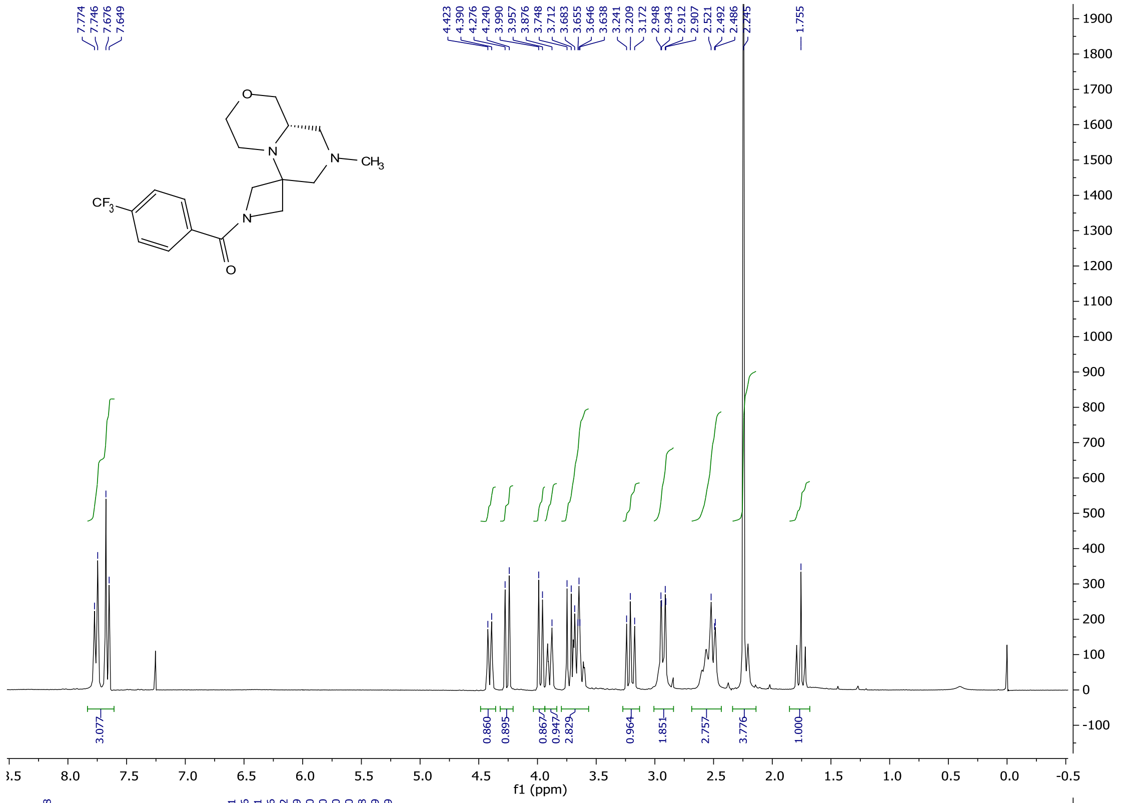

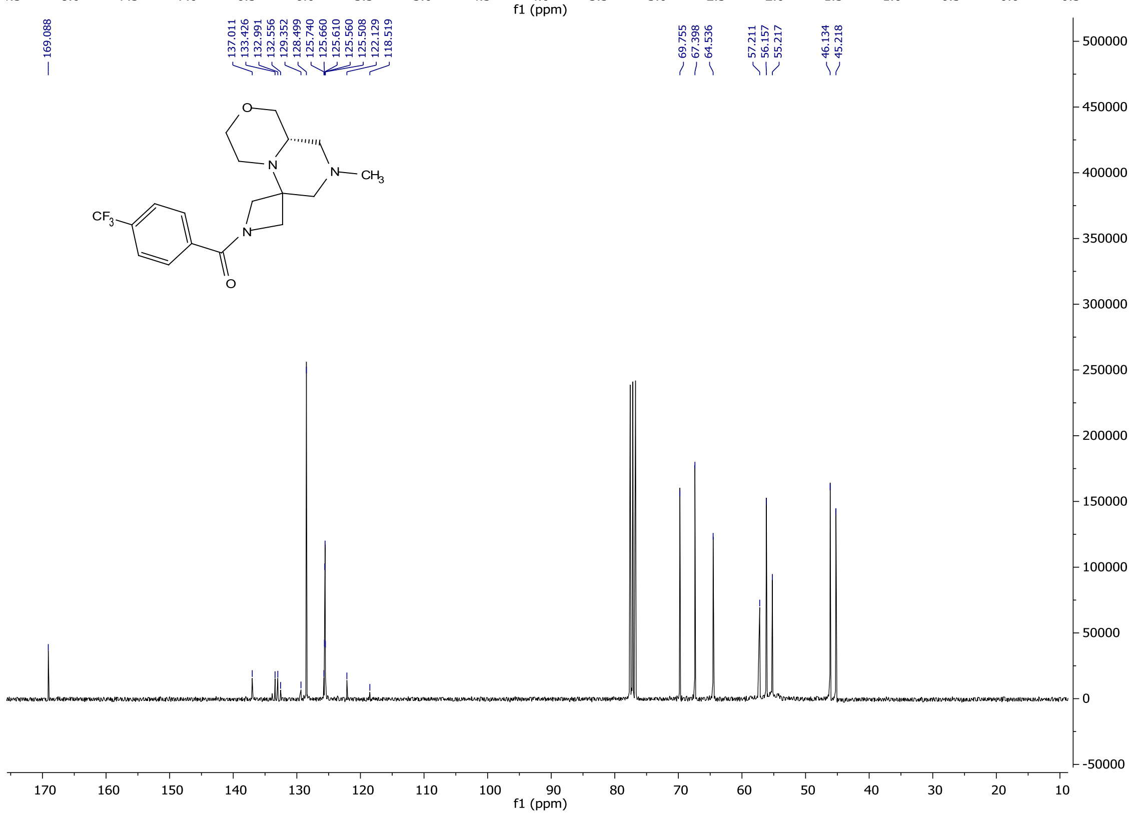

Compound 5{3}

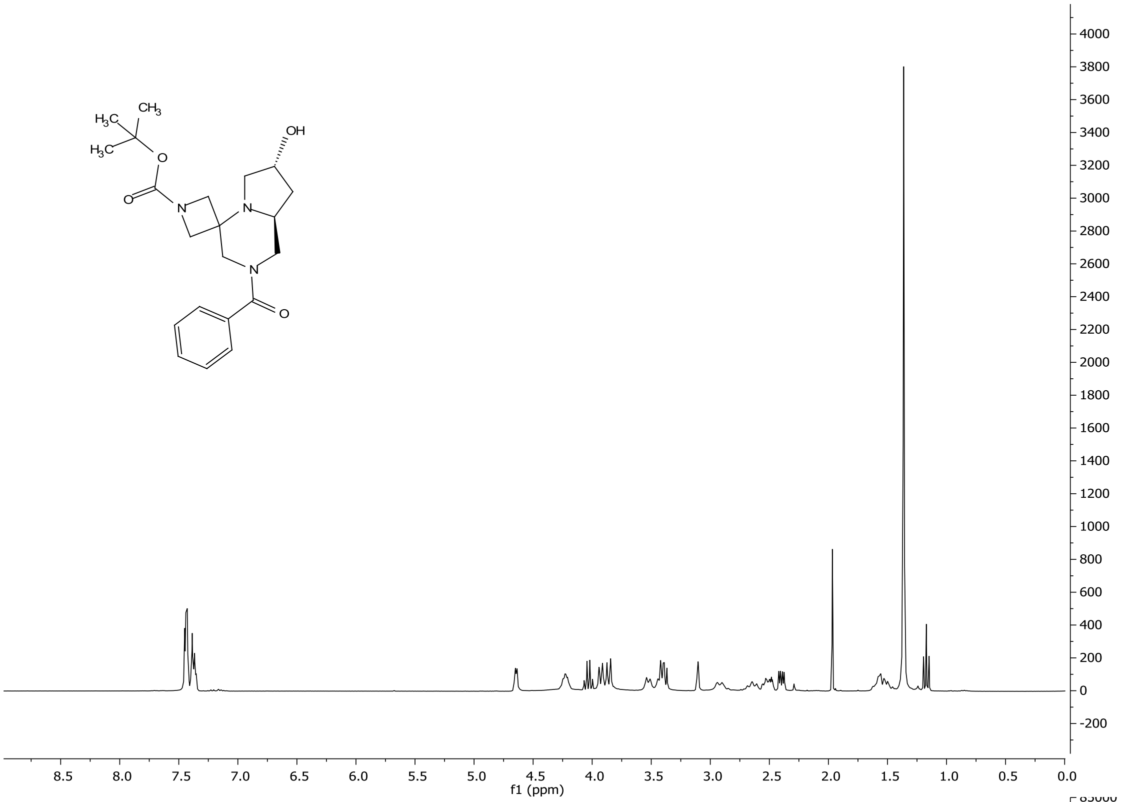

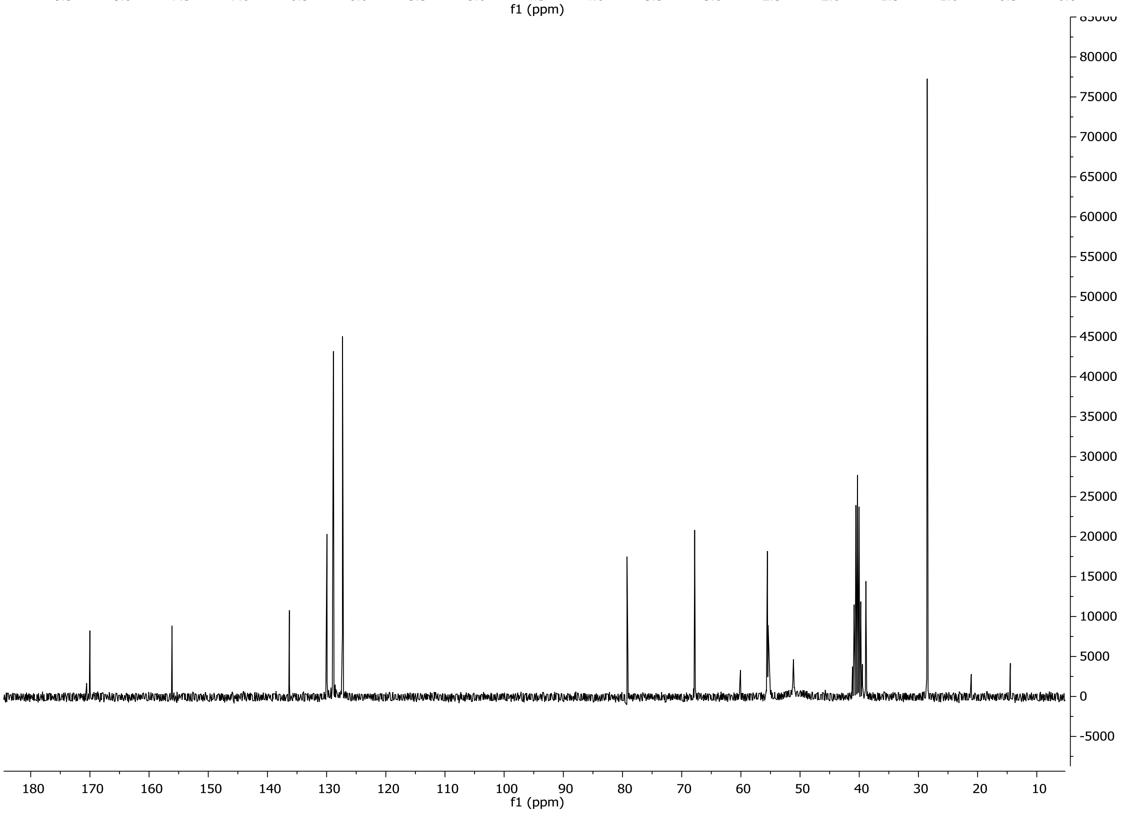

Compound 5{4}

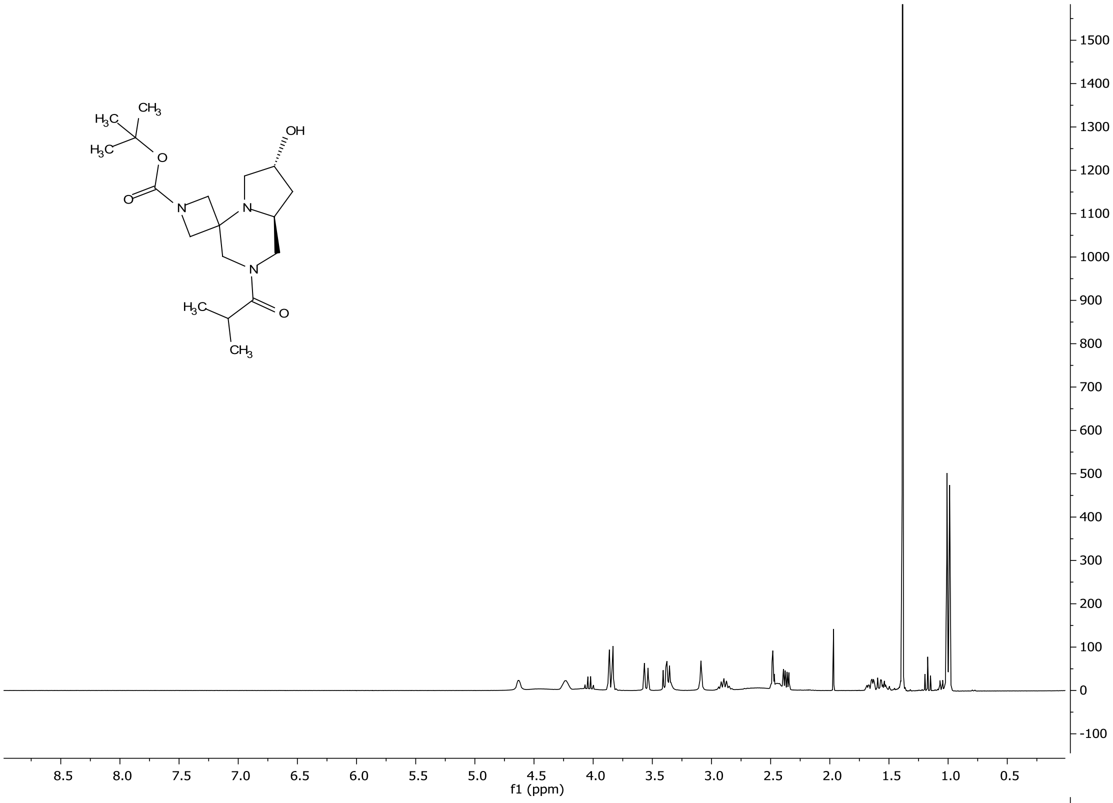

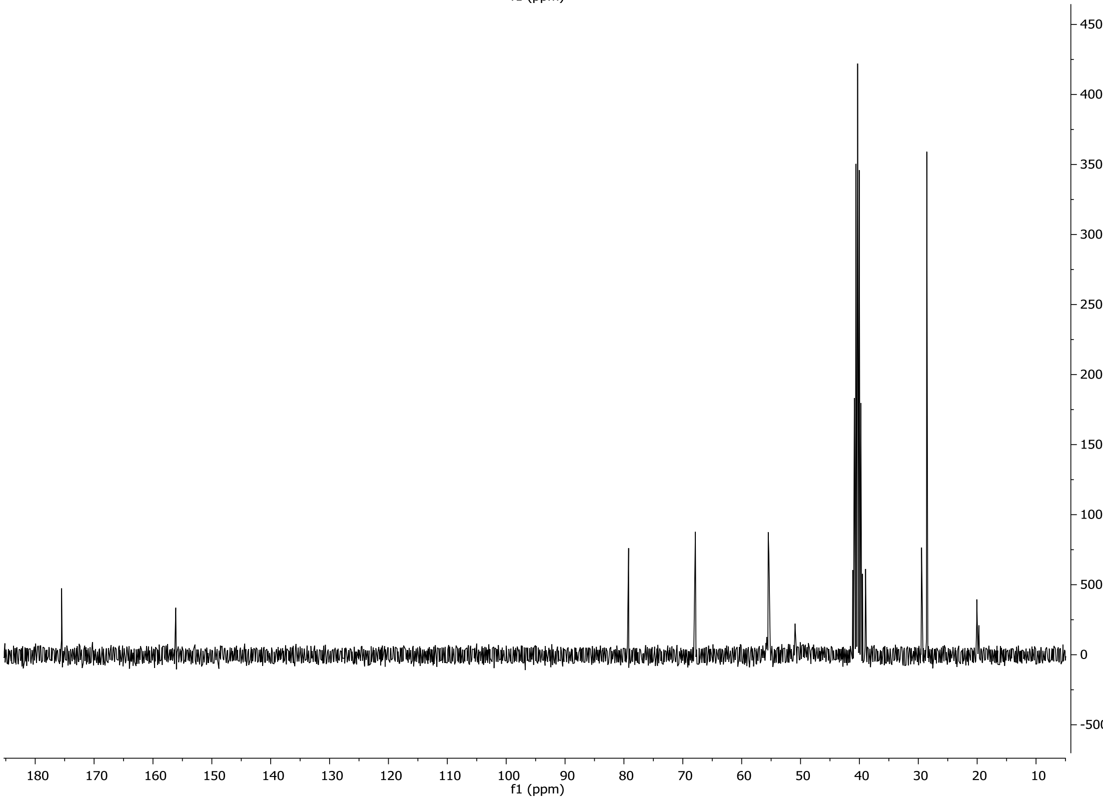

Compound 5{8}

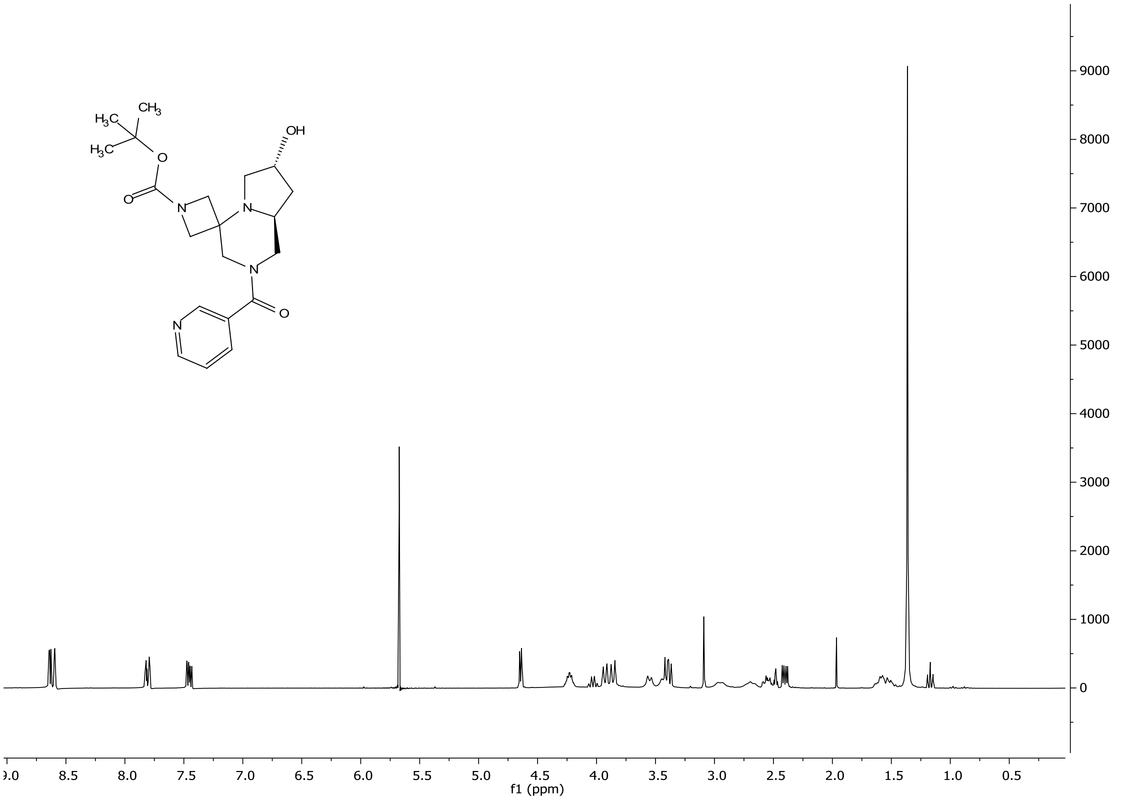

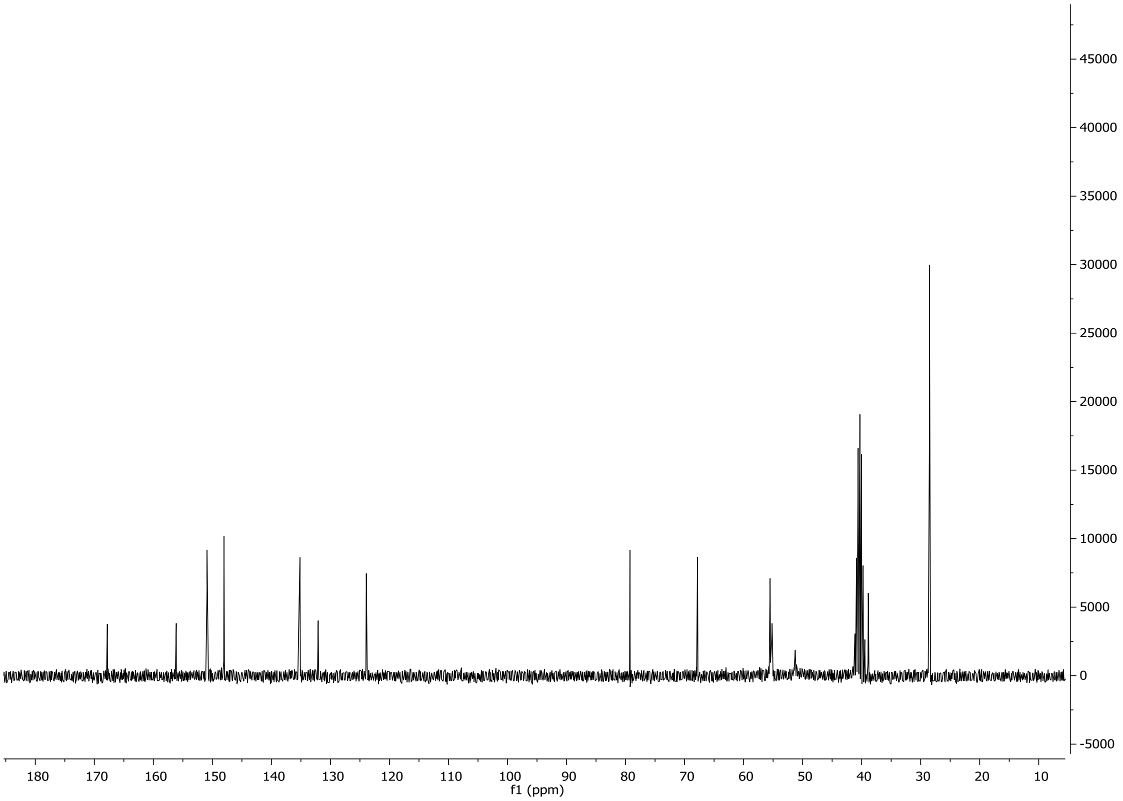

Compound 5{9}

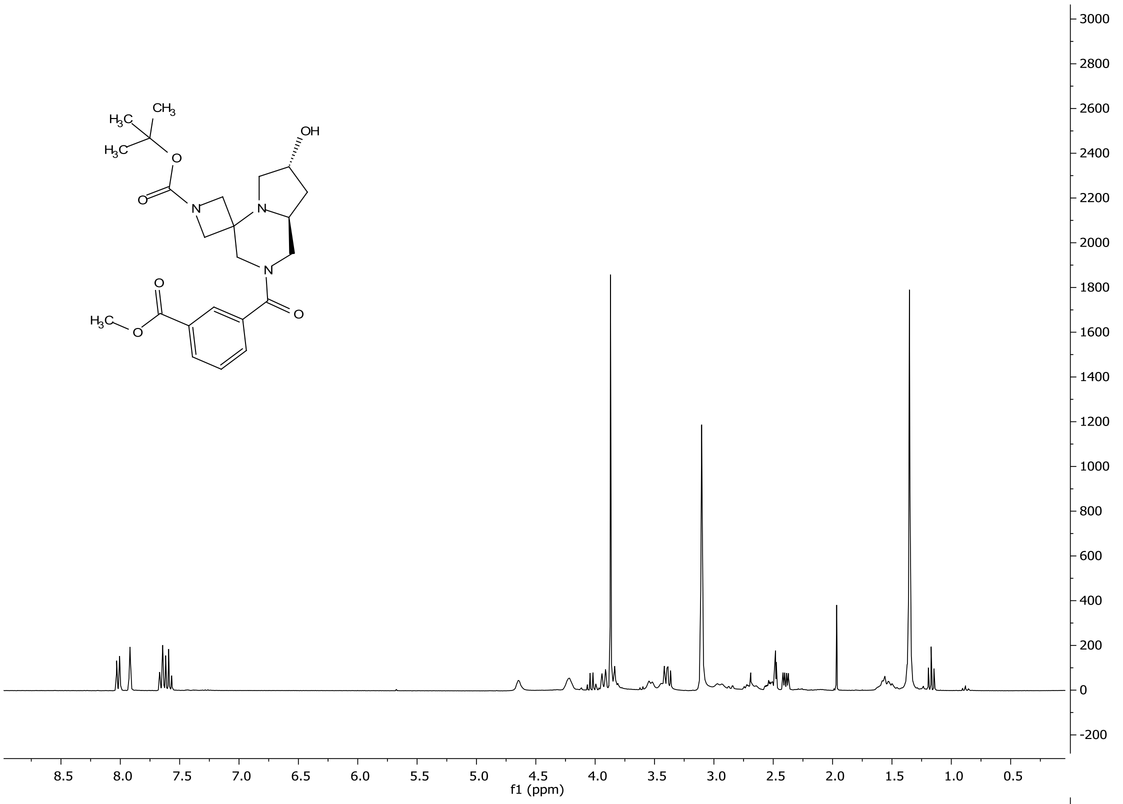

H3C
CH3
H3C
O
O
N
N
OH
N
O
O
H3C
O
O
3000
2800
2600
2400
2200
2000
1800
1600
1400
1200
1000
800
600
400
200
0
-200
8.5 8.0 7.5 7.0 6.5 6.0 5.5 5.0 4.5 4.0 3.5 3.0 2.5 2.0 1.5 1.0 0.5
f1 (ppm)

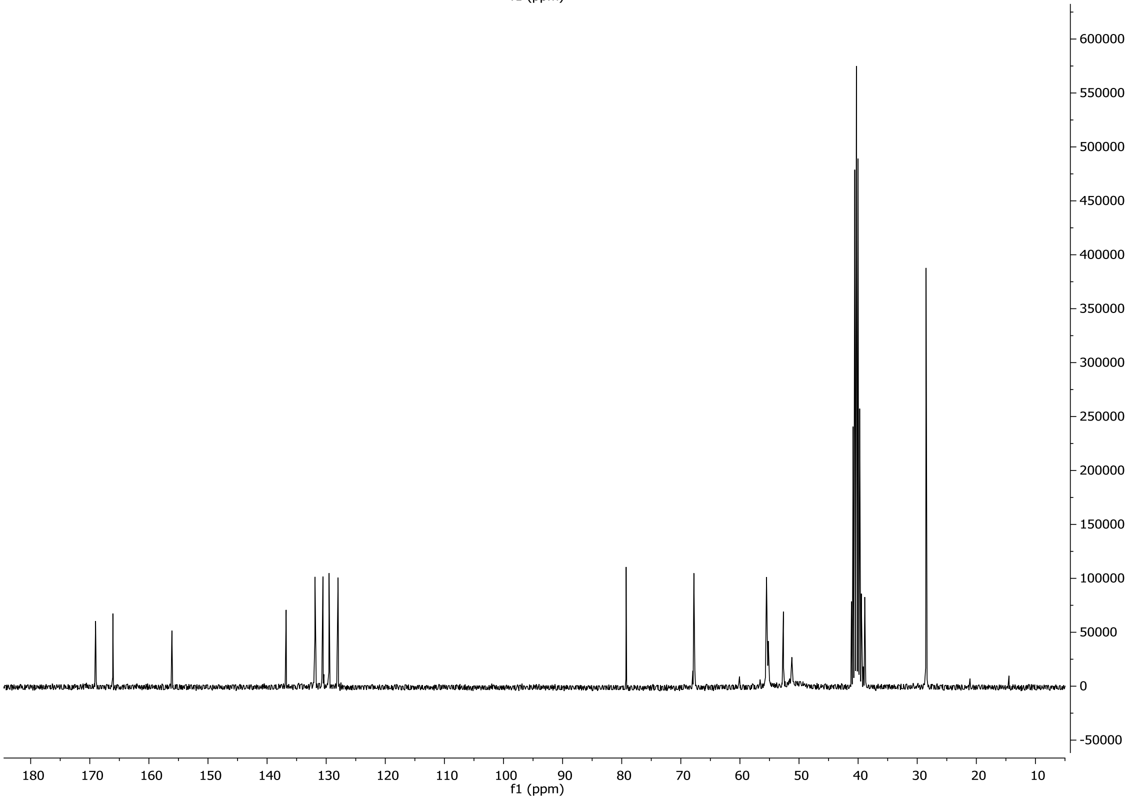

600000
550000
500000
450000
400000
350000
300000
250000
200000
150000
100000
50000
0
-50000
180 170 160 150 140 130 120 110 100 90 80 70 60 50 40 30 20 10
f1 (ppm)

Compound 5{7}

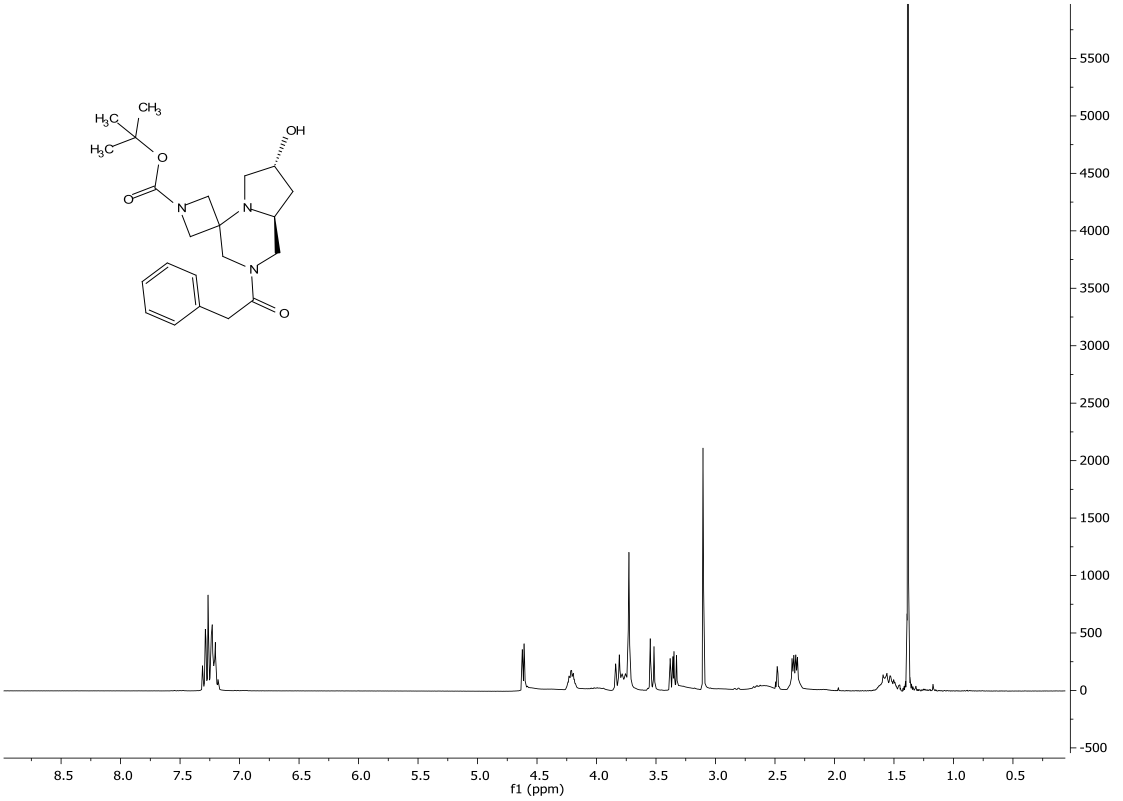

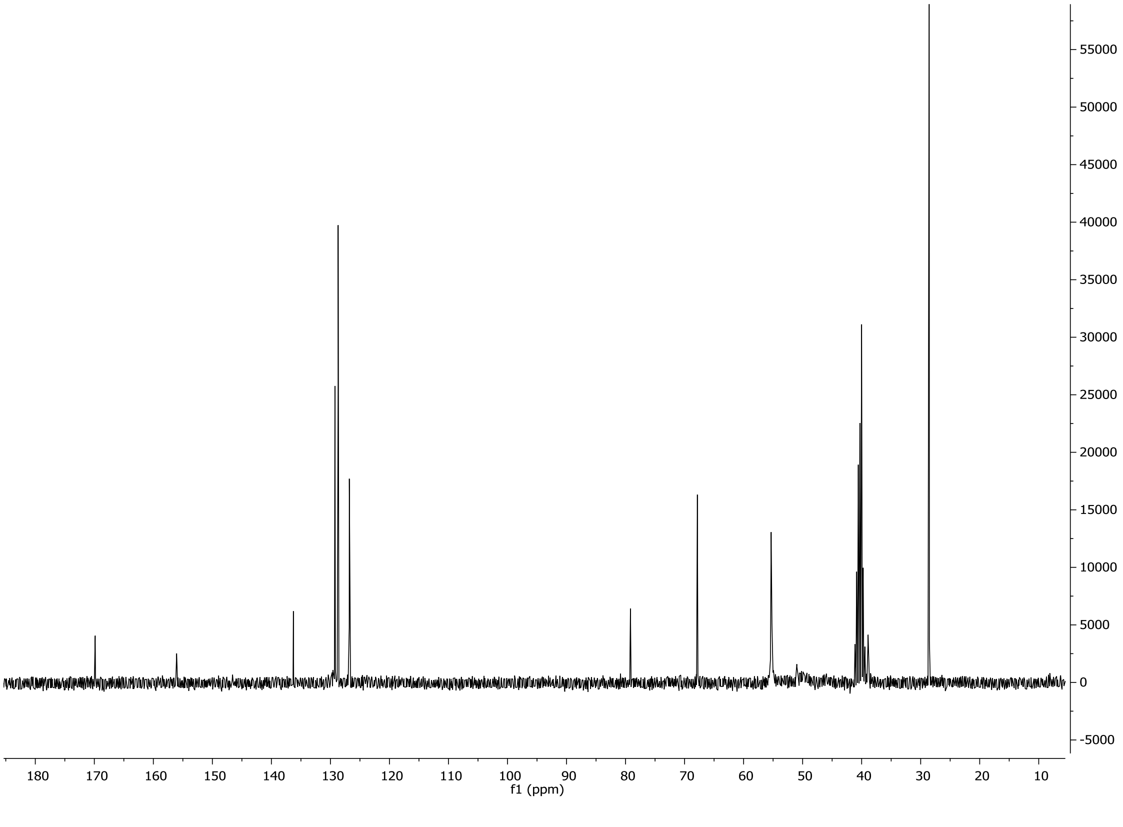

Compound 5{10}

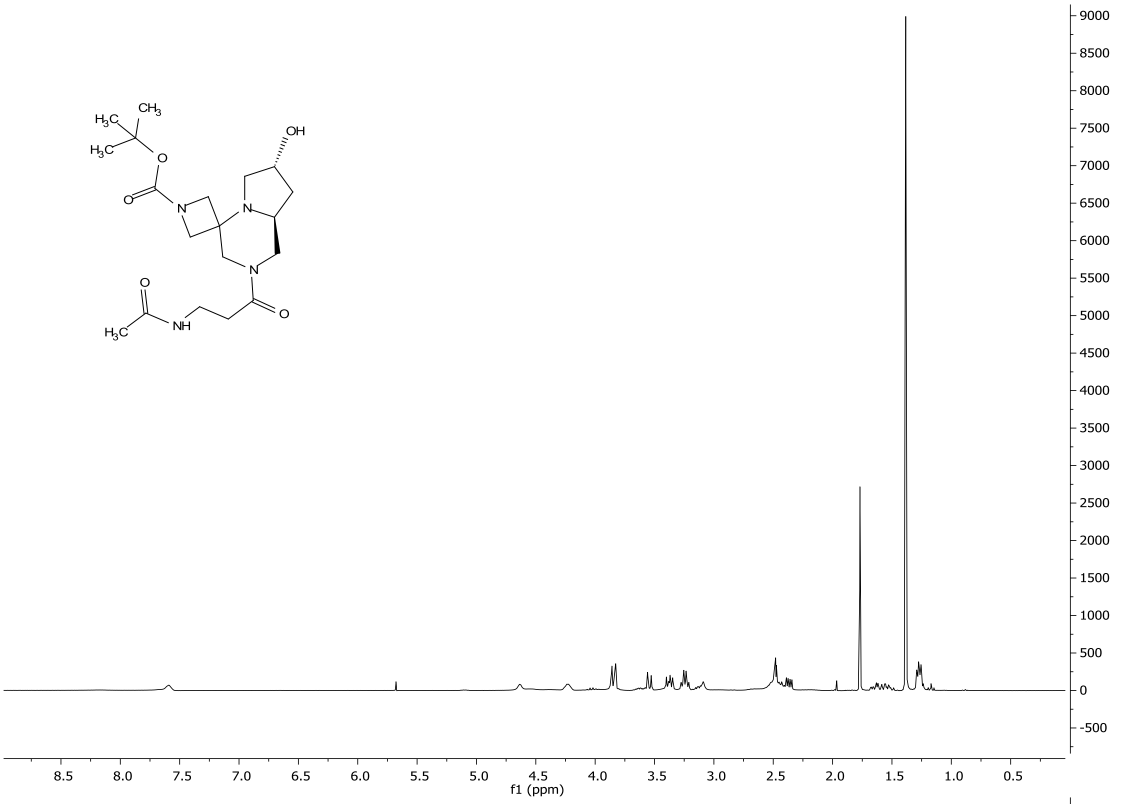

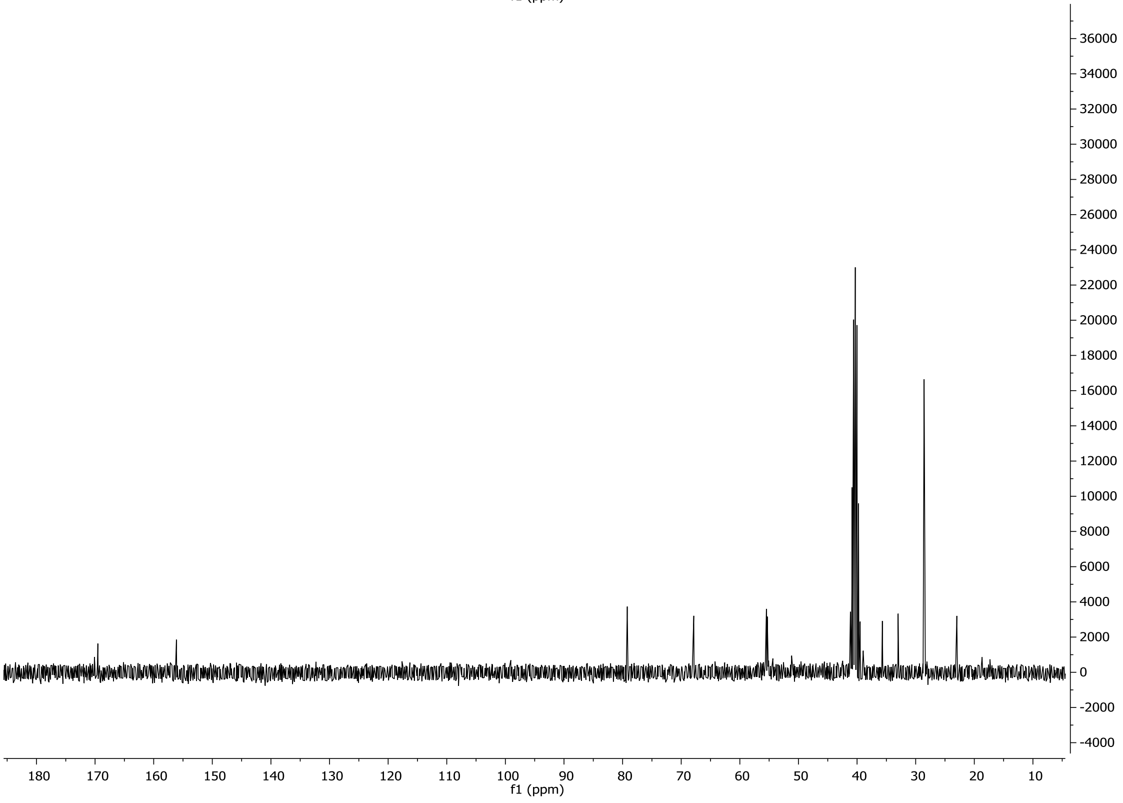

Compound 5{6}

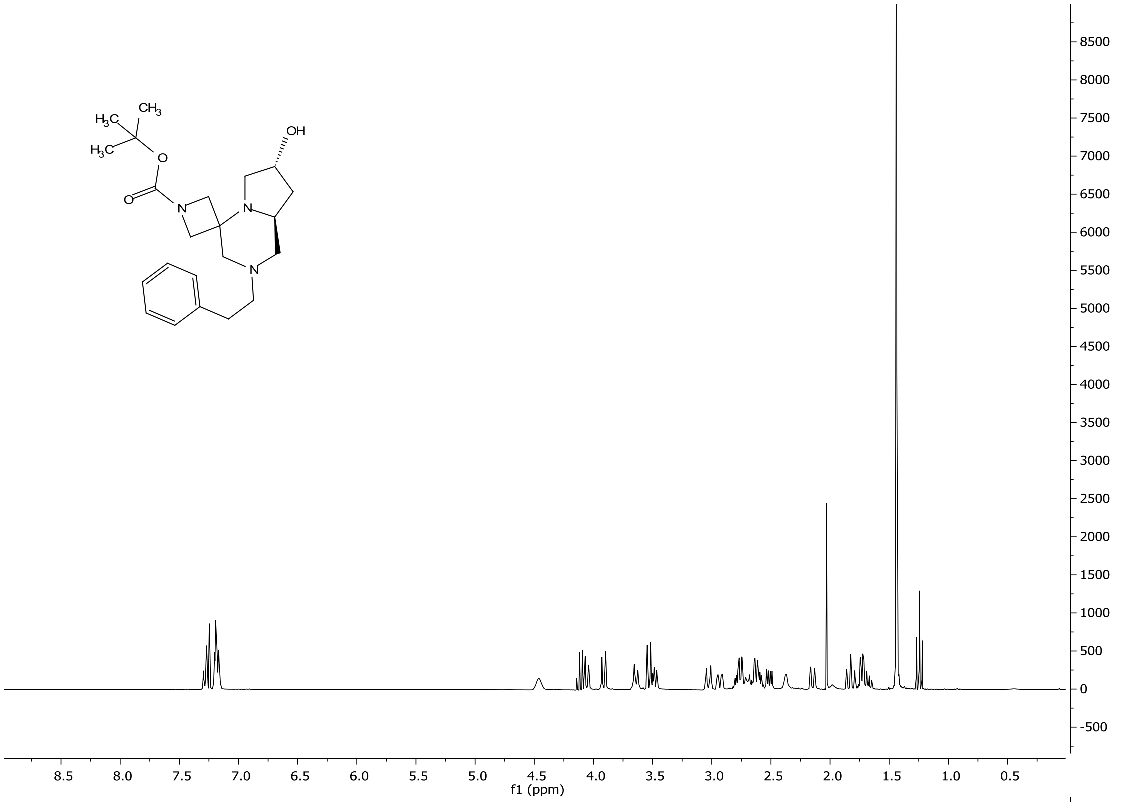

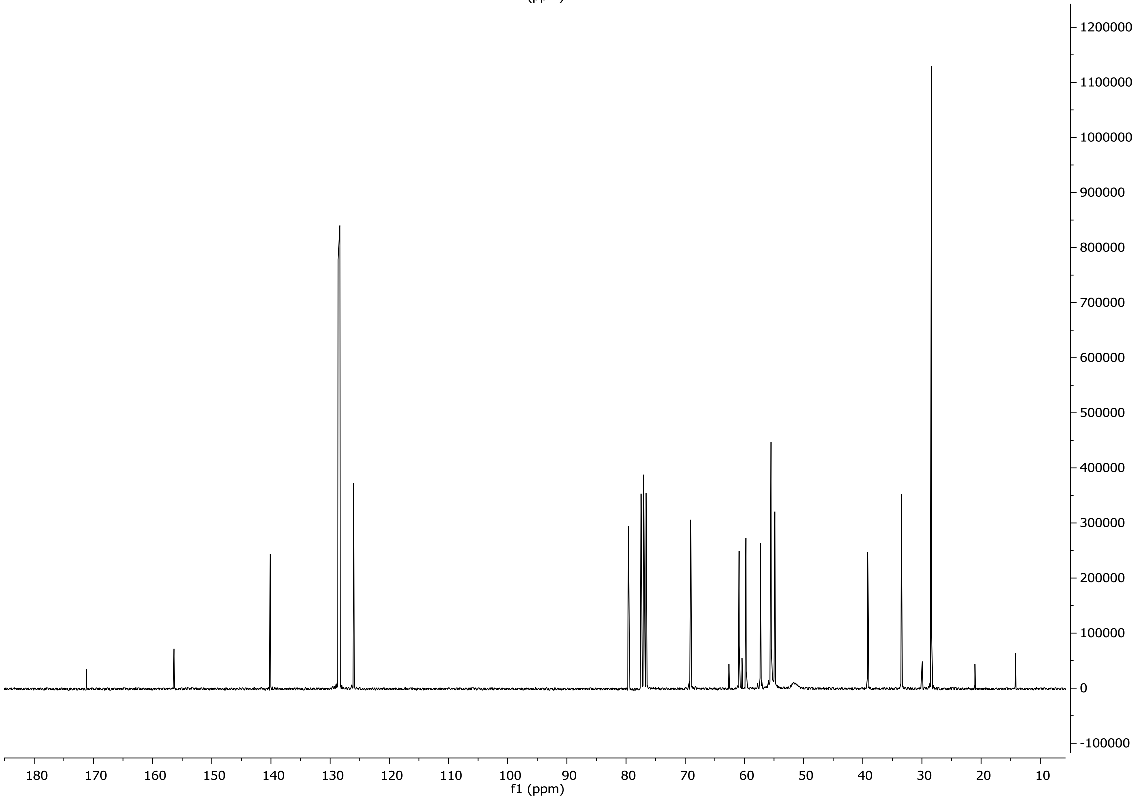

Compound 5{5}

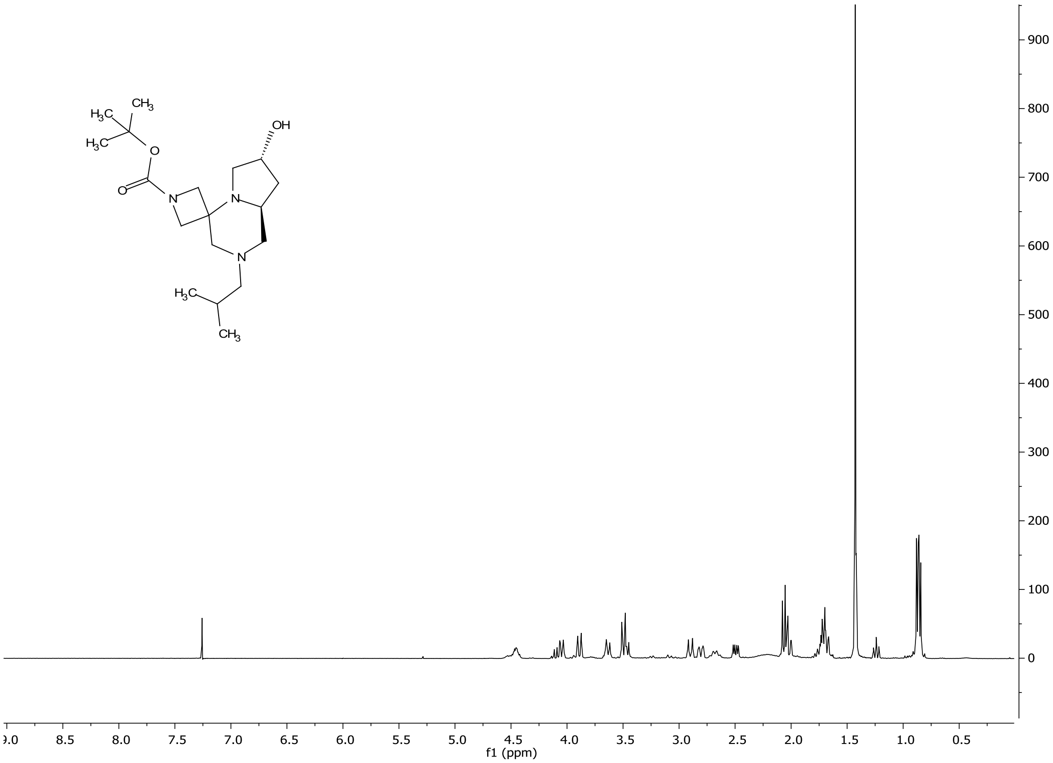

Compound 5{1}

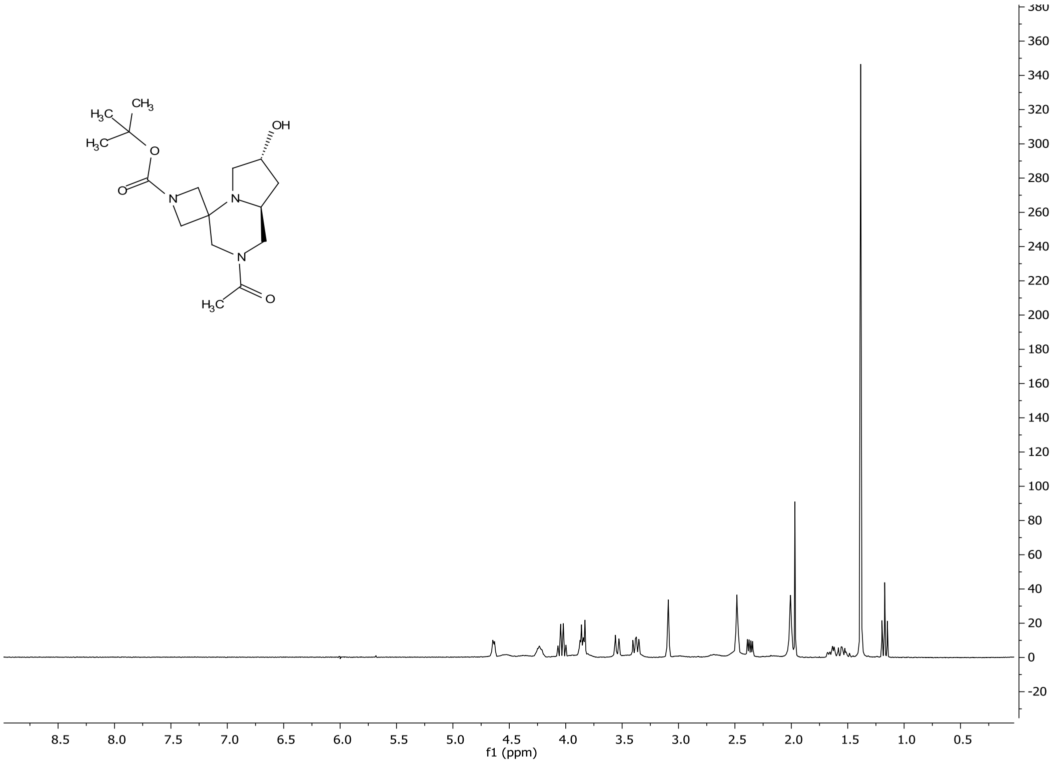

Compound 5{2}

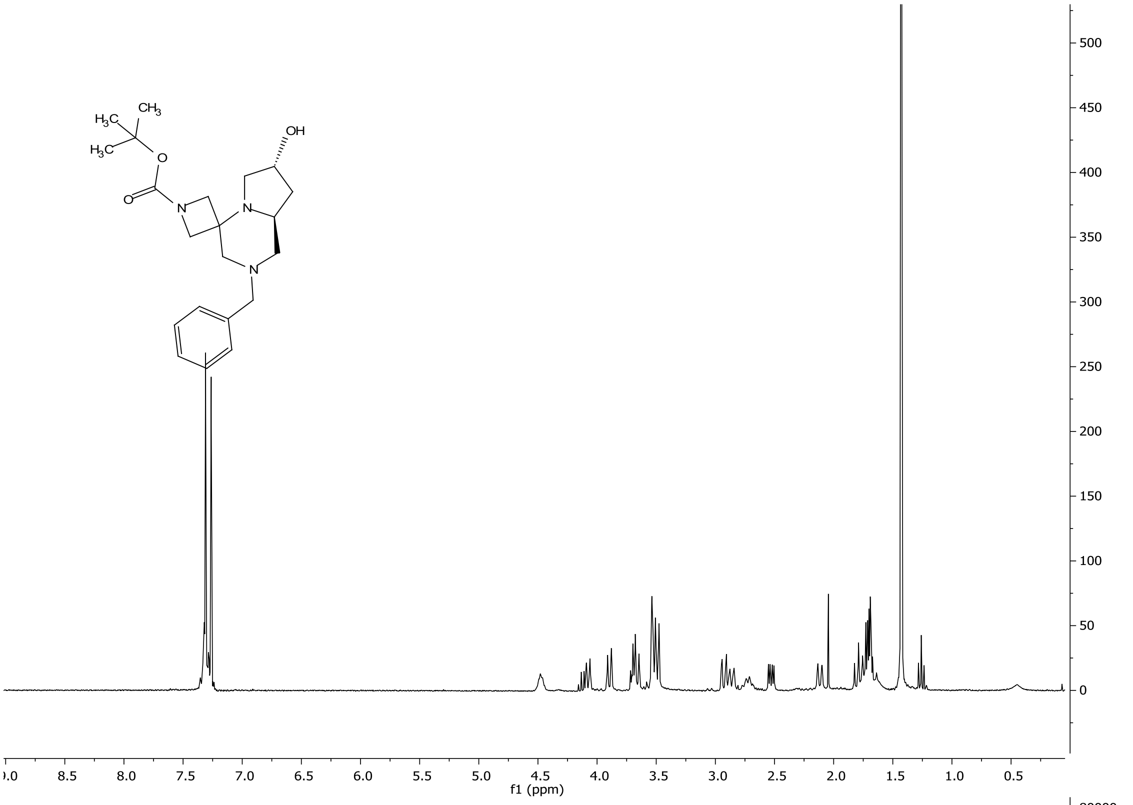

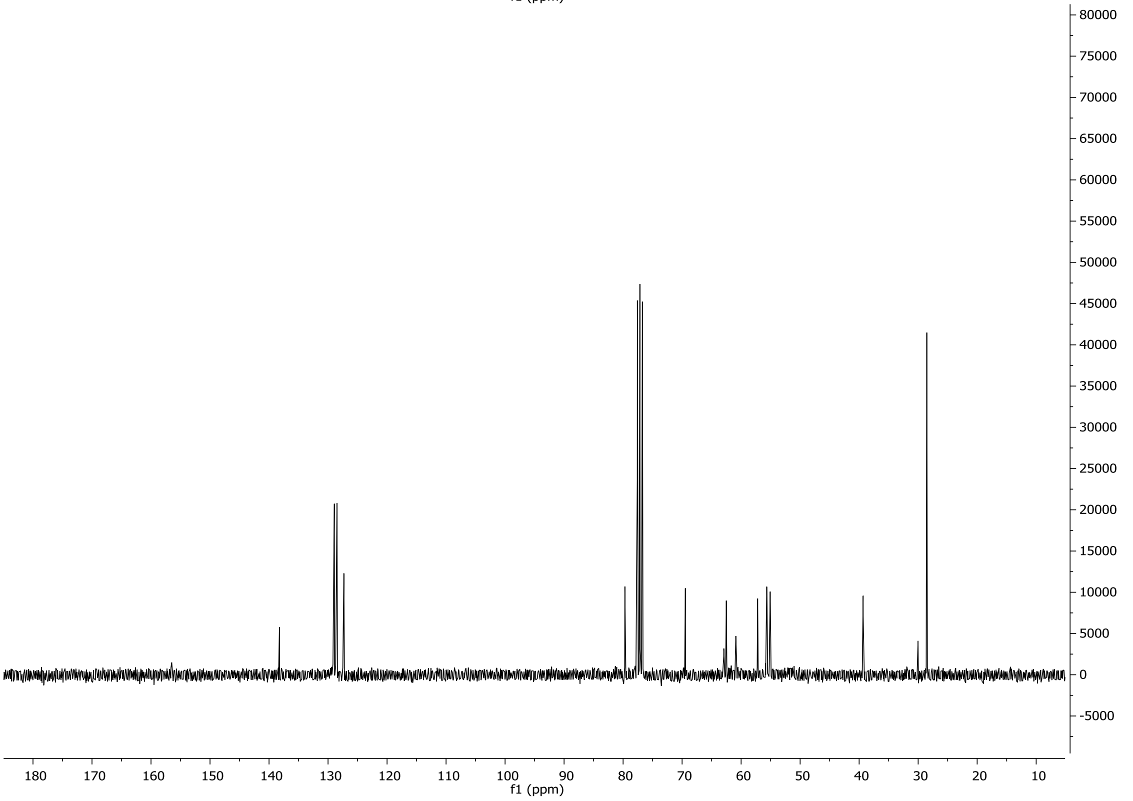

Compound 12{6}

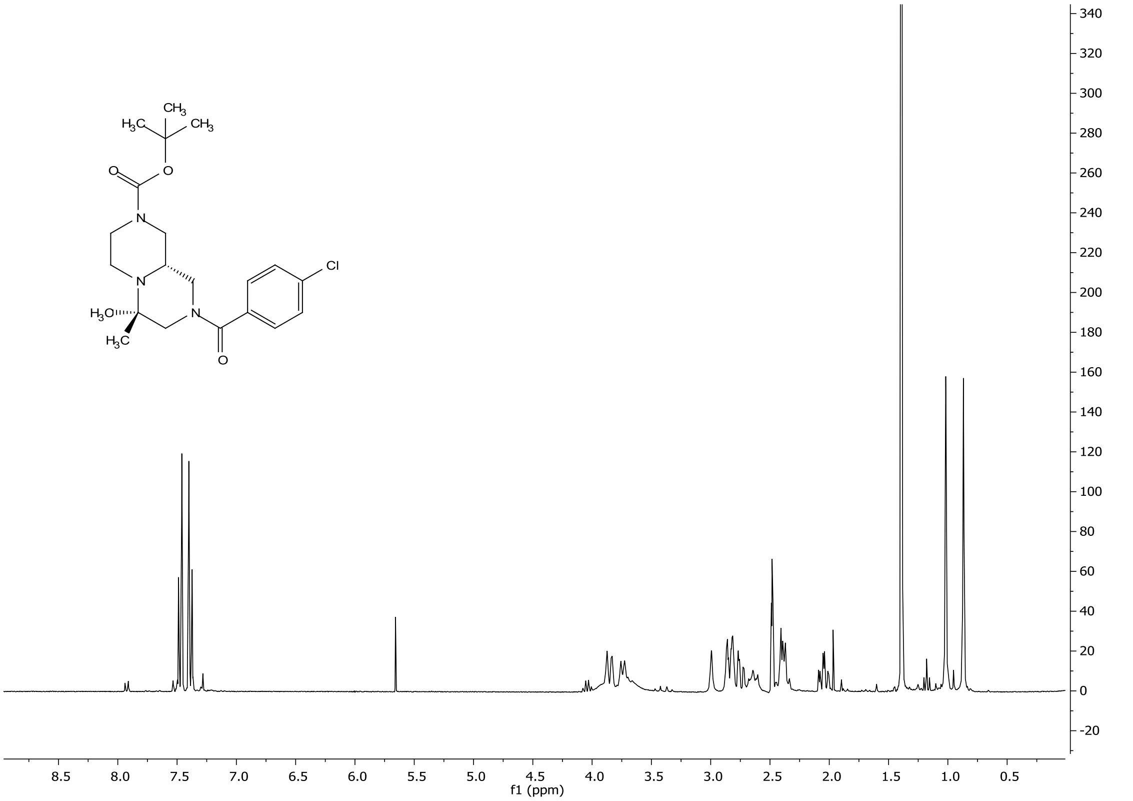

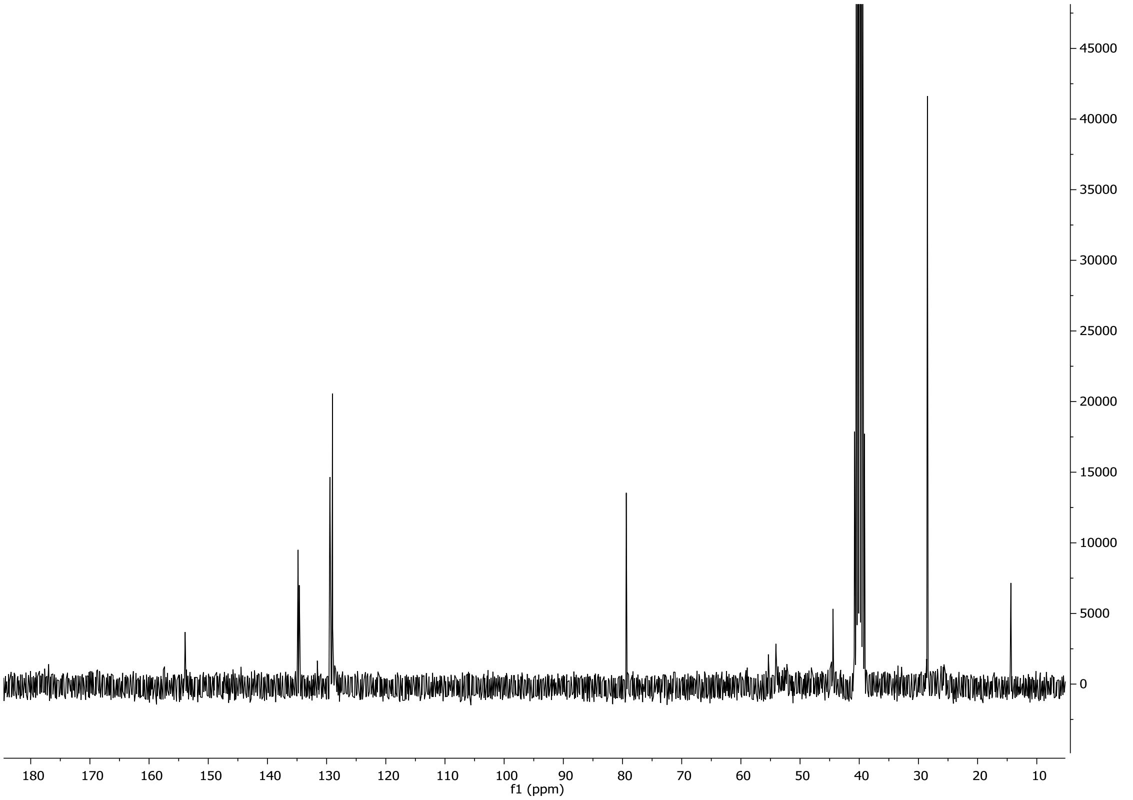

Compound 12{7}

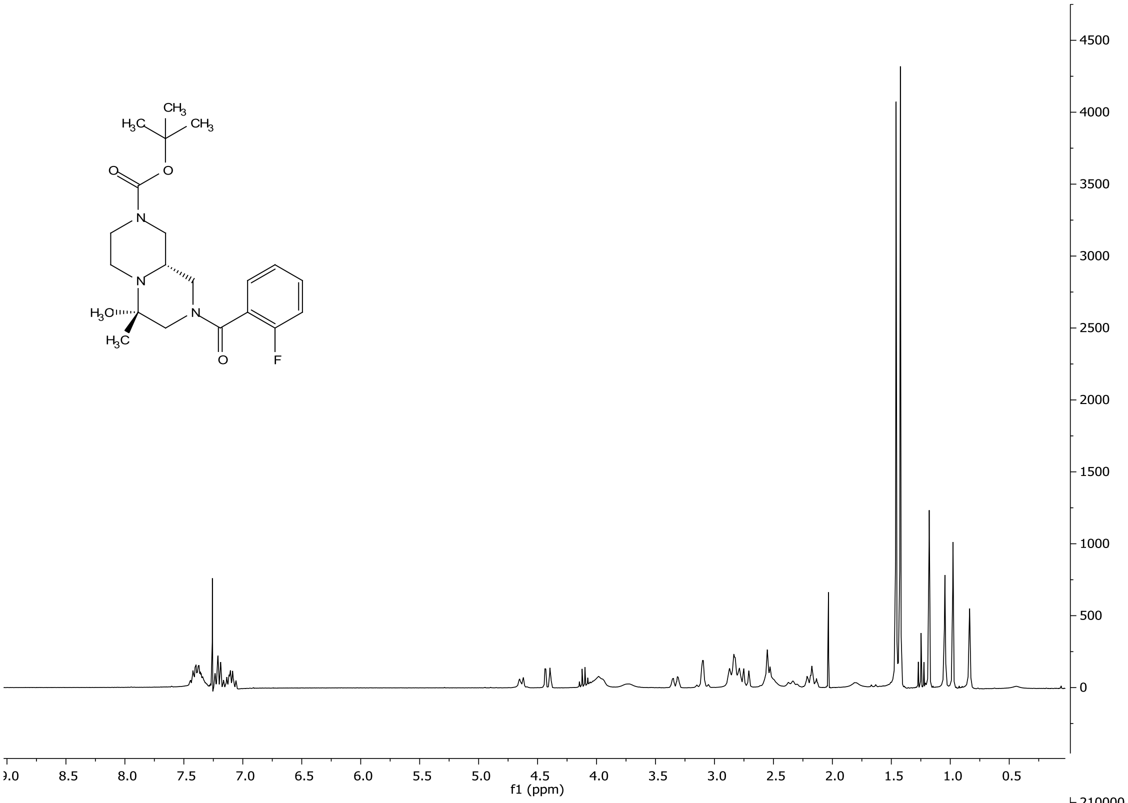

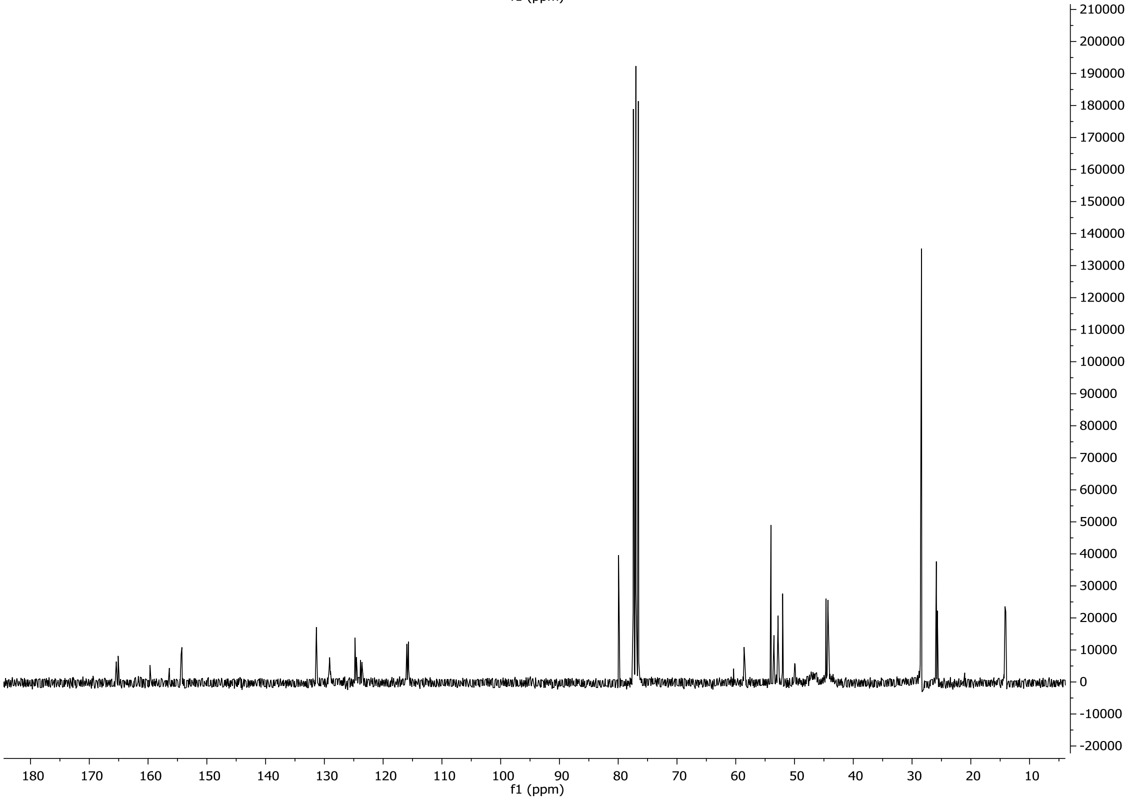

Compound 12{3}

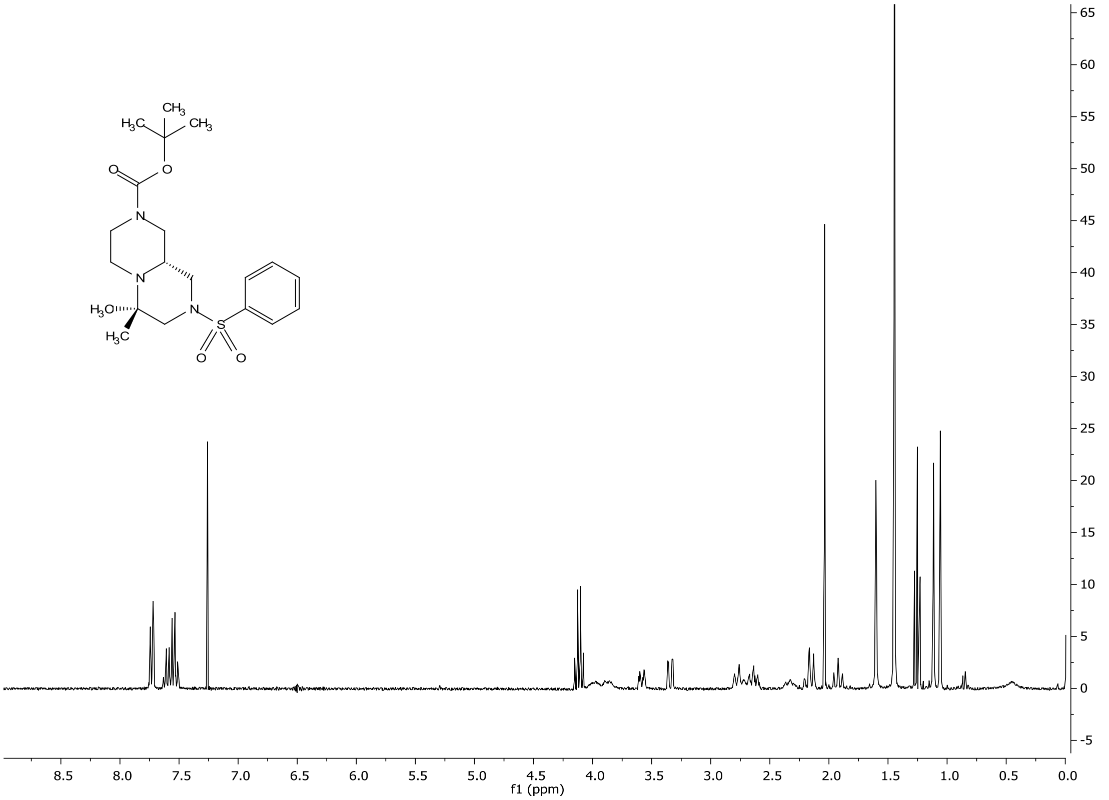

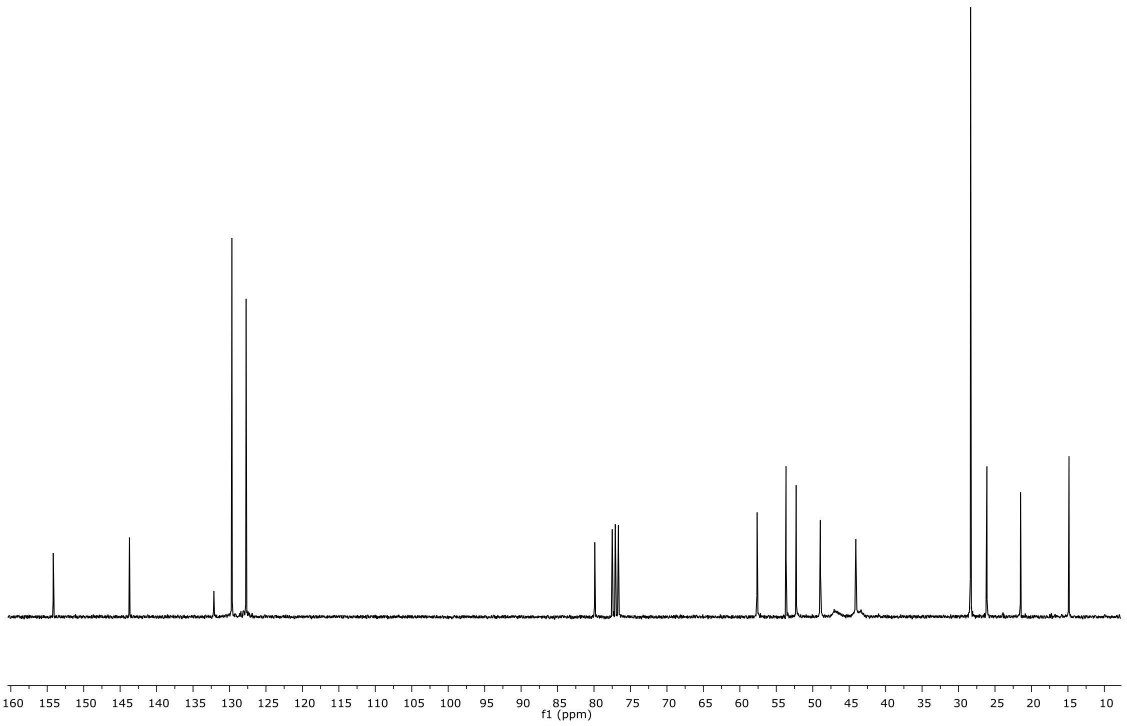

Compound 12{4}

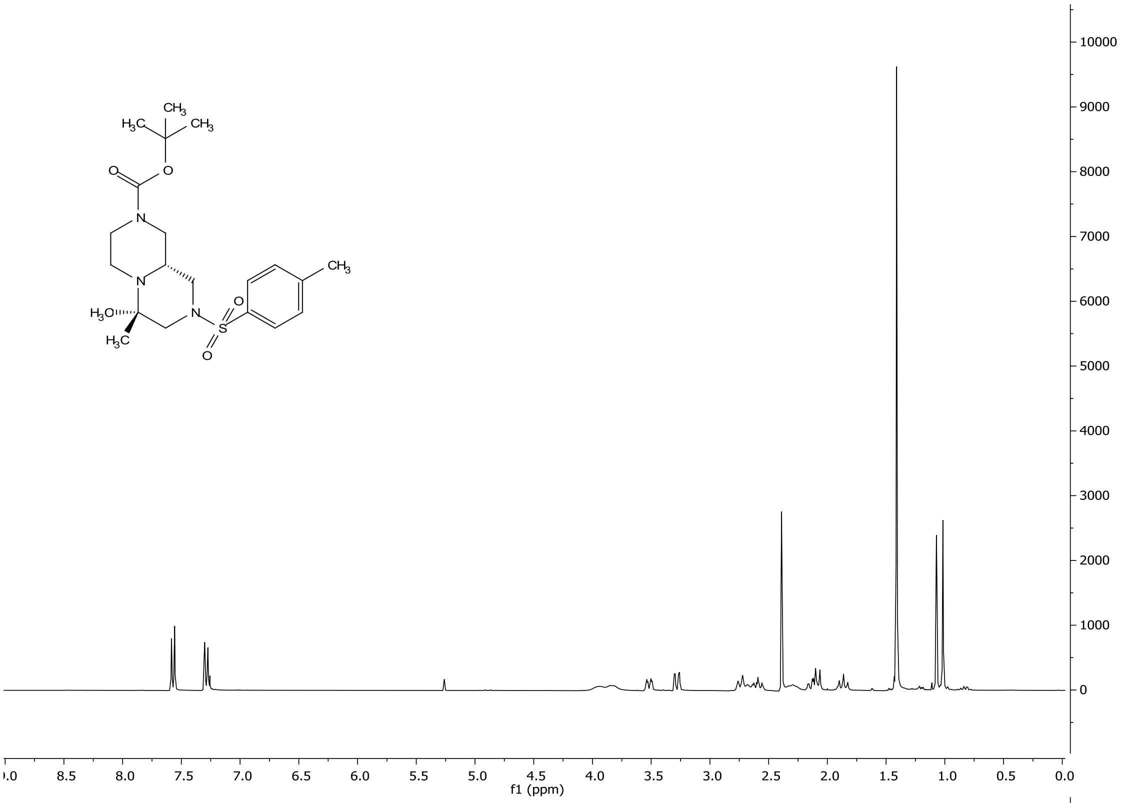

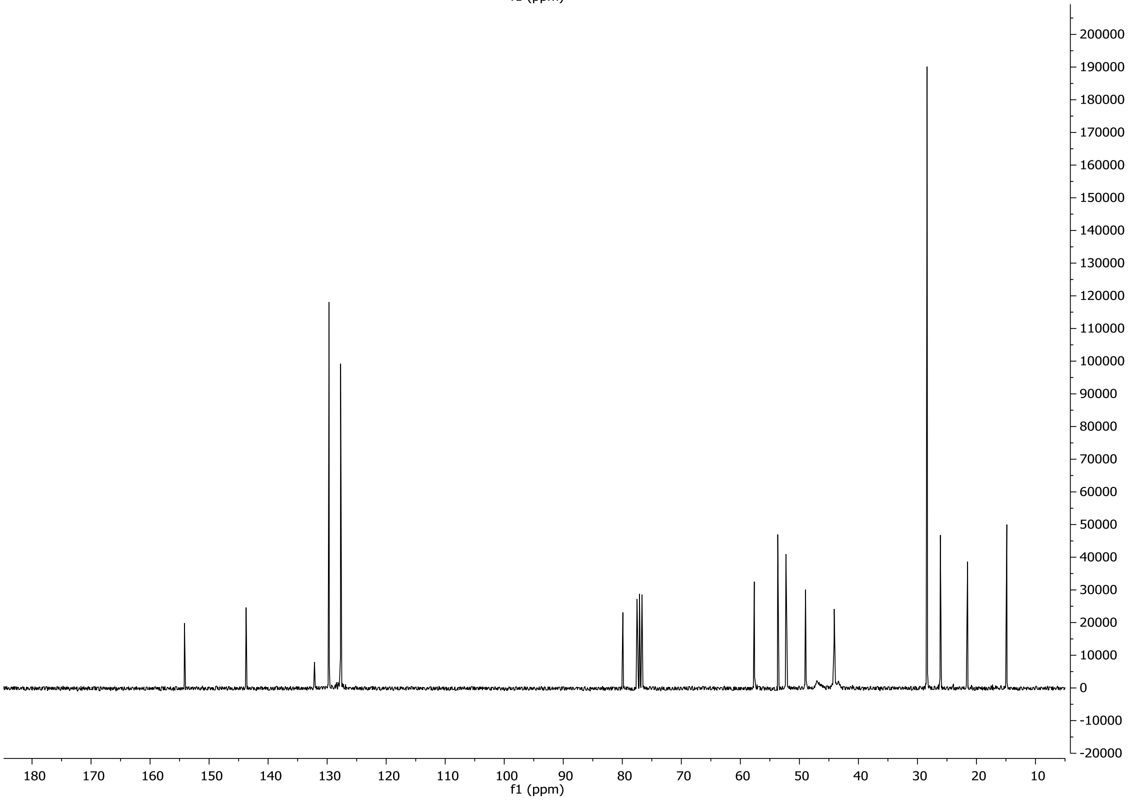

Compound 12{1}

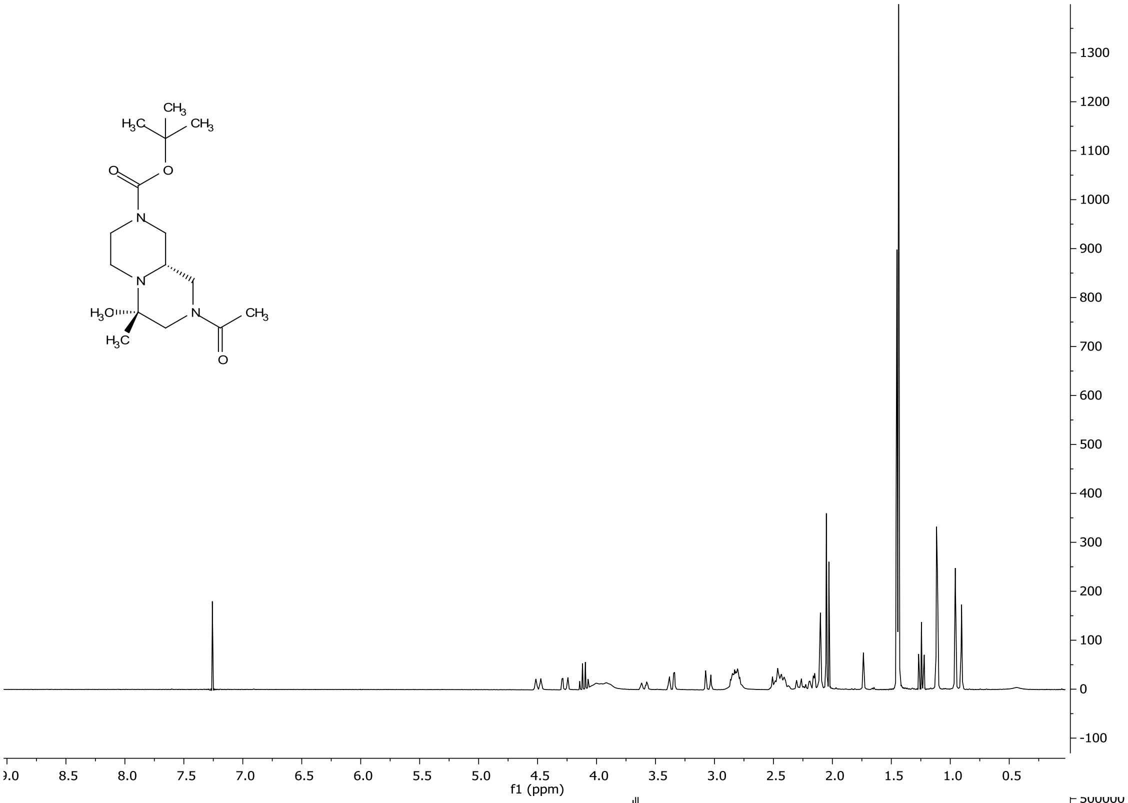

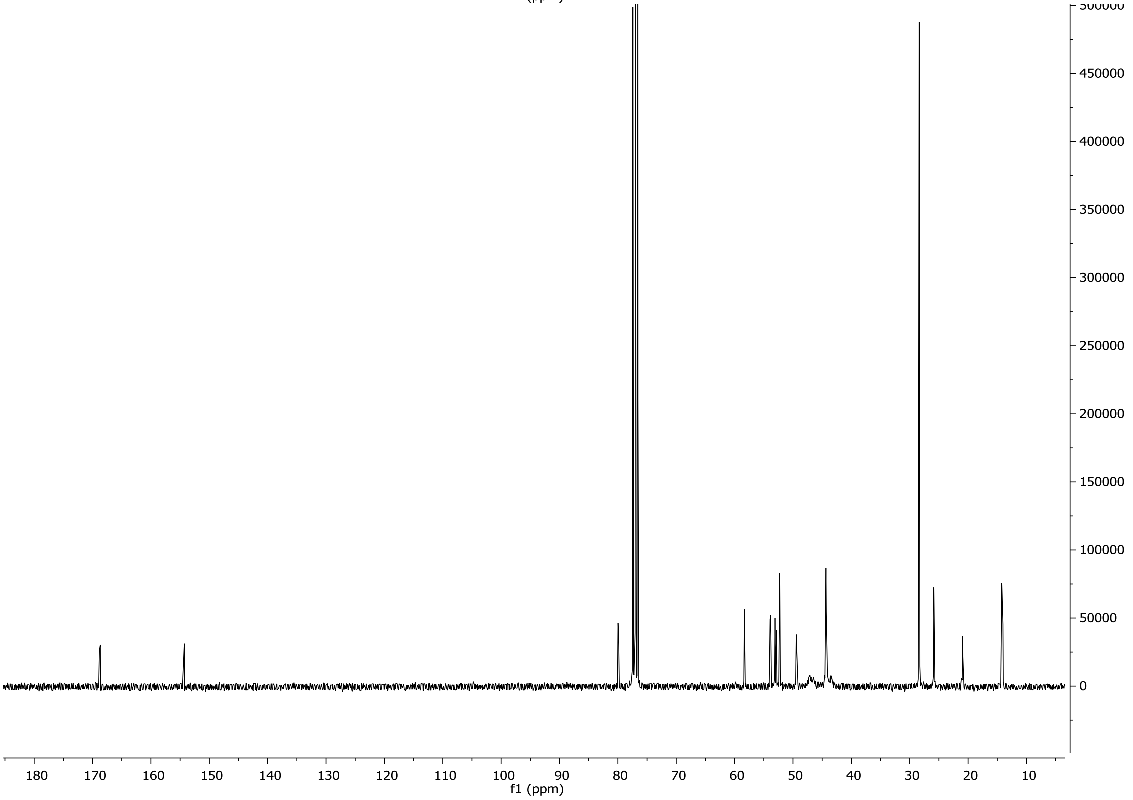

Compound 12{9}

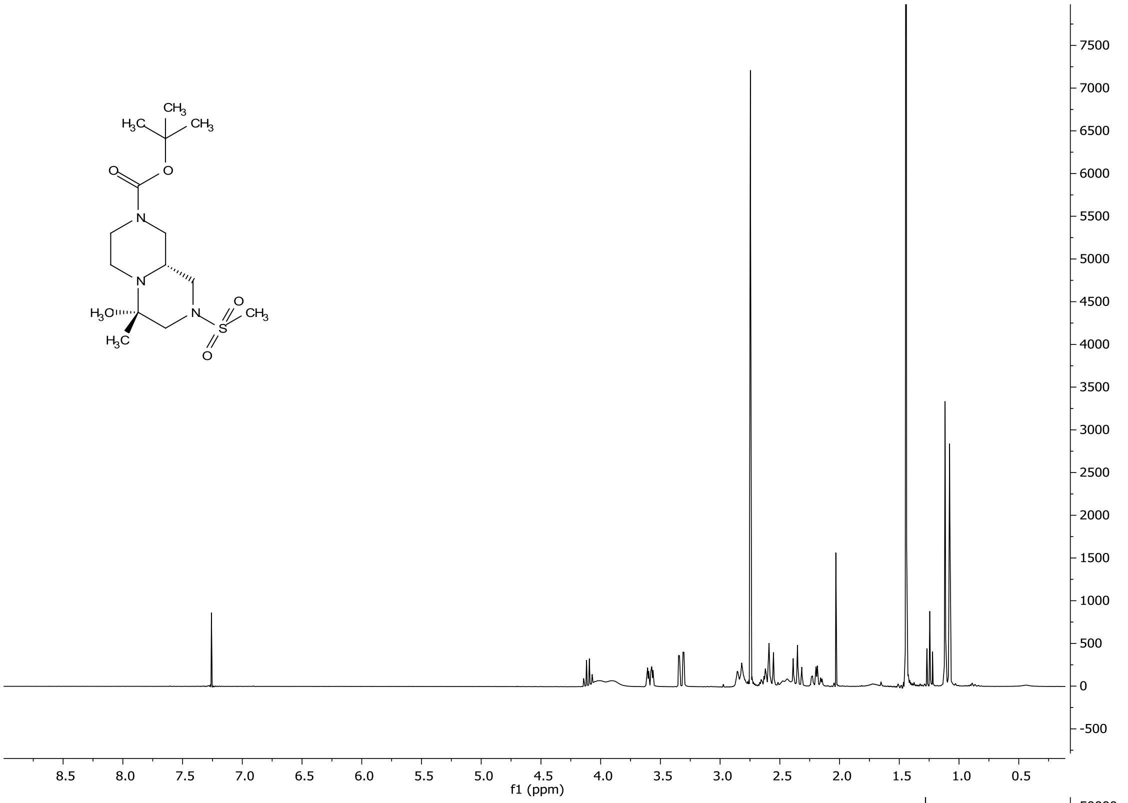

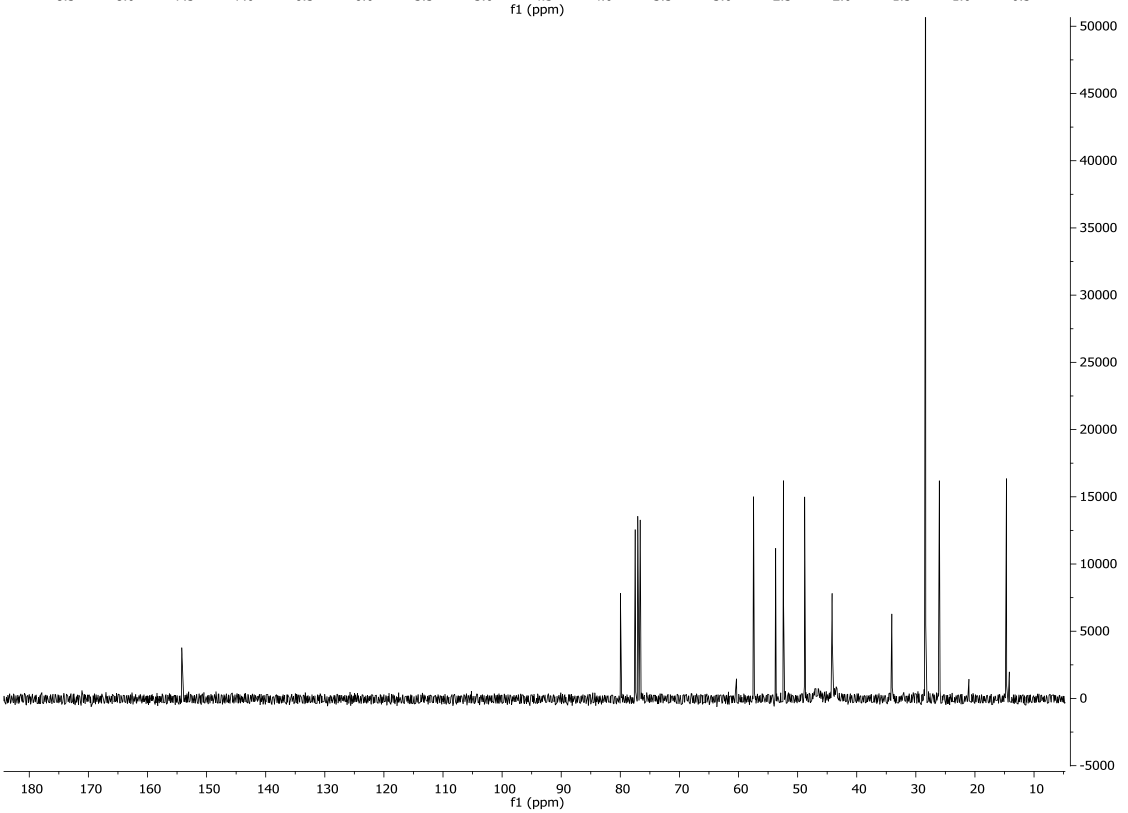

Compound 12{5}

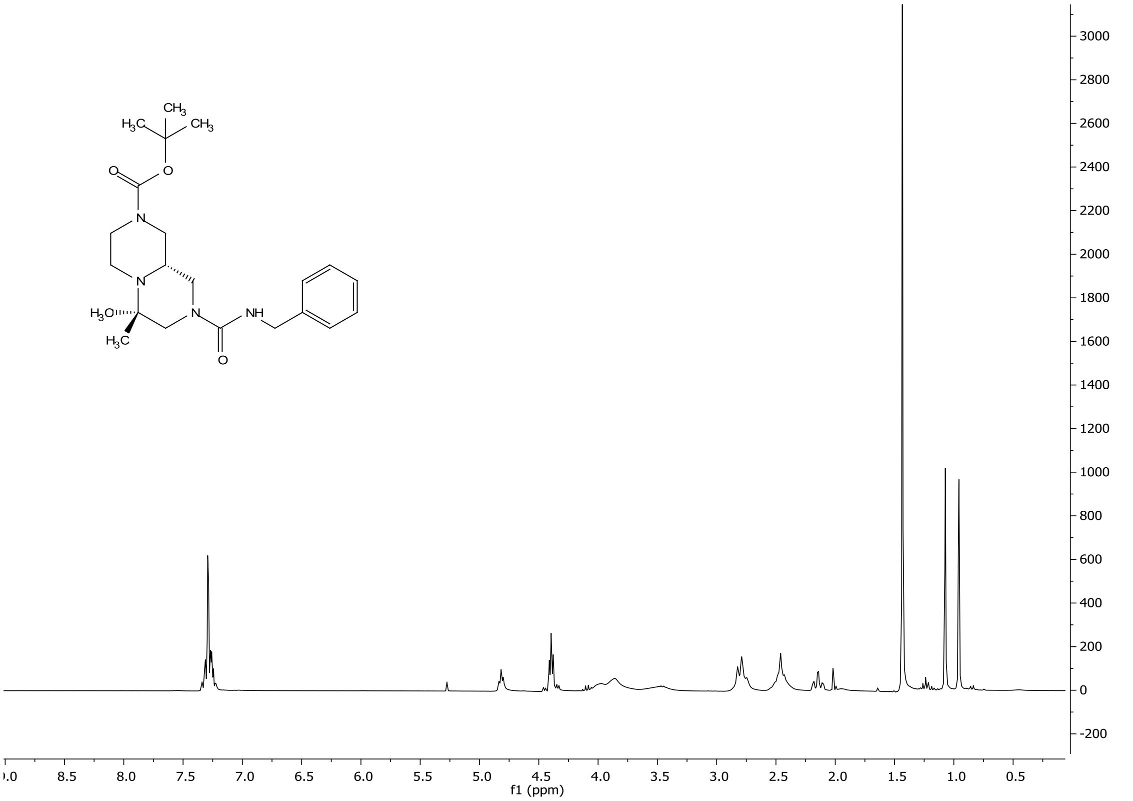

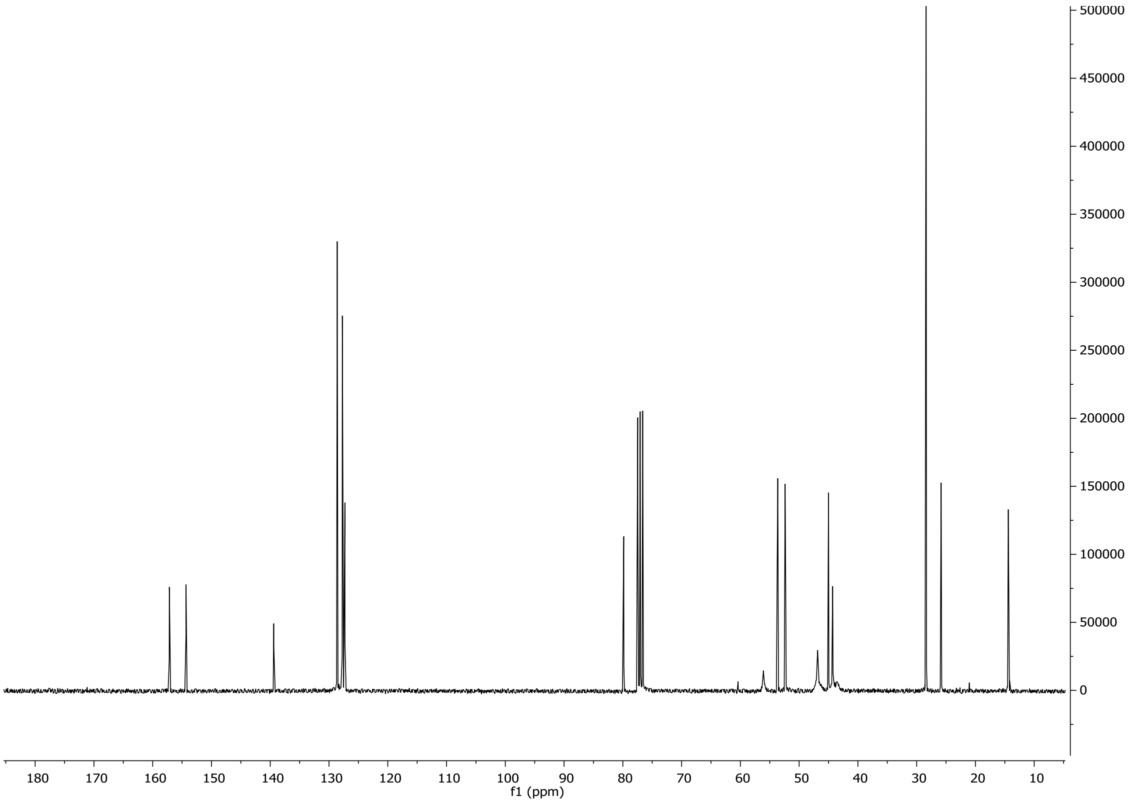

Compound 12{2}

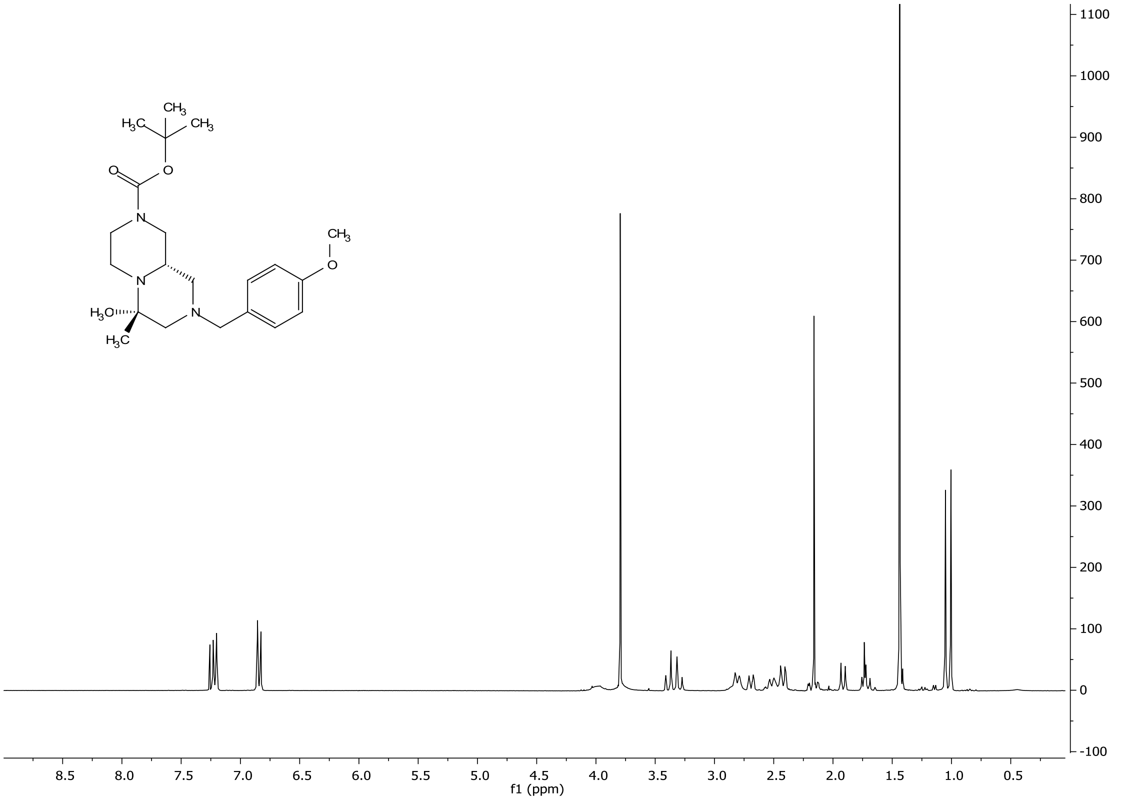

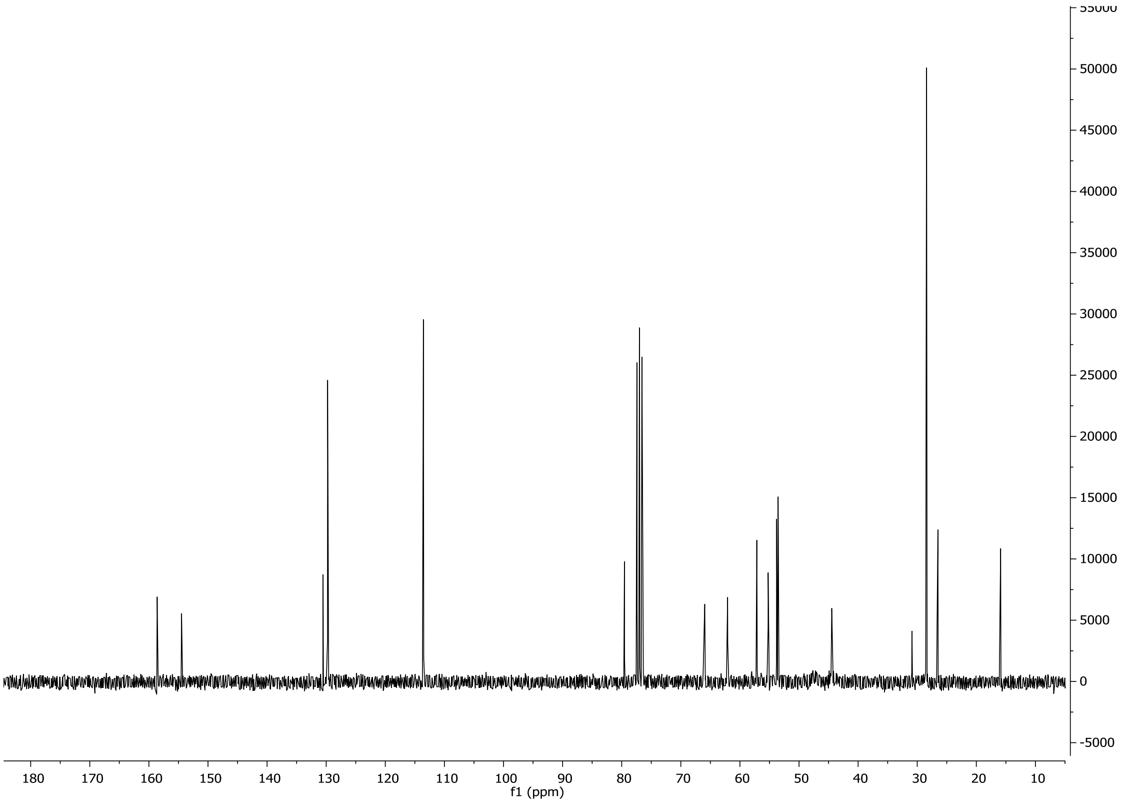

Compound 12{8}

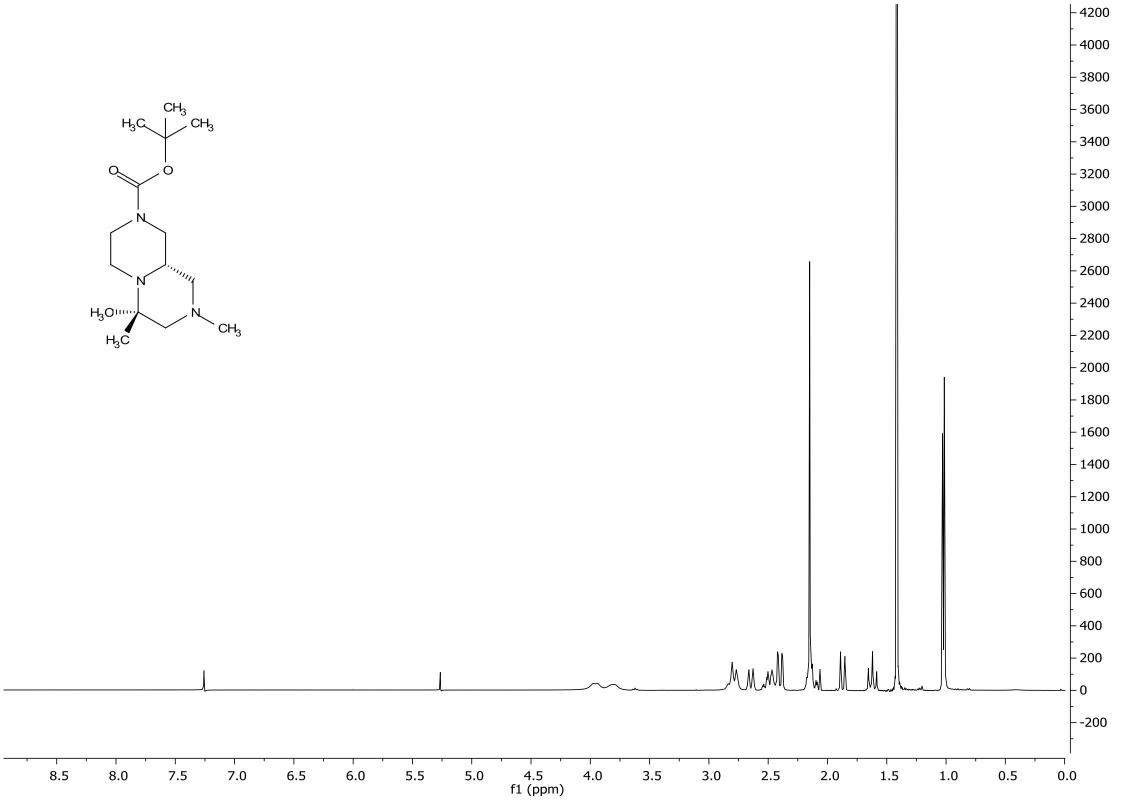

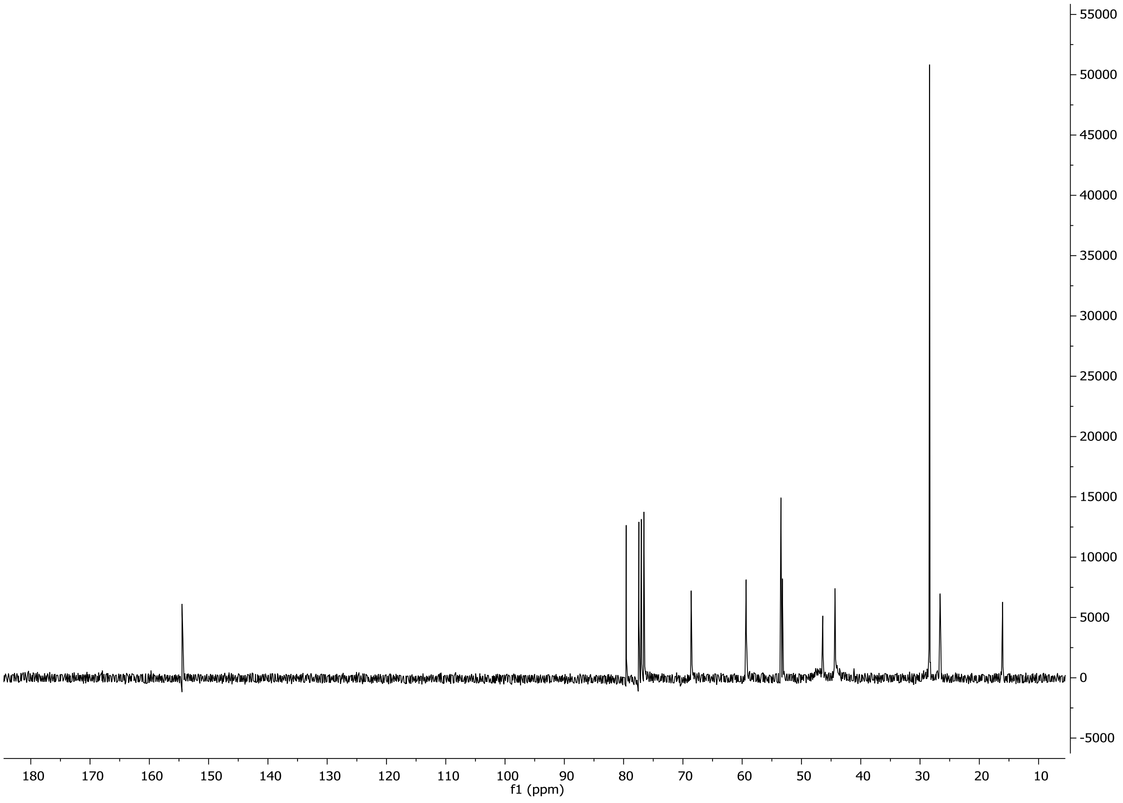

Compound 14{3}

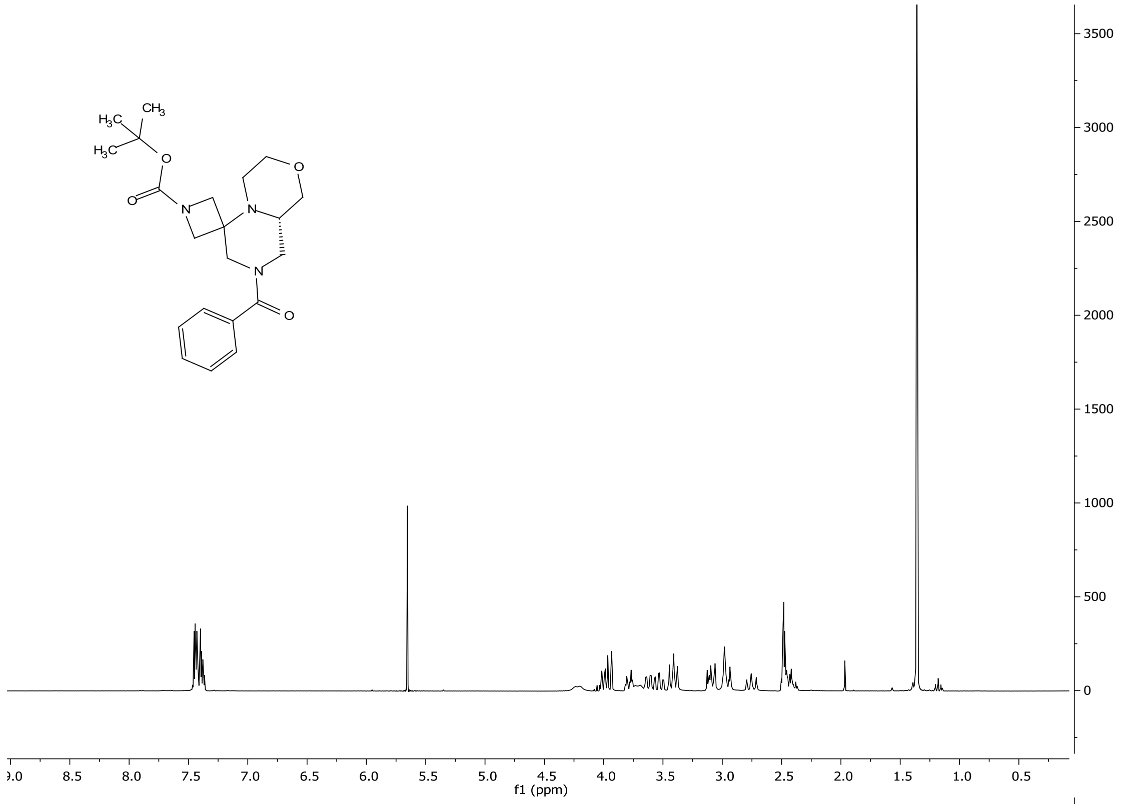

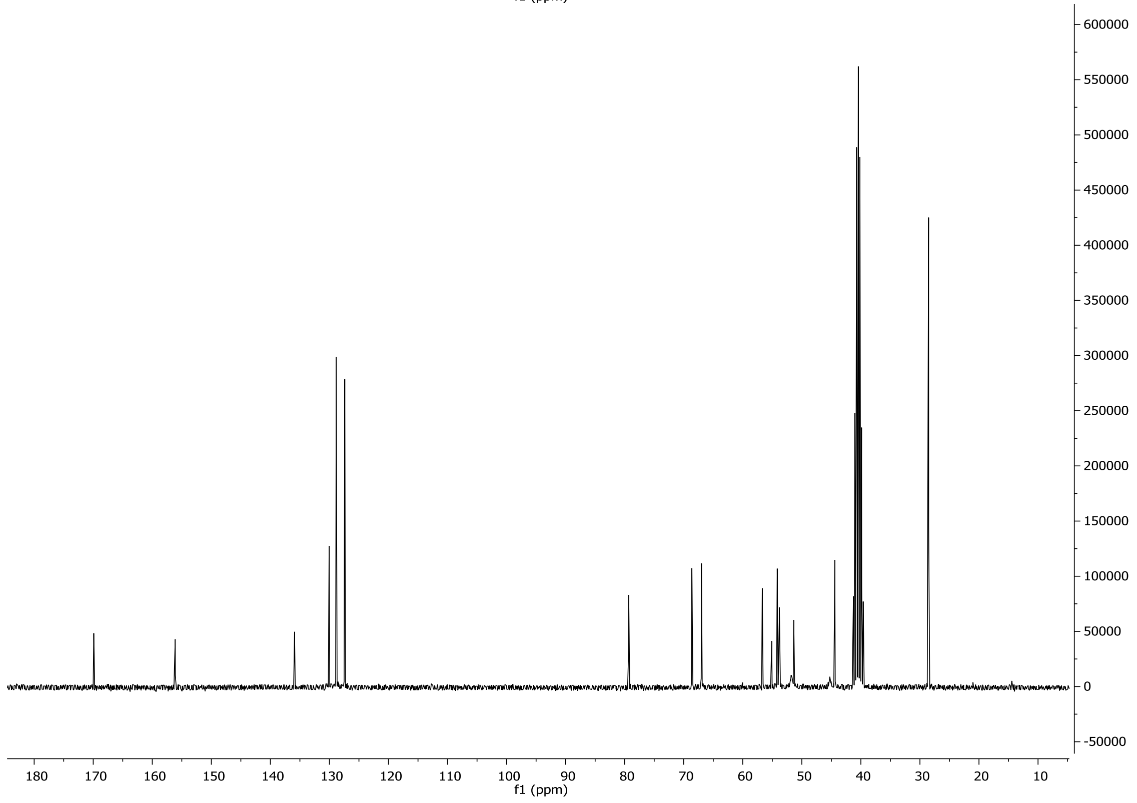

Compound 14{1}

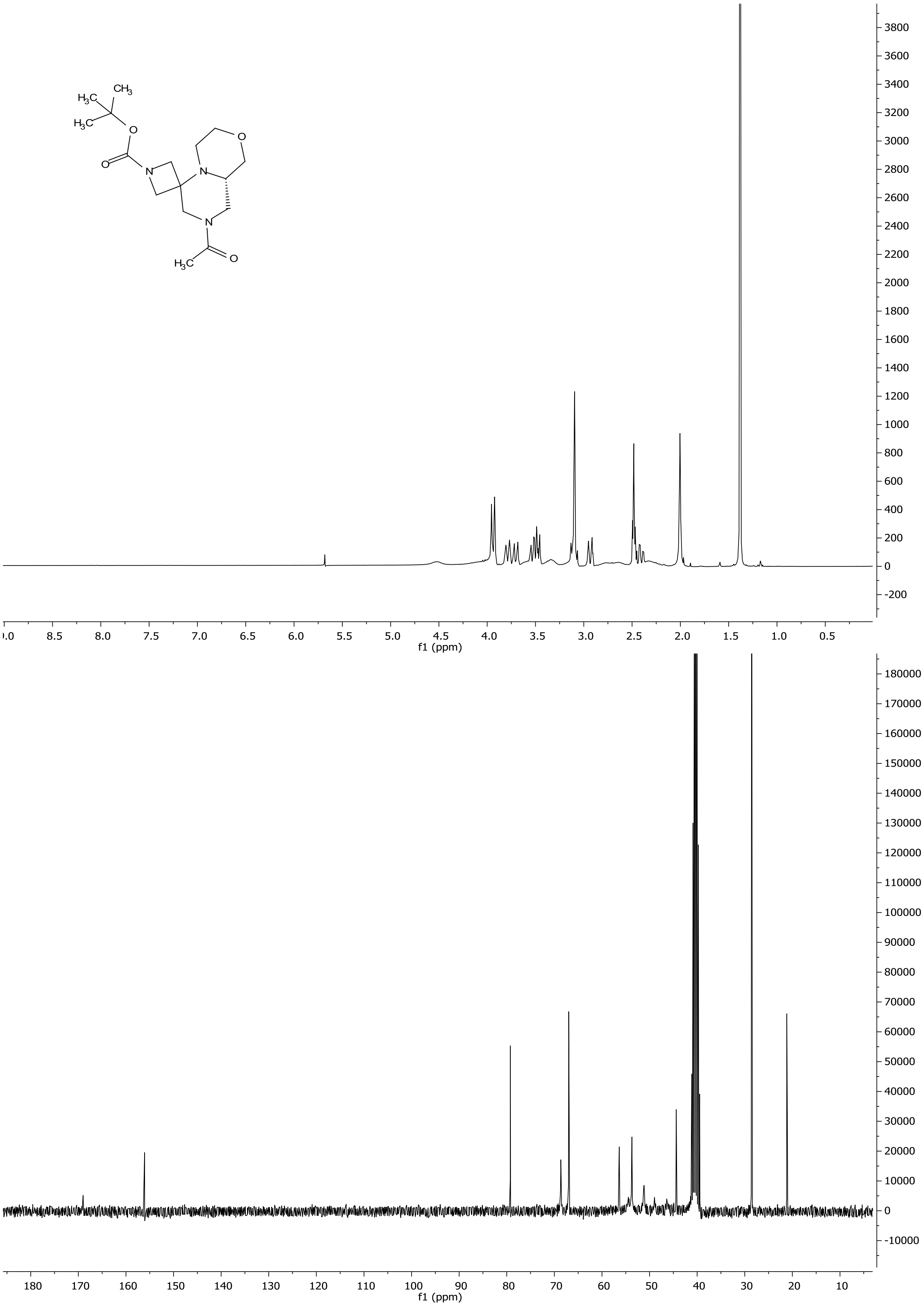

Compound 14{7}

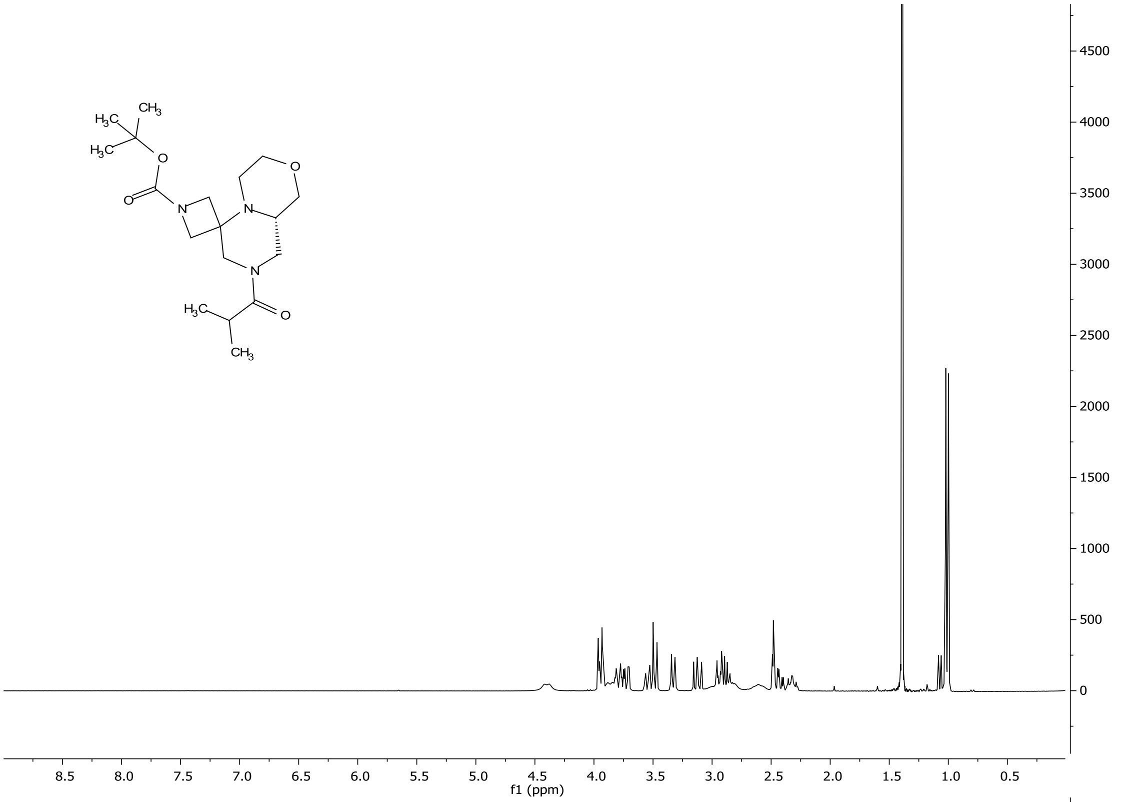

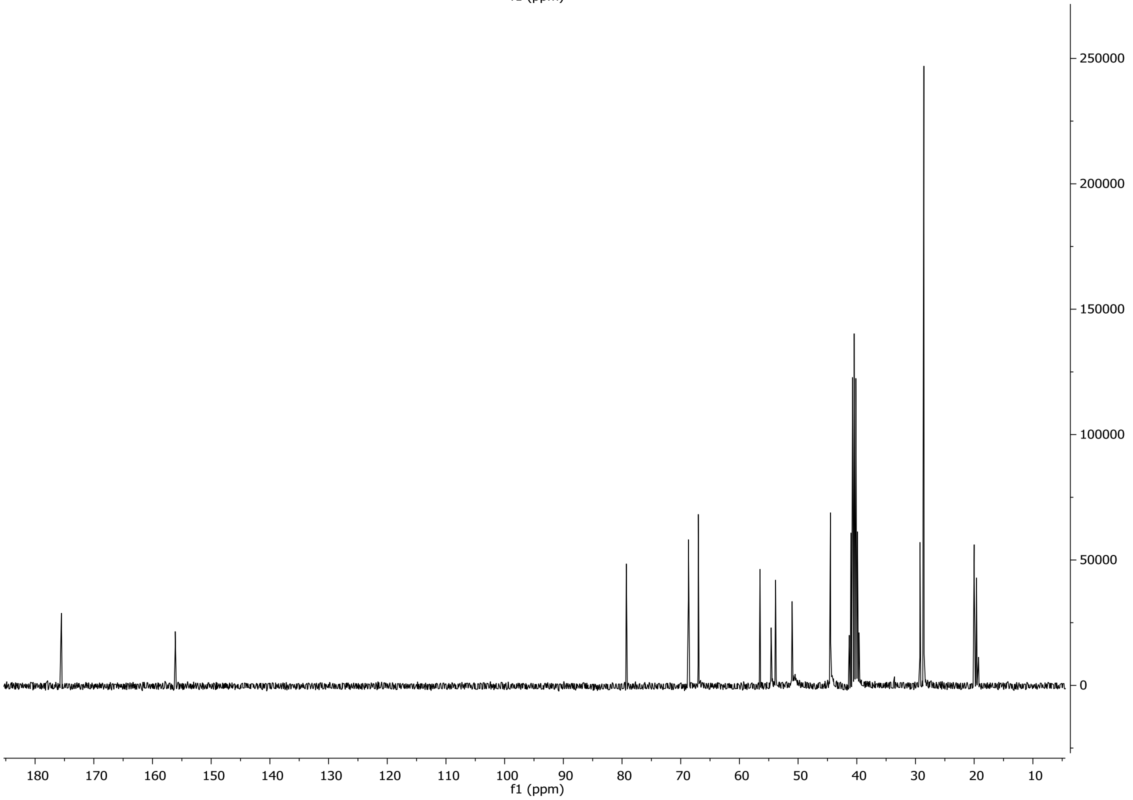

Compound 14{4}

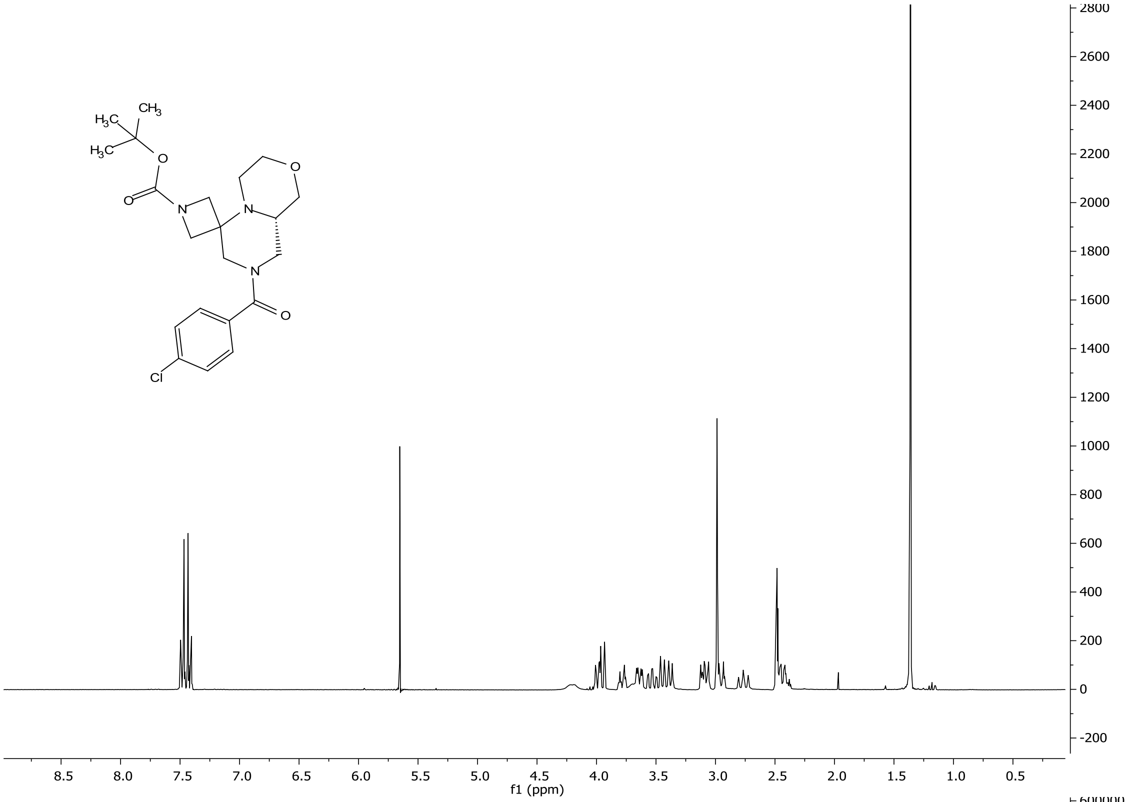

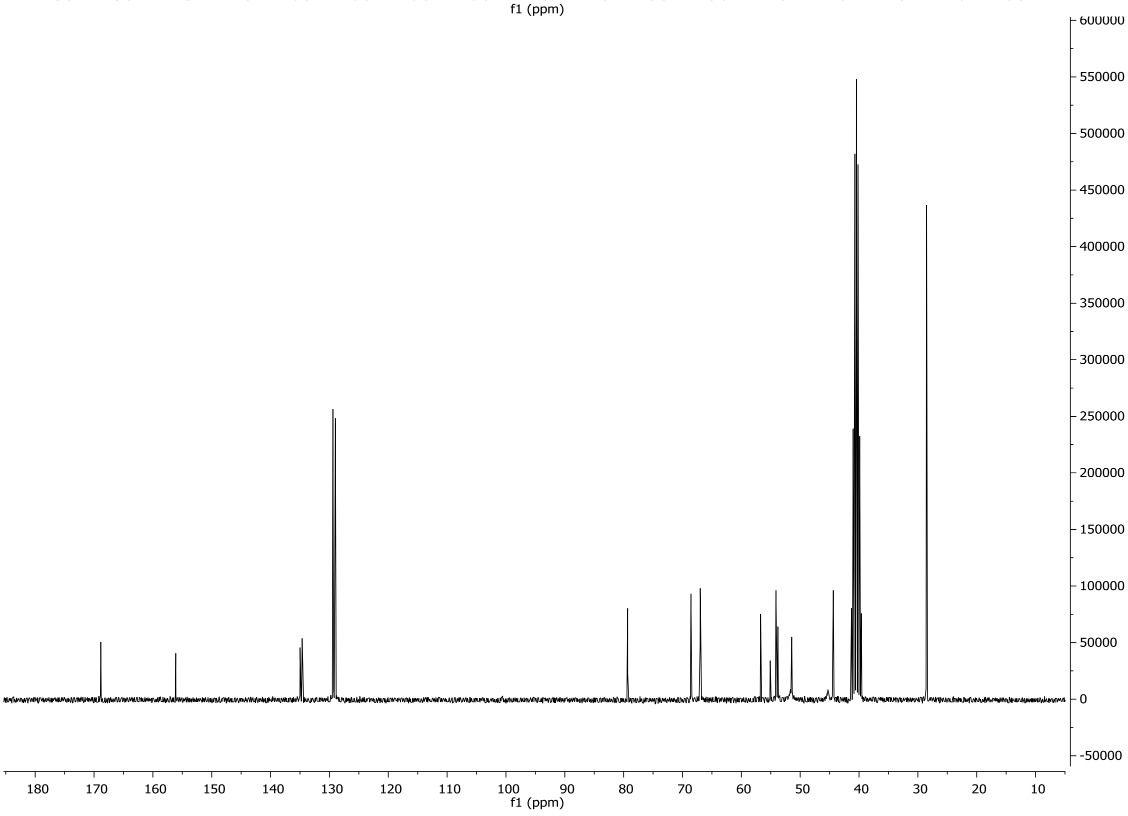

Compound 14{9}

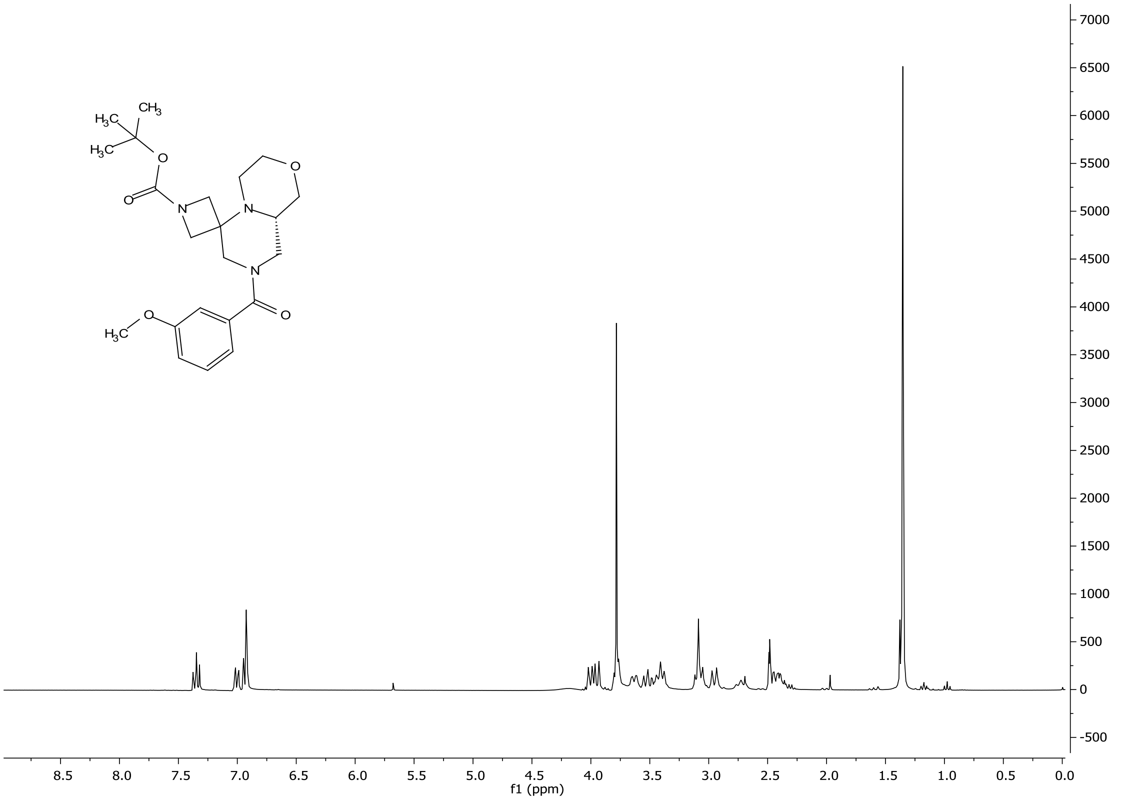

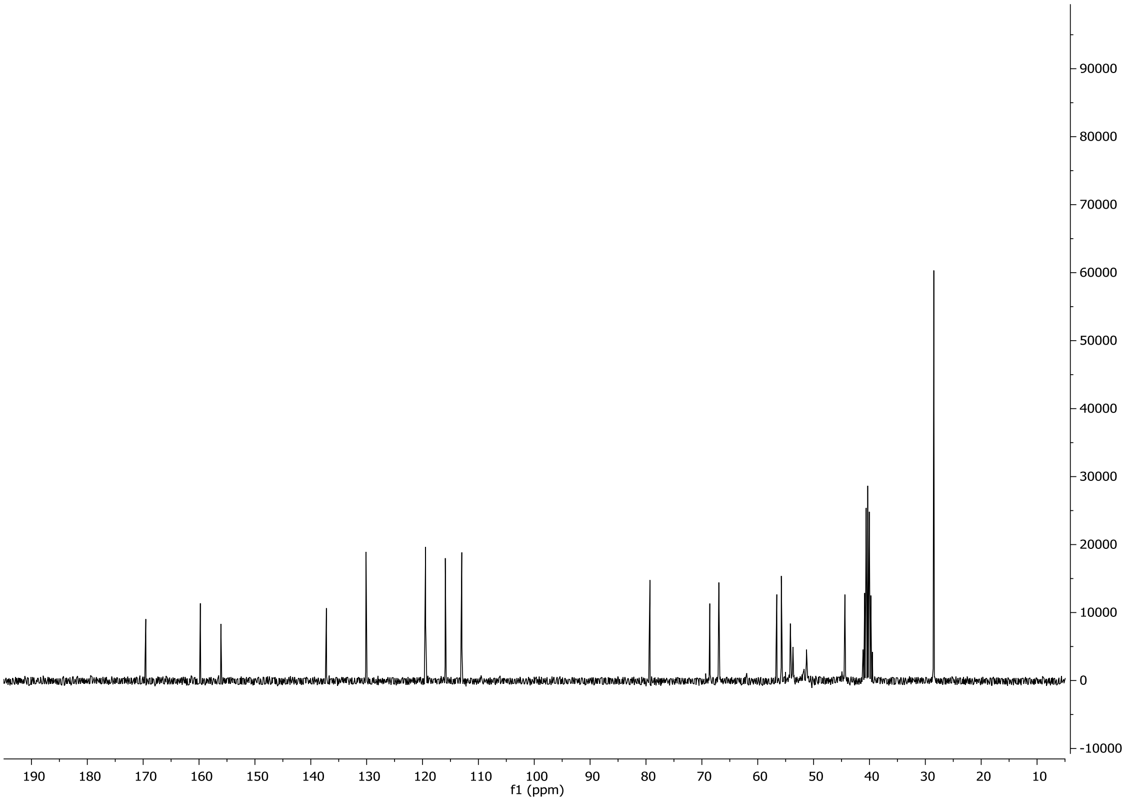

Compound 14{11}

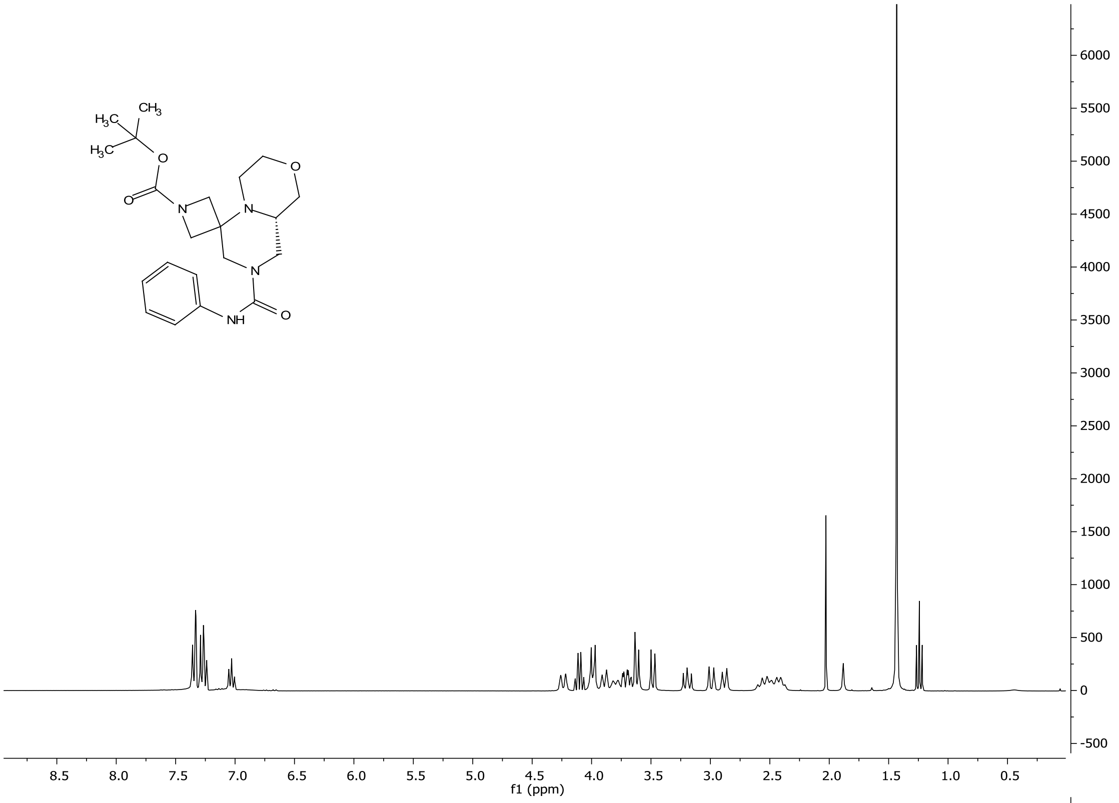

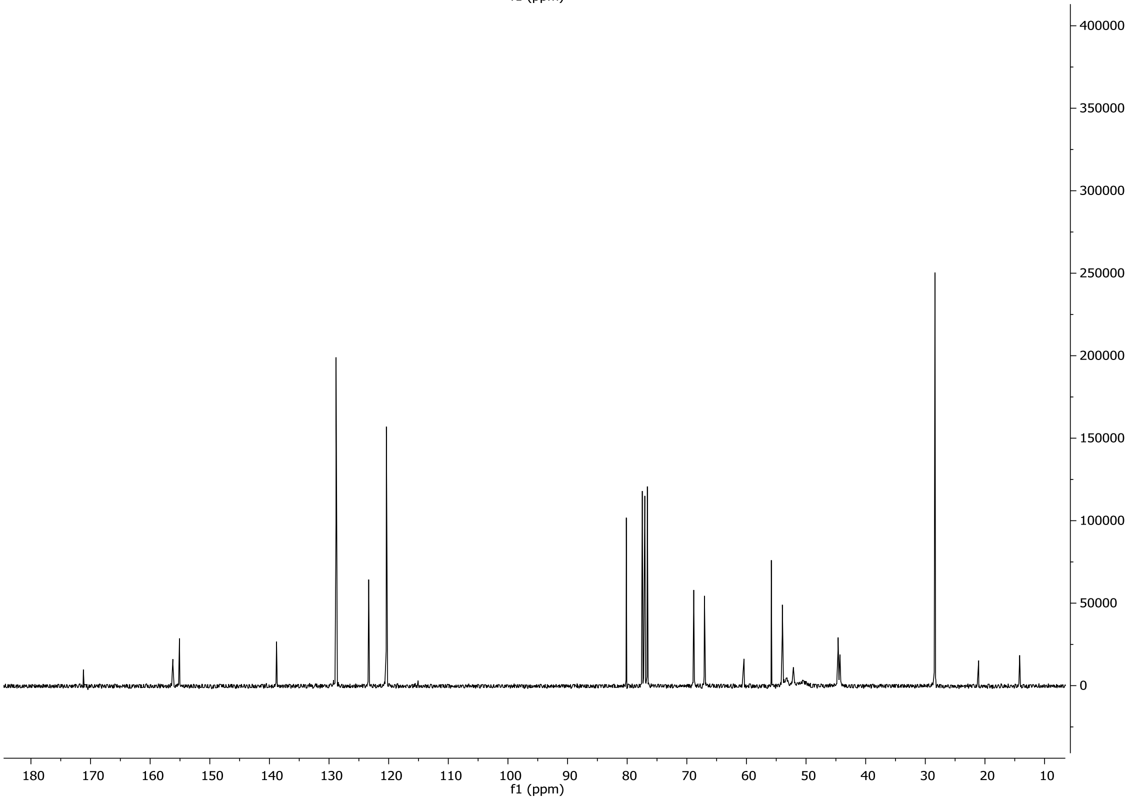

Compound 14{5}

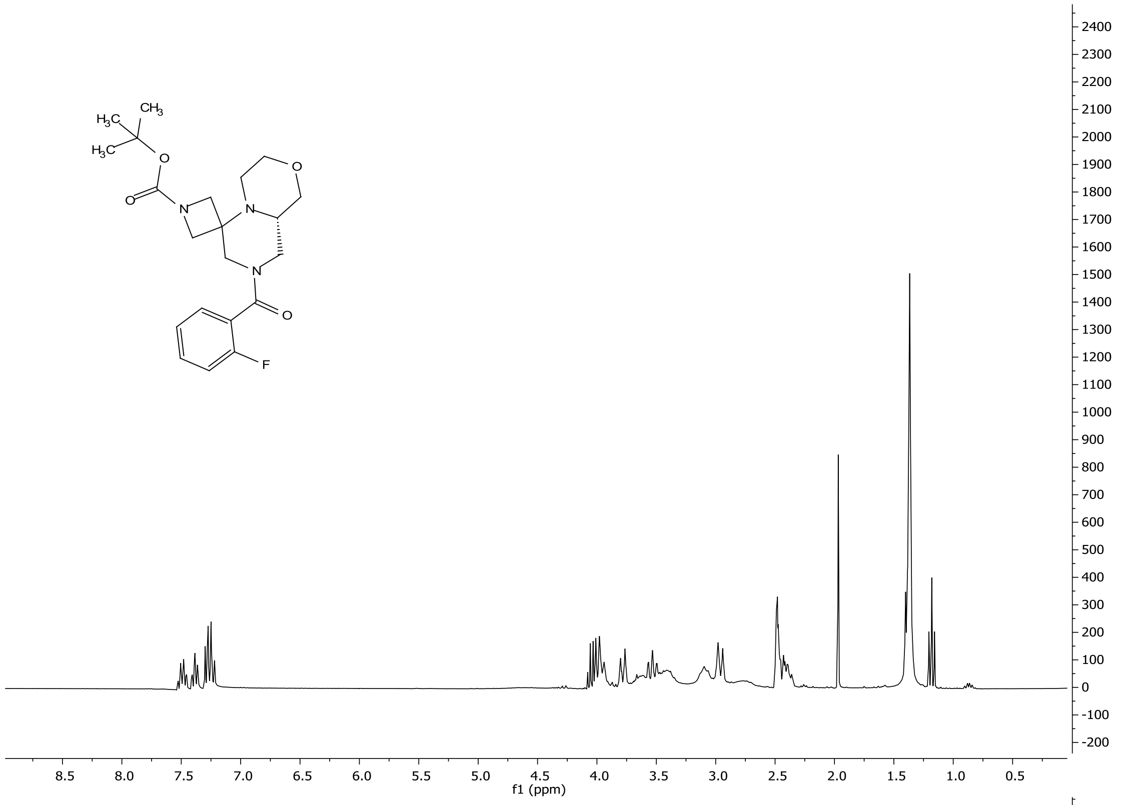

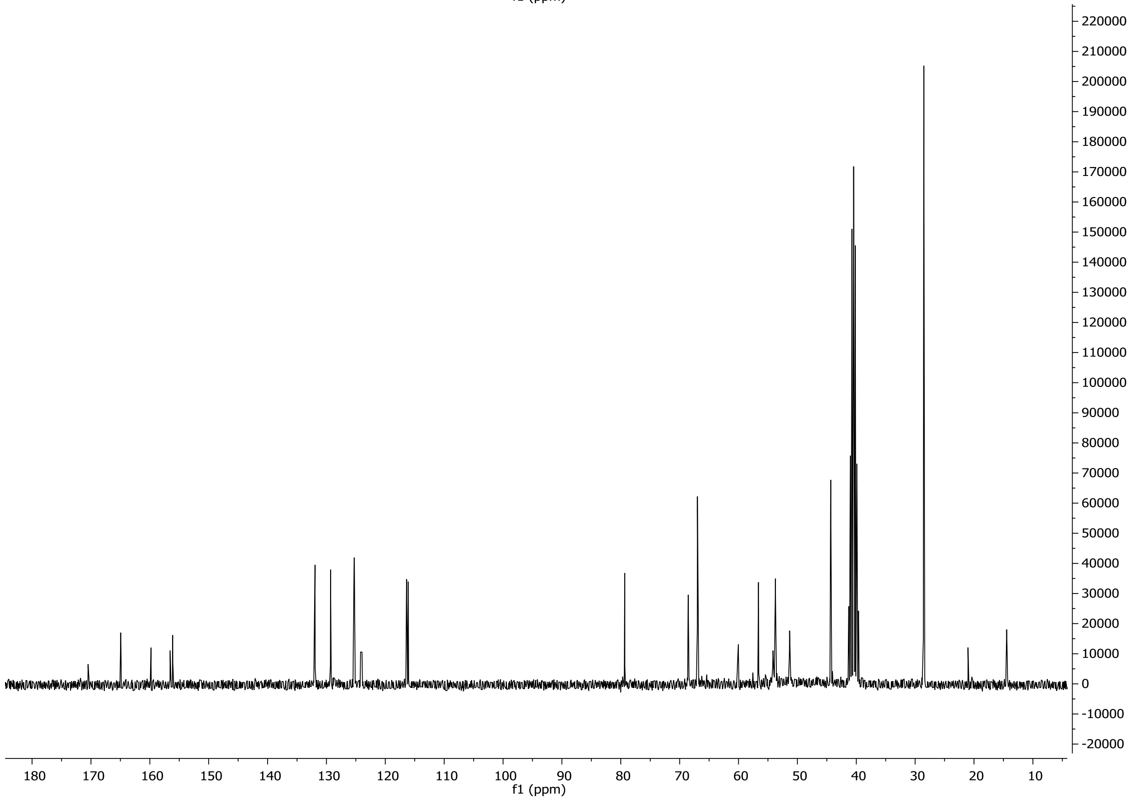

Compound 14{10}

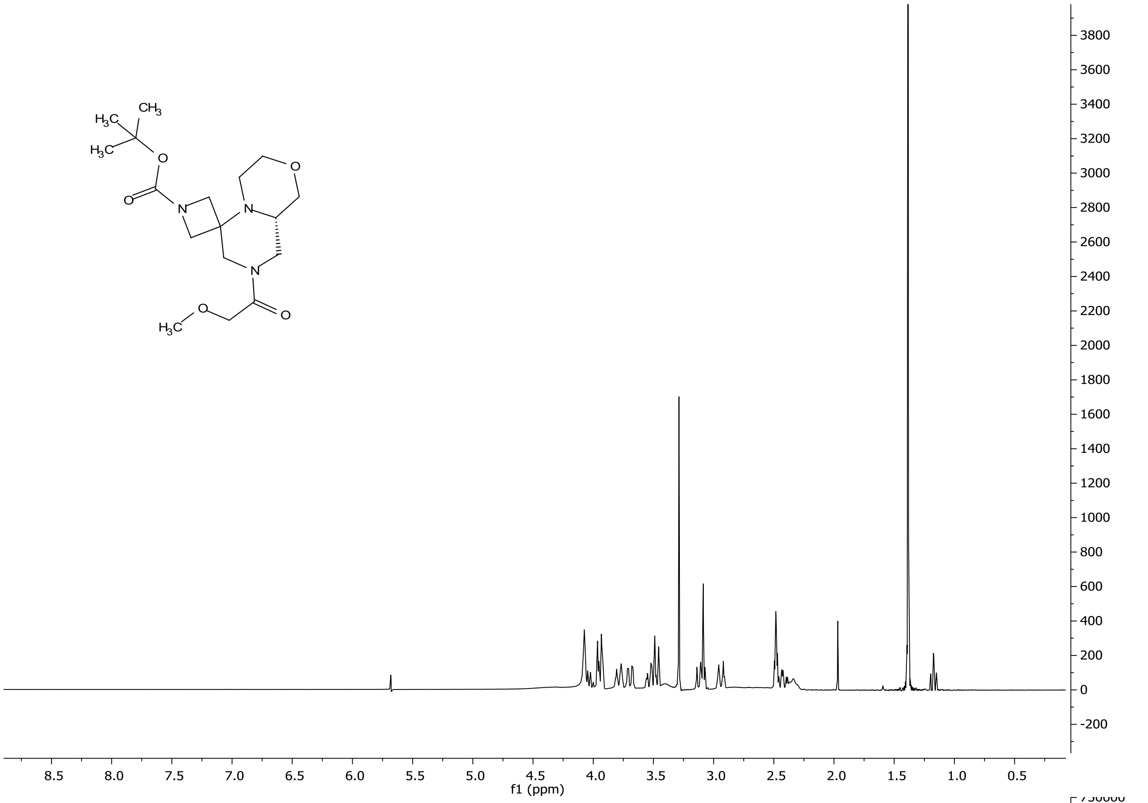

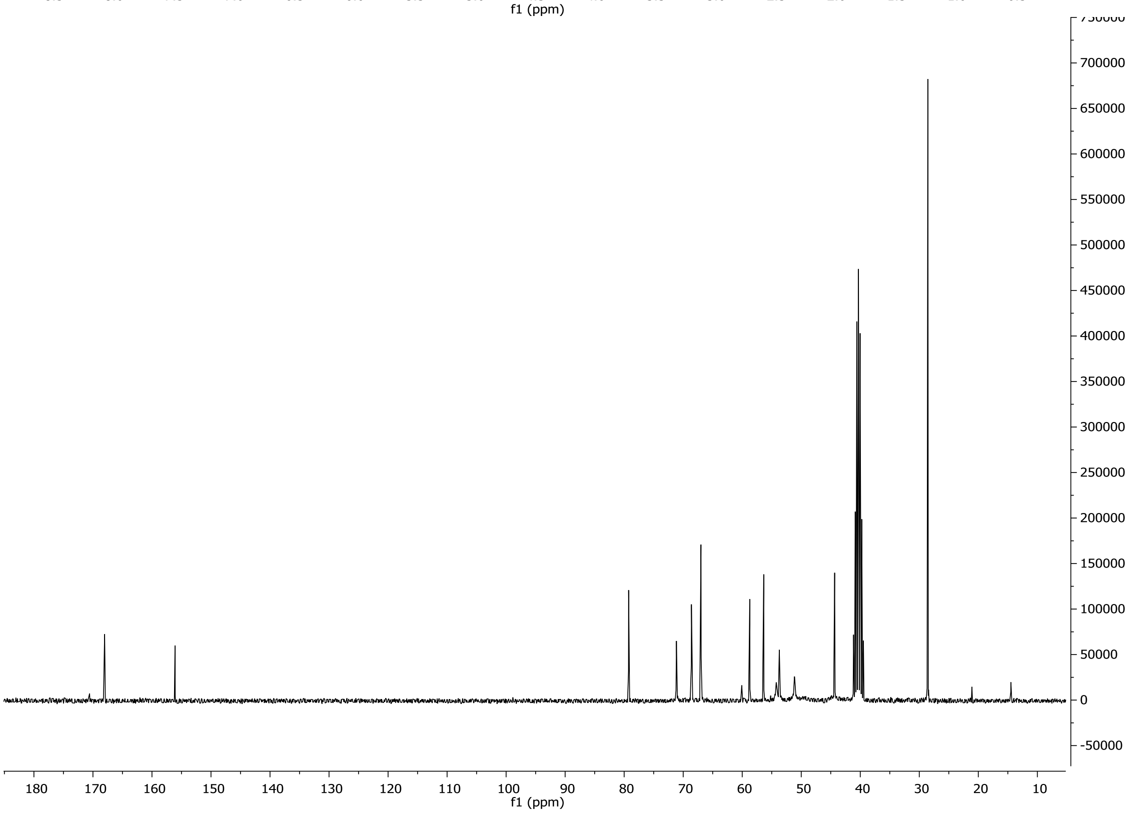

Compound 14{8}

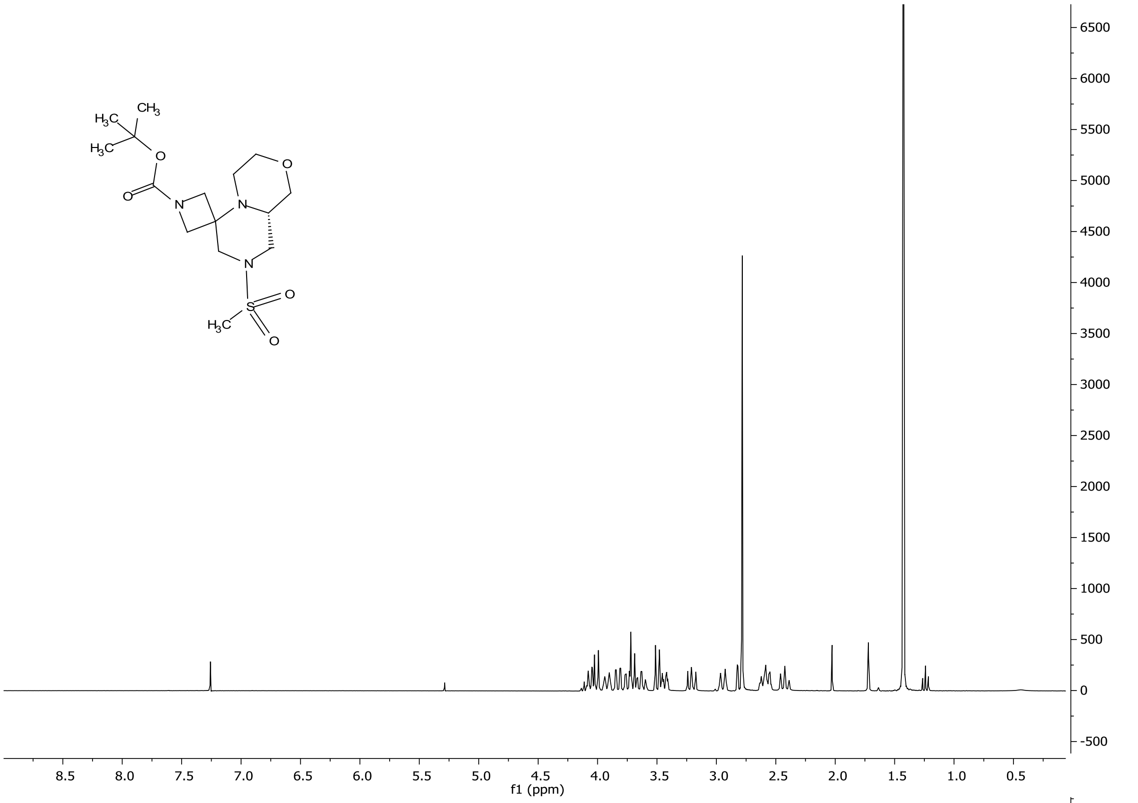

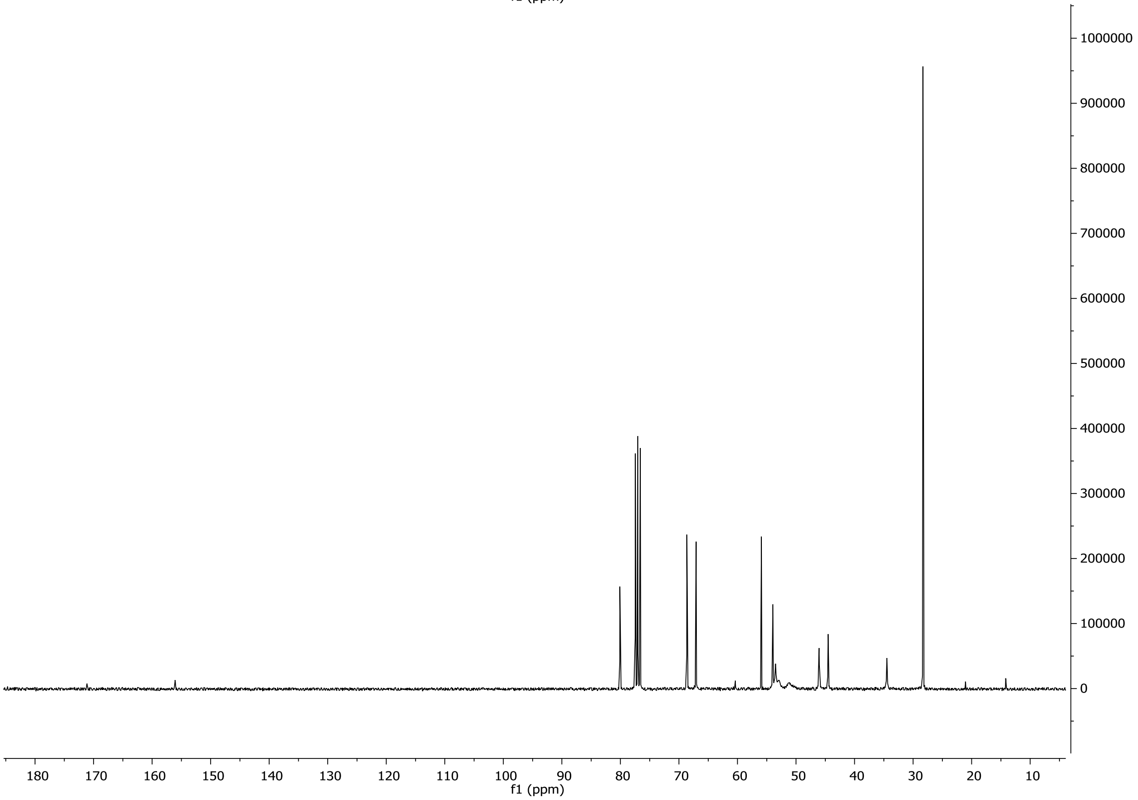

Compound 14{6}

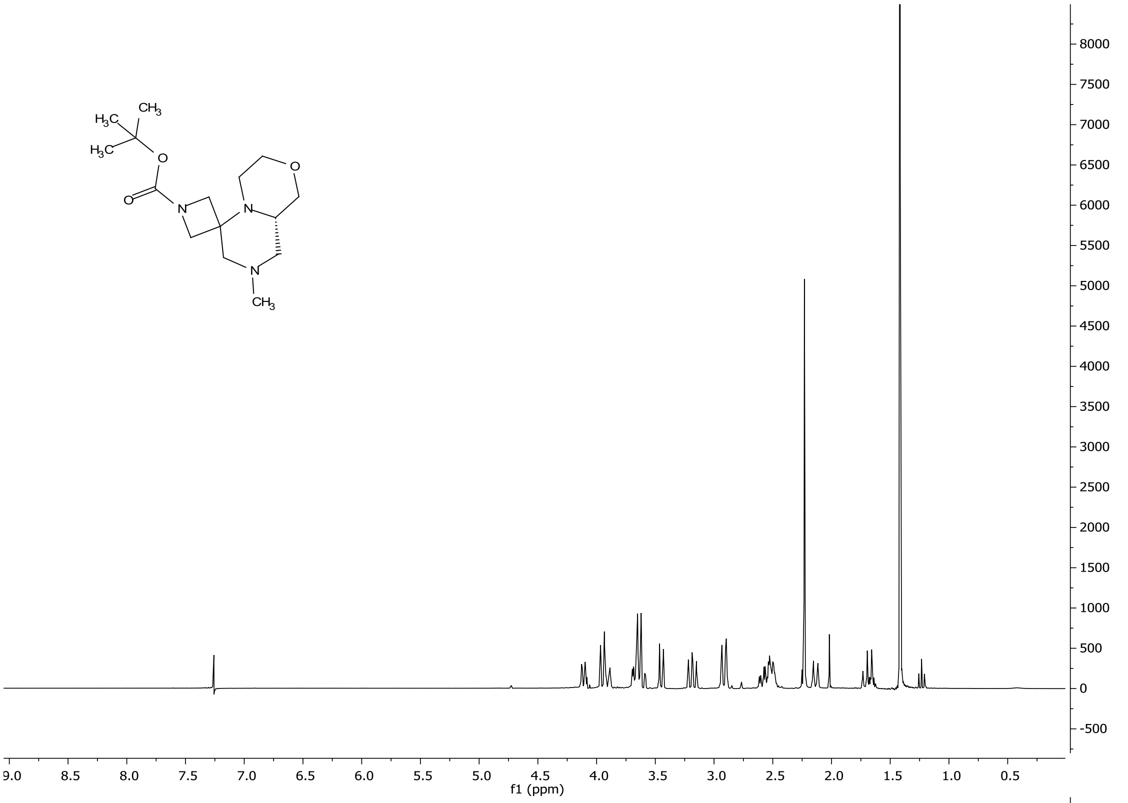

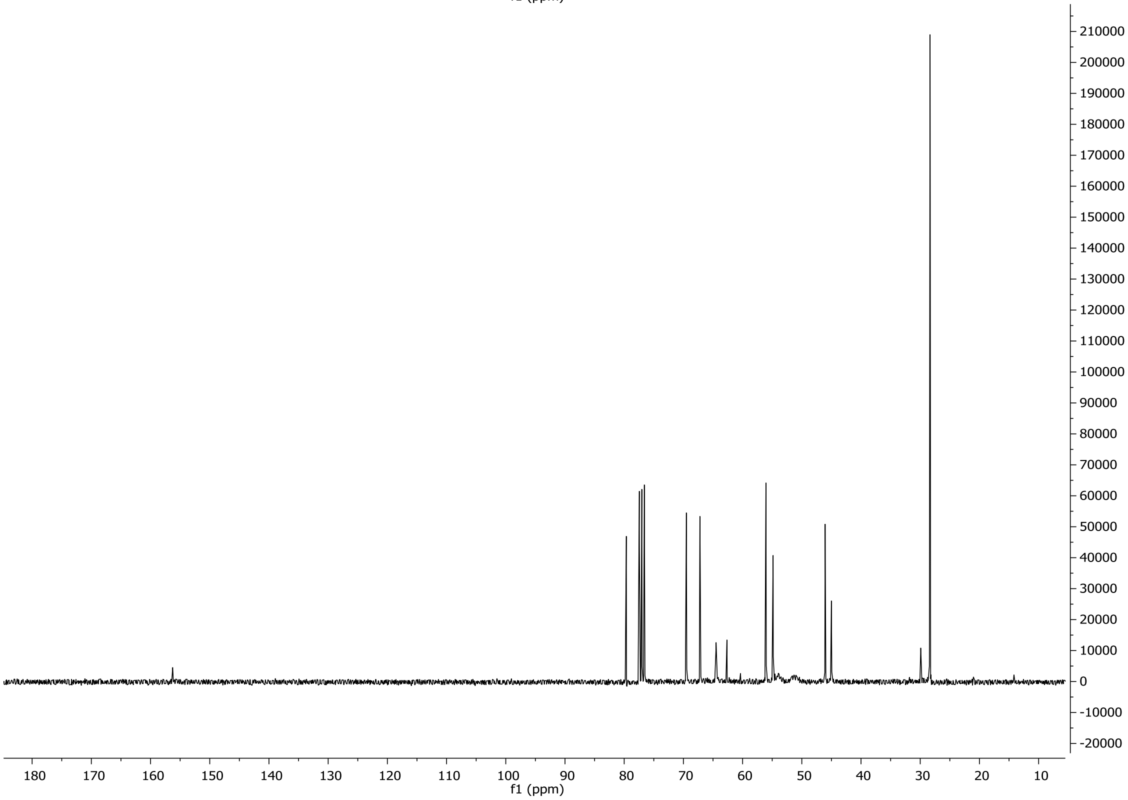

Compound 14{2}

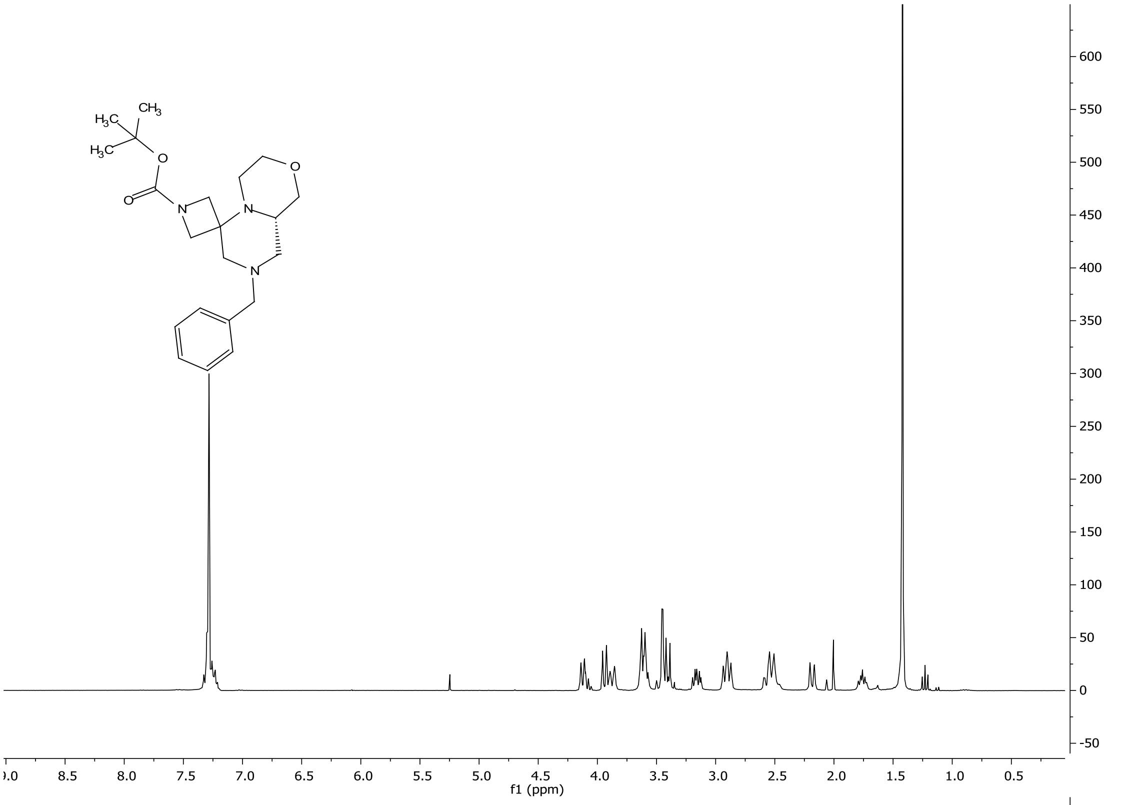

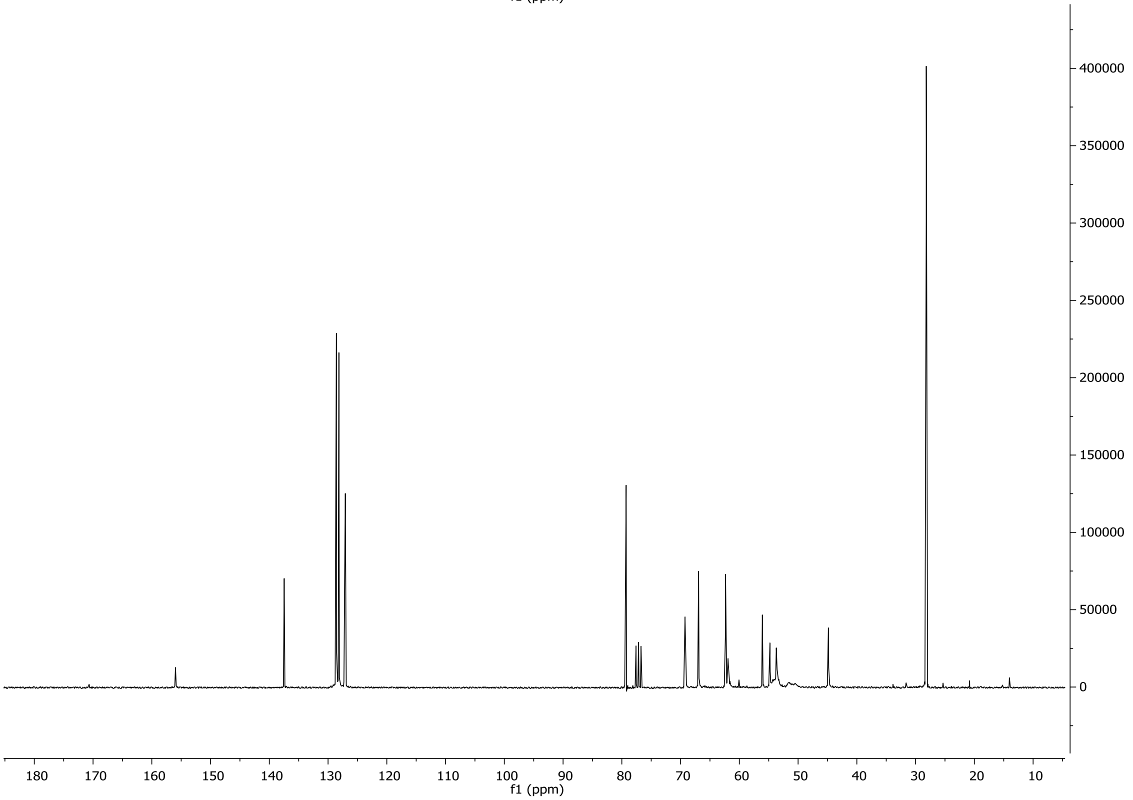

Supplement: Supplementary file 1 [file Data_Sheet_1.PDF]
